# Exploring the relationship between polycystic ovarian syndrome, testosterone, and multiple sclerosis in women: A nationwide cohort study and genome-wide cross-trait analysis

**DOI:** 10.1177/13524585241292802

**Published:** 2024-11-06

**Authors:** Yuan Jiang, Carolyn E Cesta, Qianwen Liu, Elaine Kingwell, Pernilla Stridh, Klementy Shchetynsky, Tomas Olsson, Ingrid Kockum, Elisabet Stener-Victorin, Xia Jiang, Ali Manouchehrinia

**Affiliations:** Department of Clinical Neuroscience, The Karolinska Neuroimmunology & Multiple Sclerosis Centre, Centre for Molecular Medicine, Karolinska Institutet, Stockholm, Sweden; Centre for Pharmacoepidemiology, Department of Medicine, Karolinska Institutet, Stockholm, Sweden; Department of Clinical Neuroscience, The Karolinska Neuroimmunology & Multiple Sclerosis Centre, Centre for Molecular Medicine, Karolinska Institutet, Stockholm, Sweden; Research Department of Primary Care & Population Health, University College London, London, UK; Department of Clinical Neuroscience, The Karolinska Neuroimmunology & Multiple Sclerosis Centre, Centre for Molecular Medicine, Karolinska Institutet, Stockholm, Sweden; Department of Clinical Neuroscience, The Karolinska Neuroimmunology & Multiple Sclerosis Centre, Centre for Molecular Medicine, Karolinska Institutet, Stockholm, Sweden; Department of Clinical Neuroscience, The Karolinska Neuroimmunology & Multiple Sclerosis Centre, Centre for Molecular Medicine, Karolinska Institutet, Stockholm, Sweden; Department of Clinical Neuroscience, The Karolinska Neuroimmunology & Multiple Sclerosis Centre, Centre for Molecular Medicine, Karolinska Institutet, Stockholm, Sweden; Department of Physiology and Pharmacology, Karolinska Institutet, Stockholm, Sweden; Department of Clinical Neuroscience, The Karolinska Neuroimmunology & Multiple Sclerosis Centre, Centre for Molecular Medicine, Karolinska Institutet, Stockholm, Sweden; Department of Clinical Neuroscience, The Karolinska Neuroimmunology & Multiple Sclerosis Centre, Centre for Molecular Medicine, Karolinska Institutet, Stockholm, Sweden

**Keywords:** Polycystic ovary syndrome, sex hormone, multiple sclerosis, cohort study, genome-wide cross-trait analysis, genomics

## Abstract

**Background:**

Women have a higher risk of developing multiple sclerosis (MS), potentially due to hormonal factors. Elevated testosterone levels, common in polycystic ovary syndrome (PCOS), might influence MS risk.

**Objective:**

To investigate the relationship between PCOS, as a proxy for elevated testosterone levels, and MS risk through phenotypic and genomic analysis.

**Methods:**

Cox regression models analysed the association between PCOS and MS risk. The genome-wide cross-trait analysis examined the genetic architecture.

**Results:**

In a Swedish cohort of 1,374,529 women, 77 (0.3%) with PCOS and 3,654 (0.3%) without PCOS were diagnosed with MS. After adjusting for birth year and obesity, no association was found between PCOS and MS (*HR* = 0.91, 95% CI = 0.72–1.15), which was confirmed by Mendelian randomization analysis, where genetically predicted PCOS propensity, sex hormone-binding globulin (SHBG), or testosterone levels did not causally affect MS risk (all *p*-values > 0.05). By exploring horizontal pleiotropy, we identified shared genetic regions and 19 independent pleiotropic SNPs for SHBG with MS and 11 for testosterone with MS.

**Conclusion:**

We did not find evidence for a causal role of PCOS, as a proxy of elevated testosterone, in reducing the risk of MS in women. The shared genetic loci between testosterone, SHBG, and MS provide biological insights.

## Introduction

Women are at two to three times higher risk of developing multiple sclerosis (MS), an autoimmune condition of the central nervous system.^1^ While the exact reason for female preponderance is not entirely clear, sex hormones and their disruption in homeostasis may be involved.^2^ Besides estradiol, the primary female sex hormone, the role of testosterone in females has been gaining increasing attention in recent years. Testosterone has anti-inflammatory and immunosuppressive effects and could play a role in reducing neurodegeneration, stimulating myelin formation, and mediating regeneration in MS.^2^ However, the effects of testosterone on MS susceptibility have only been demonstrated in animal experiments;^3^ evidence for consistent effects is still lacking from population-based studies, especially for women.

**Figure 1.**
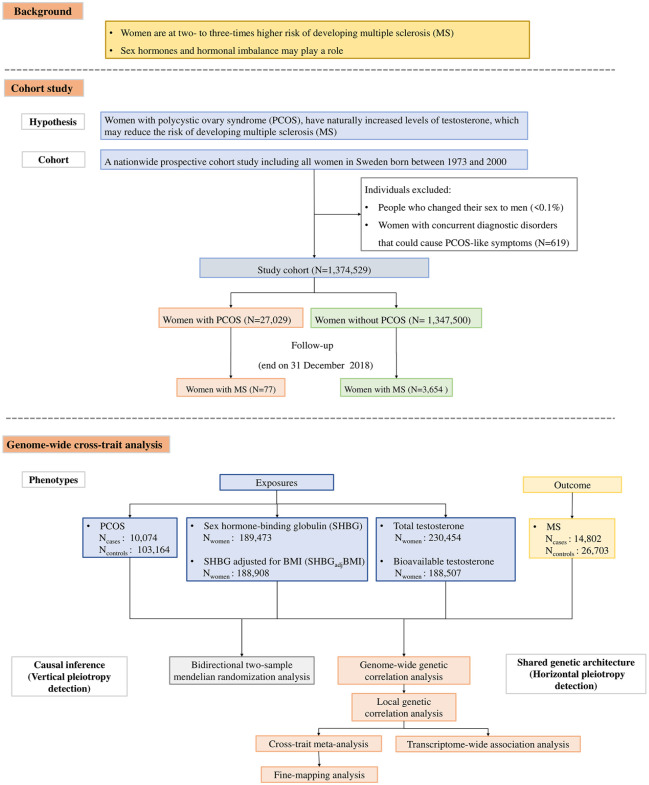
Flowchart of the overall study design. We conducted a large-scale prospective cohort study to investigate the association between polycystic ovary syndrome (PCOS), a proxy for hormonal imbalance with elevated androgen levels, and multiple sclerosis. We further validated our findings with genetic approaches by conducting a bidirectional Mendelian randomization analysis to make causal inferences between PCOS, sex hormone-binding globulin, testosterone, and MS. To investigate the shared genetic architecture underlying these traits, we first quantified the genome-wide genetic correlation between these paired traits; we then partitioned the genome into linkage-disequilibrium independent blocks to estimate local genetic correlation. Next, we identified potential pleiotropic loci contributing to both traits and pinpointed causal variants by statistical fine-mapping. Finally, we conducted transcriptome-wide association analysis to identify shared tissue-specific genes associated with both traits.

There is a limited number of reports on testosterone in women with MS, possibly due to the challenges and insensitivity of testing approaches in detecting lower levels of androgen typically found in women.^4^ However, a small cohort study in women of reproductive age (16 MS and 30 controls) demonstrated significantly lower testosterone levels in the women with MS (follicular phase: 0.46 vs 0.82 ng/mL; luteal phase: 0.58 vs 0.88 ng/mL).^5^ This finding was confirmed by two more studies: one involving 36 individuals (follicular phase: 0.49 vs 0.58 ng/mL; luteal phase: 0.47 vs 0.63 ng/mL)^6^ and the other involving 30 individuals (0.54 vs 0.75 ng/mL),^7^ while the opposite was found in a study involving 14 MS and 14 controls, where bioavailable testosterone concentration was significantly higher in premenopausal women with MS (0.018 vs 0.011 nmol/L).^8^ The inconsistent results may be due to the small sample sizes or factors often encountered in observational studies, such as selection bias or uncontrolled confounding. Women with polycystic ovary syndrome (PCOS) have naturally elevated levels of androgens throughout their reproductive life,^9,10^ which may reduce the risk of MS. To investigate the potential role of testosterone and hormonal imbalance in the development of MS, we conducted a large-scale prospective population-based cohort study to estimate the association between PCOS (as a proxy for sex hormonal imbalance characterized by elevated testosterone levels) and MS susceptibility. In addition, data from large-scale genome-wide association studies (GWASs), and cutting-edge statistical genetics approaches, allowed us to complement the phenotypic correlation by exploring their intrinsic link. Leveraging GWAS summary statistics for PCOS, sex hormone-binding globulin (SHBG), testosterone, and MS, we sought to understand the shared genetic architecture among these traits by exploring both vertical (genetic variant influencing one trait via its effects on the other) and horizontal (genetic variant having effects on both traits) pleiotropy. The overall flow of the study design is shown in Figure 1.

## Material and methods

### Epidemiological investigation

#### Data Sources

All women born in Sweden between 1973 and 2000 were identified and linked to nationwide population health registers. Detailed descriptions of the data sources, exposure classification, and outcome classification are provided in the Supplementary Methodology section.

#### Statistical analysis

Women were followed from their 13th birthday (Tanner stage 2 and 3 of puberty) to the date of diagnosis of MS, death, emigration, or end of follow-up (31 December 2018), whichever came first. The association between PCOS and MS was estimated as a hazard ratio with 95% confidence intervals (*CI*) using Cox regression models with age as the underlying time scale and adjusted for year of birth in four seven-year categories and any lifetime diagnosis of obesity. Robust standard errors were used to account for dependence between observations, which occurs when the women in the population are siblings.

A sensitivity analysis was conducted by restricting the cohort to women with complete follow-up to at least 40 years of age by the end of the study period. This approach ensured a sufficient follow-up time to minimize the potential for exposure misclassification (i.e. assuming all PCOS cases in the cohort will have been diagnosed by age 40). A *p*-value of 0.05 was used to define statistical significance. Analyses were conducted using Stata version 17.

### Genome-wide cross-trait analysis

The genome-wide cross-trait analysis provides insights into the intrinsic link underlying hormonal imbalance and MS. We first performed a bidirectional Mendelian randomization (MR) analysis to determine causal relationships between PCOS, SHBG, testosterone, and MS. To further investigate the shared genetic architecture underlying trait pairs, we quantified the genome-wide and local genetic correlations. Furthermore, we identified potential pleiotropic loci influencing both traits, which were complementarily validated through fine-mapping and a transcriptome-wide association study (TWAS).

Detailed descriptions of the GWAS summary statistics and procedures of genome-wide cross-trait analysis are provided in the Supplementary Methodology section. The characteristics of index SNPs used for MR analysis are presented in Supplementary Tables 1–5.

## Results

### PCOS and risk of MS – results from the cohort study

A total of 1,374,529 women born in Sweden between 1973 and 2000 were identified, of which 27,029 (2.0%) had a PCOS diagnosis by the end of follow-up (31 December 2018). Women with a PCOS diagnosis more often had a diagnosis of obesity than women without PCOS. MS was observed in 3,654 (0.3%) women without PCOS and in 77 (0.3%) women with PCOS. The mean age at the end of follow-up was 32 years (range 18–46) for the full cohort; age at MS diagnosis did not differ between women with (28.8 ± 6.1) and without (28.9 ± 6.4) PCOS (Table 1). We did not observe an association between PCOS and MS in either the unadjusted or adjusted models, with a hazard ratio of 0.91 (95% CI = 0.72–1.15) after adjusting for birth year and any lifetime diagnosis of obesity. Restricting to women with full follow-up to their 40th birthday (*N*_total_ = 255,480, *N*_women with PCOS_ = 4,079, *N*_women with MS_ = 1,215) (Supplementary Table 6), the adjusted hazard ratio was 0.80 (95% CI = 0.48–1.33).

**Table 1.** Characteristics of women born in Sweden between 1973 and 2000 with or without a diagnosis of polycystic ovary syndrome.

	Women with PCOS (*N* = 27,029)	Women without PCOS (*N* = 1,347,500)	Total (*N* = 1,374,529)
Year of Birth (N, %)			
1973–1979	5,486 (20.3)	335,137 (24.9)	340,623 (24.8)
1980–1986	8,769 (32.4)	312,736 (23.2)	321,505 (23.4)
1987–1993	9,601 (35.5)	384,933 (28.6)	394,534 (28.7)
1994–2000	3,173 (11.7)	314,694 (23.4)	317,867 (23.1)
**Obesity (N, %)**	5,169 (19.1)	67,390 (5.0)	72,559 (5.3)
**MS (N, %)**	77 (0.3)	3,654 (0.3)	3,731 (0.3)
**Age at MS diagnosis (mean** **±** **SD)**	28.8 ± 6.1	28.9 ± 6.4	28.9 ± 6.4
**Age at end of follow-up (mean** **±** **SD)**	32.9 ± 6.5	32.1 ± 8.0	32.1 ± 8.0

PCOS: polycystic ovary syndrome; MS: multiple sclerosis; SD: standard deviation.

### Bidirectional Mendelian randomization analysis

**Figure 2.**
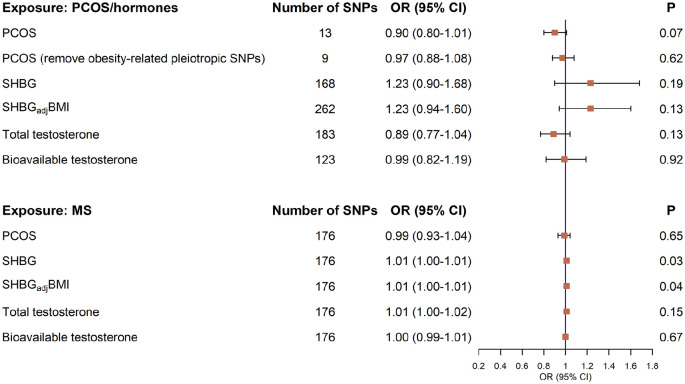
A bidirectional Mendelian randomization analysis on the causal associations between genetically predicted PCOS, SHBG, testosterone and the risk of MS. Red squares represent odds ratio of outcomes per each standard deviation increment in genetically predicted exposures. Bars represent 95% confidence intervals. The Mendelian randomization analysis was performed based on the inverse variance weighted approach. PCOS: polycystic ovary syndrome; SHBG: sex hormone-binding globulin; SHBG_adj_BMI: sex hormone-binding globulin adjusted for BMI; MS: multiple sclerosis; SNP: single nucleotide polymorphism; OR: odds ratio; CI: confidence interval.

We performed a bidirectional two-sample MR to validate the phenotypic correlation. Consistent with the result from the cohort study, no significant causal relationship was observed between PCOS and the risk of MS (IVW *OR* = 0.90, 95% CI = 0.80–1.01, *p* = 0.07). The estimate remained unchanged after removing obesity-related SNPs (IVW *OR* = 0.97, 95% CI = 0.88–1.08, *p* = 0.62) (Figure 2, Supplementary Table 7).

We next explored the causal relationship between levels of SHBG, testosterone, and MS risk and found an overall null association (IVW SHBG *OR* = 1.23, 95% CI = 0.90–1.68, *p* = 0.19; SHBG_adj_BMI *OR* = 1.23, 95% CI = 0.94–1.60, *p* = 0.13; total testosterone *OR* = 0.89, 95% CI = 0.77–1.04, *p* = 0.13; bioavailable testosterone *OR* = 0.99, 95% CI = 0.82–1.19, *p* = 0.92) (Figure 2, Supplementary Table 7). These results remained consistent in all sensitivity analyses (Supplementary Table 7). No evidence of reverse causality was observed, indicating that genetic predisposition for MS did not affect the risk of PCOS or sex hormone levels (Figure 2, Supplementary Table 8).

Under the sample size of MS (41,505 with 35.66% of MS cases) and assuming an α of 0.05, given that the phenotypic variance explained by exposure-associated IVs ranged from 0.005 to 0.145, our study had 80% power to detect a 47% change in the risk of MS associated with PCOS. The corresponding effect sizes were 8%–10% for SHBG and 12%–15% for testosterone (Supplementary Table 9).

### Genome-wide and local genetic correlations

**Figure 3.**
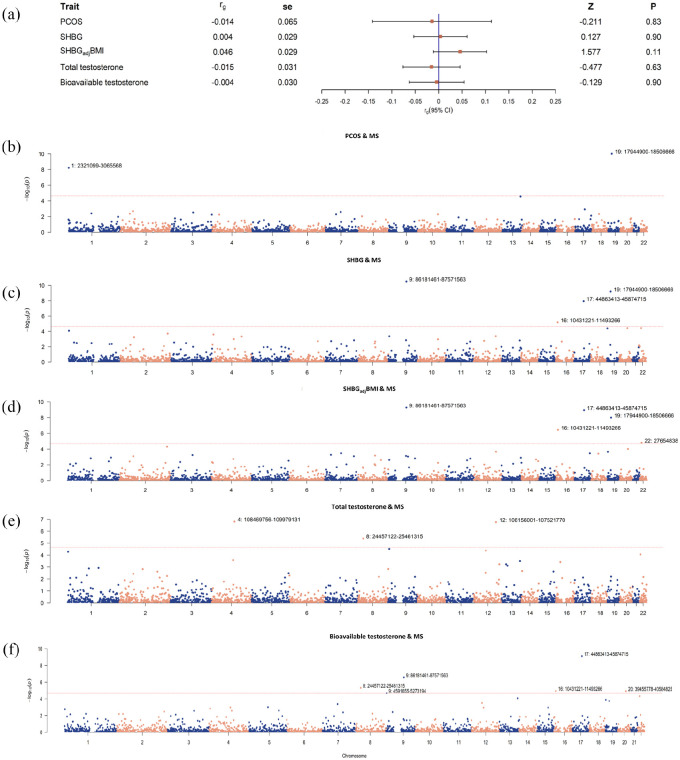
Genome-wide genetic correlation and local genetic covariance between PCOS, SHBG, testosterone, and MS. (a) Estimates of genome-wide genetic correlation between PCOS, SHBG, testosterone, and multiple sclerosis (MS). Squares represent point estimates of genetic correlation, and bars represent standard error. (b-f) local genetic covariance between PCOS, SHBG, testosterone, and MS. X-axis represents chromosomes, Y-axis represents negative logarithm of *p*-values, and each dot in the Manhattan plot represents a linkage-disequilibrium independent genomic region. Chromosomes and genomic regions of significant local genetic correlations (*p* < 2263/0.05) were marked. SHBG: sex hormone-binding globulin; SHBG_adj_BMI: sex hormone-binding globulin adjusted for BMI; PCOS: polycystic ovary syndrome; MS: multiple sclerosis.

We further explored the horizontal pleiotropy among pairs of traits. Despite a negligible genome-wide genetic correlation across PCOS, SHBG, testosterone, and MS (all *p*-values > 0.05) (Figure 3(a)), significant local correlations were observed. For PCOS with MS, two significant local regions were identified (1p36.32, 19p13.11). There were five local regions for SHBG with MS (four by using crude SHBG: 9q21.32-q21.33, 16p13.2-p13.13, 17q21.31-q21.32, 19p13.11; an additional one by using SHBG_adj_BMI: 22q12.1), and eight for testosterone with MS (three by using total testosterone: 4q25, 8p21.2, 12q23.3; an additional five by using bioavailable testosterone: 9p24.2-p24.1, 9q21.32-q21.33, 16p13.2-p13.13, 17q21.31-q21.32, 20q12) (Figure 3(b)–(f), Supplementary Table 10).

### Cross-trait meta-analysis

Motivated by the significant local genetic correlations, we conducted a cross-trait meta-analysis to simultaneously identify pleiotropic SNPs affecting PCOS, SHBG, testosterone, and MS. After excluding MHC regions due to their complex LD patterns, no pleiotropic SNP was identified for PCOS with MS.

**Figure 4.**
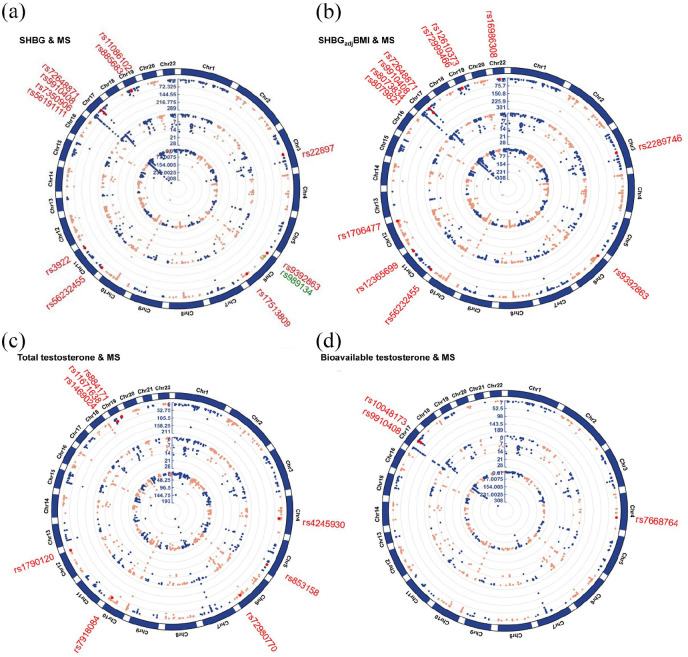
Cross-trait meta-analysis between SHBG, testosterone, and MS. (a) Cross-trait meta-analysis between SHBG and MS. (b) Cross-trait meta-analysis between SHBG_adj_BMI and MS. (c) Cross-trait meta-analysis between total testosterone and MS. (d) Cross-trait meta-analysis between bioavailable testosterone and MS. We did not find pleiotropic SNP of PCOS with MS. Independent top-associated loci of sex hormones and multiple sclerosis are located in inner and middle circles of the circular Manhattan plot, respectively. Results of cross-trait meta-analyses are listed in outer circles. Significant pleiotropic SNPs are presented by red spot. A novel pleiotropic SNP was detected for SHBG with MS, marked by a green spot (rs989134). Chromosomes are located outside of the circle. The scales inside the circles represent the negative logarithm of *p*-values. Each dot in the circular Manhattan plot represents an independent top-associated locus. MHC regions were excluded due to their complex LD pattern. Chr: chromosome; SHBG: sex hormone-binding globulin; SHBG_adj_BMI: sex hormone-binding globulin adjusted for BMI; MS: multiple sclerosis.

As shown in Figure 4 and Supplementary Table 11, for SHBG with MS, a total of 19 independent pleiotropic SNPs were identified (12 by using crude SHBG and an additional seven by using SHBG_adj_BMI). In addition to the five ‘known’ SNPs, five ‘SHBG-driven’ SNPs, one ‘MS-driven’ SNP, and seven ‘LD-tagged’ SNPs, one novel pleiotropic SNP (rs989134) was detected, which is an intron variant located in chromosome 6. For each of these 19 SNPs, we further determined a 95% credible set of causal SNPs through fine-mapping analysis. We found two SNPs to be highly likely causal (rs9910408 and rs72648871, with a posterior probability of 1.00) (Supplementary Table 12). When exploring gene-level association using TWAS, we identified ten pleiotropic genes, eight of which were also mapped by SNPs detected in our cross-trait meta-analysis (GFI1, KPNB1, NPEPPS, TBKBP1, AC025682.1, AC068234.2, MAST3, MPV17 L2, PDE4 C, and OGFOD2) (Supplementary Table 13).

For testosterone with MS, a total of 11 independent pleiotropic SNPs were identified (eight by using total testosterone and an additional three by using bioavailable testosterone). Among these, no novel pleiotropic SNP was detected (two ‘known’ SNPs, three ‘testosterone-driven’ SNPs, one ‘MS-driven’ SNP, and five ‘LD-tagged’ SNPs). Fine-mapping analysis identified SNP rs9910408 as highly likely causal (posterior probability of 1.00). Using TWAS analysis, we identified six pleiotropic genes, three of which were also mapped by SNPs detected in our cross-trait meta-analysis (KPNB1, NPEPPS, and TBKBP1) (Supplementary Table 11–13).

## Discussion

Our study, incorporating analyses of both phenotypes and genetics, represents the most comprehensive investigation to date on the link between testosterone and MS in women. We observed no association between PCOS (as a proxy for elevated testosterone levels) and the development of MS from a phenotypic perspective. Our genetic analysis revealed notable biological links, with horizontal pleiotropy playing a more important role than vertical pleiotropy, which advances our understanding of the complex relationships underlying testosterone and MS.

Previous *in vitro* and *in vivo* experiments suggested the greater susceptibility in females is partly due to a protective effect of androgens.^11^ However, this protective role has not been confirmed through population-based studies, especially among women, due to the limited sample sizes and difficulties in accurately measuring fluctuating sex hormone levels.^5,6,8,12^ In our study, using unprecedentedly large samples, we could not detect any evidence of a causal association between naturally increased testosterone and MS development in women. The credibility of this finding can be attributed to several factors. First, the absence of association was observed through both an observational study and a large-scale MR analysis. Our MR analysis included not only PCOS as a proxy for elevated androgens but also directly investigated the role of testosterone in MS. The MR approach overcomes many potential disadvantages of conventional observational epidemiology, such as confounding, reverse causation, and measurement errors, and enhances the validity of our epidemiological findings. Second, the robustness and reliability of our findings were further supported by a series of sensitivity analyses conducted in both the observational and genetic analyses. Third, in the observational setting, we used data from a cohort of all women in Sweden, which minimized selection bias and strengthened the generalizability of our findings.

Although, according to our study, elevated testosterone is most likely not associated with the development of MS in women, several other factors may provide explanations for the sex disequilibrium of MS development. Oestrogen and progesterone, the primary female sex hormones, may play a role. Oestrogen has been suggested to have a neuroprotective effect, as its intrinsic antioxidant properties may help to provide a chemical shield for neurons.^13^ Moreover, it has also been found to affect immune tolerance, with a paradoxical effect of suppressing inflammation and generating a pro-inflammatory effect.^14^ However, although women with PCOS may also have elevated levels of oestrogen,^10^ an effect of PCOS on MS risk has not been identified through our study. Progesterone has been shown to have anti-inflammatory^14^ and neuroprotective properties and is seen as a promising candidate for promoting myelination.^15^ The combination of oestrogen and progesterone in experimental autoimmune encephalomyelitis (EAE) has been shown to inhibit the development of major neurochemical abnormalities and clinical signs of the disease.^16^ This suggests that multiple sex hormones, rather than a single one, could be involved in the development of MS. In addition to hormones, sex-linked genes may contribute to sex differences in MS. The X chromosome gene *FoxP3* elicits downstream immunosuppressive responses by regulating the development and function of T regulatory cells. Differences in methylation between sexes lead to more *FoxP3* expression in males compared to females, which would be consistent with the phenomenon of less autoimmunity in males; the X chromosome gene *TLR7* has been shown to mediate neuronal degeneration in cortical neurons, which was found to be higher in male mice compared with female mice in central nervous system.^17^ Moreover, the latest GWAS revealed that the MS locus (rs2807267) on the X chromosome is associated with MS susceptibility.^18^ Moreover, sex differences in immune responses, epigenetics, and environmental and metabolic factors may also play roles.^2,15,19,20^ A study suggested that sex hormone effects may depend not only on their concentration but also on the type of target cell and the receptor subtype expressed on a given cell type.^14^ Since MS susceptibility is likely influenced by multiple factors, it is important to consider how these factors may interact with each other in future studies.

Our genetic analysis revealed the important role of horizontal pleiotropy. Here, we highlight several interesting findings. The genes *GFI1, KPNB1, NPEPPS*, and *TBKBP1* are shared between SHBG and MS, as well as between testosterone and MS. *GFI1*, a protein-coding gene located in 1p22.1, regulates gene expression by blocking transcription and is essential for CD4 ^+^ cell function.^21^ *KPNB1*, located in 17q21.32, plays a role in NF-κB signalling, an important process for immune cell activation in response to infection or inflammation.^21^ *NPEPPS* located in 17q21.32, participates in the innate immune system pathway and contributes to proteolytic events that regulate the cell cycle. *TBKBP1*, a protein-coding gene located in 17q21.32, is involved in NF-κB signalling and many cellular processes such as immune response and inflammation.^22^ While the direct link between these genes and sex hormones has not been found, it is likely that they could indirectly influence hormone levels or activities through various cellular processes. The discovery of underlying shared mechanisms of these diseases could contribute to the development of prevention strategies and treatment options for MS in the future.

Our findings should be interpreted with caution. First, we used PCOS as a proxy for elevated testosterone. Testosterone levels fluctuate significantly throughout the lifetime of women due to factors such as age, menstrual cycle, health status, and lifestyle. Therefore, using PCOS-proxied high testosterone levels may not accurately detect a link between testosterone and MS at specific periods. Although accurately measuring testosterone levels in women remains a challenge, direct measurements would be needed to validate our findings in the future. Second, we observed a 2% prevalence of PCOS in our cohort, lower than the estimated 5%–15%,^23^ likely attributable to the younger mean age of the study cohort at the end of follow-up and changes in diagnostic criteria over the years. In addition, undiagnosed cases of PCOS and cases only diagnosed in primary health care, which are not captured in the national registers, would have been treated as non-PCOS. Such misclassification could have led to an underestimation of the association between exposure and outcome. Nevertheless, considering that the diagnosis of PCOS requires meeting at least two out of three diagnostic criteria, the phenotypes with hyperandrogenemia, which is one of the criteria and exhibits more severe symptoms, are more likely to be diagnosed and treated in specialist care (e.g. by an endocrinologist) and therefore captured in the register data. This may result in the PCOS cases in our study being more suitable as a proxy for sex hormonal imbalance with elevated androgens. Third, our study used the date of first clinical recognition of MS, which often occurs later than MS symptom onset. The delay between MS onset and clinical recognition may have resulted in under ascertainment of MS outcomes in our study. Fourth, in our genetic analysis, we used female-specific GWAS data for PCOS and sex hormones, but sex-combined GWAS data for MS. While this may introduce sex heterogeneity into our analysis, we expected the effect to be small since MS is a female-dominant trait. To assess the robustness of the findings though, we performed sensitivity analyses using sex-combined data of exposures and obtained similar results (not shown). Fifth, we excluded the MHC regions from our analyses due to their complex LD pattern. Although the MHC region plays an important role in the immune system and exerts a strong genetic effect on MS risk, no direct link between the MHC and testosterone has been detected. Therefore, we assumed that removing the MHC region from the analyses would not significantly impact our findings. Sixth, although our study focuses on the potential effect of testosterone on the development of MS, testosterone can be converted to estradiol, the primary female sex hormone, by aromatase.^2^ An investigation of the role of estradiol in women within a large-scale population could provide valuable insights into the effects of other sex hormones on MS susceptibility. While our current study lacks direct measurement of sex hormones, future studies could delve deeper into this aspect. Moreover, it would also be important to explore the effects of sex hormones on the activity and progression of MS. Finally, our observational study was performed in a Swedish population, and the genomic analyses were performed in populations of European ancestry; observations may therefore only be generalizable to European populations. The findings of our study should be verified in other populations.

## Conclusion

We did not find evidence to support a causal role of PCOS, as a proxy of elevated testosterone, in reducing the risk of MS in women. However, the identification of shared genetic loci provides valuable biological insights, which may help guide future research and provide evidence for MS prevention and treatment.

## Supplemental Material

sj-docx-1-msj-10.1177_13524585241292802 – Supplemental material for Exploring the relationship between polycystic ovarian syndrome, testosterone, and multiple sclerosis in women: A nationwide cohort study and genome-wide cross-trait analysis Supplemental material, sj-docx-1-msj-10.1177_13524585241292802 for Exploring the relationship between polycystic ovarian syndrome, testosterone, and multiple sclerosis in women: A nationwide cohort study and genome-wide cross-trait analysis by Yuan Jiang, Carolyn E Cesta, Qianwen Liu, Elaine Kingwell, Pernilla Stridh, Klementy Shchetynsky, Tomas Olsson, Ingrid Kockum, Elisabet Stener-Victorin, Xia Jiang and Ali Manouchehrinia in Multiple Sclerosis Journal

sj-docx-2-msj-10.1177_13524585241292802 – Supplemental material for Exploring the relationship between polycystic ovarian syndrome, testosterone, and multiple sclerosis in women: A nationwide cohort study and genome-wide cross-trait analysis Supplemental material, sj-docx-2-msj-10.1177_13524585241292802 for Exploring the relationship between polycystic ovarian syndrome, testosterone, and multiple sclerosis in women: A nationwide cohort study and genome-wide cross-trait analysis by Yuan Jiang, Carolyn E Cesta, Qianwen Liu, Elaine Kingwell, Pernilla Stridh, Klementy Shchetynsky, Tomas Olsson, Ingrid Kockum, Elisabet Stener-Victorin, Xia Jiang and Ali Manouchehrinia in Multiple Sclerosis Journal
