# Supplementary material for: Exploring the relationship between polycystic ovarian syndrome, testosterone, and multiple sclerosis in women: A nationwide cohort study and genome-wide cross-trait analysis
Source: Mult Scler. 2024 Nov 6;30(14):1765–74. doi: 10.1177/13524585241292802 (PMC11616213; doi:10.1177/13524585241292802)
Supplement: sj-docx-1-msj-10.1177_13524585241292802 – Supplemental material for Exploring the relationship between polycystic ovarian syndrome, testosterone, and multiple sclerosis in women: A nationwide cohort study and genome-wide cross-trait analysis [file sj-docx-1-msj-10.1177_13524585241292802.docx]

**Supplementary Methodology**

**Epidemiological investigation**

**Data sources** All women born in Sweden between 1973 and 2000 were identified and linked to nationwide population health registers by the unique personal identification number assigned to each individual at birth. Individuals born female but registered a sex change were excluded from the study population (<0.1%). Data from the National Patient Register (NPR), Migration Register, and Cause of Death Register were extracted. The NPR covers all public inpatient care since 1987 and all hospital-based specialist outpatient care since 2001.

**Exposure classification** Women with PCOS were identified by having at least one recorded diagnosis of PCOS in the NPR after 1990 and after the age of 13 (ICD-9: 256E; ICD-10: E28.2). Given that women with PCOS have elevated androgen levels throughout their reproductive lives^1, 2^, we assume that elevated androgen levels begin at puberty, regardless of the timing of the PCOS diagnosis, which often occurs during the pregnancy preparatory period when they seek medical care for infertility.

Women with a concurrent diagnosed condition that could cause symptoms similar to PCOS were considered as not having PCOS to ensure specificity (N=619), including pituitary adenoma (ICD-10: D35.2), hyperfunction of the pituitary glands (ICD-10: E22), Cushing’s syndrome (ICD-10: E24), adrenogenital disorders including congenital adrenal hyperplasia (ICD-10: E25), other disorders of the adrenal glands (ICD-10: E27), and Turner’s syndrome (ICD-10: O96).

**Outcome classification** Women with MS were identified by having at least two recorded ICD-10 G35 diagnoses in the NPR.^3^ The date of the first ICD-10 code for MS (diagnosis) was used to define age at diagnosis.

**Covariates** Any lifetime diagnosis of obesity was identified from the NPR by ICD-10 code E66.

**Genome-wide cross-trait analysis**

**PCOS GWAS** Summary statistics for the risk of PCOS were obtained from a meta-analysis of GWASs comprising 10,074 PCOS cases and 103,164 controls of European ancestry ^4^. Genetic variants with minor allele frequency (MAF) < 1%, imputation quality (*R^2^*) < 0.3, or imputation information score (info) < 0.4 were excluded. A total of 14 genome-wide significant SNPs (*P* < 5×10^-8^) were identified.

**SHBG GWAS** SHBG is a protein which plays an important role in transportation and regulation of testosterone by binding to it. High levels of SHBG can reduce the levels of bioavailable testosterone in the body, and vice versa. The latest and largest GWAS of circulating SHBG levels was conducted by UK Biobank, involving 189,473 women of European ancestry. Genetic variants with MAF < 1% were excluded. A total of 237 genome-wide significant SNPs (*P* < 5×10^-8^) were identified using 1Mb distance-based clumping with linkage disequilibrium (LD) < 0.05 across all variants ^5^. To account for the confounding effect of BMI, we also used summary statistics of BMI-adjusted SHBG (SHBG_adj_BMI), which involved 188,908 women and identified a total of 359 index SNPs.^5^

**Testosterone GWAS** GWAS summary statistics of total testosterone levels involved 230,454 women of European ancestry. The study population, genetic discovery process, and signal selection standard were identical to those of SHBG GWAS. A total of 254 index SNPs were identified. The level of bioavailable testosterone was calculated by using Vermeulen Equation and involved 188,507 women. A total of 180 index SNPs were identified ^5^.

**MS GWAS** Summary statistics for the risk of MS were obtained from a recently published GWAS meta-analysis including 14,802 MS cases and 26,703 non-MS controls of non-Hispanic white population from 15 participating data sets^6^. Each of the 15 participating data sets was first quality-checked and then pooled through a fixed-effects meta-analysis. A total of 200 SNPs in the autosomal non-major histocompatibility complex (MHC) genome were obtained with genome-wide significance (*P* < 5×10^-8^). Among this GWAS, one participating data set with 691 MS patients was from Sweden. Consequently, there may be some overlap with the participants in our observational study(<3%). Nevertheless, given that both datasets are derived from populations of European ancestry, the risk of spurious associations due to population structure is likely minimized.

**Bidirectional mendelian randomization analysis** MR analysis elucidates a putative exposure-outcome causal association by using genetic variants (SNPs) as instrumental variables (IVs) ^7^. A two-sample bidirectional MR analysis was performed to determine the causal association between PCOS, sex hormones, and MS. The inverse variance weighted (IVW) approach was used as our primary approach, assuming all IVs were valid, or the overall pleiotropy was balanced to zero ^8^. A series of sensitivity analyses were conducted to validate model assumptions and to guarantee the robustness of findings. MR-Egger regression was applied under the assumption of instrument strength independent of direct effects, where the intercept term reflects directional pleiotropy ^9^. The weighted median approach was performed assuming that up to 50% of IVs contributing to the analysis were invalid ^10^. The IVW approach was repeated, excluding palindromic SNPs (A/T or G/C SNPs introducing ambiguity into identifying effect alleles). As PCOS can lead to both hyperandrogenism and obesity, to minimize the confounding effect, MR analysis was performed using IVs without obesity-related pleiotropic SNPs.

Statistical power was calculated using the non-centrality parameter-based approach ^11^. All analyses were conducted using packages “TwoSampleMR”, “MRInstruments”, and “Mendelian Randomization” in R v3.6.3. P < 0.05 was used to define statistical significance.

**Genetic correlation analysis** To further investigate the shared genetic architecture, genome-wide genetic correlation ($r_{g}$) was quantified using cross-trait LD-score regression (LDSC), an algorithm that quantifies the average sharing of genetic effects between pairs of traits ^12, 13^. A threshold of P < 0.05 was established to define statistical significance. Local genetic covariance was quantified using SUPERGNOVA (Super GeNetic cOVariance Analyzer) to identify specific regions to disproportionately affect both traits ^14^. A total of 2,263 LD-independent blocks were determined by using the 1000 Genomes project reference panel, and a Bonferroni-corrected P-value of 0.05/2,263 was used to define statistical significance.

**Cross-trait meta-analysis** To further detect potential pleiotropic loci influencing both traits, a cross-trait meta-analysis was applied using cross-phenotype association analysis (CPASSOC) ^15^. The test statistic S_Het_ was used to combine association evidence.

Independent top-associated loci were obtained by applying the default PLINK LD-based clumping function (parameters: --clump-p1 5e-8 --clump-p2 1e-5 --clump-r2 0.2 --clump-kb 500). Significant pleiotropic SNPs were defined as variants with P_single-trait_ < 1×10^-5^ in both traits and P_CPASSOC_ < 5×10^-8^ in paired traits. These SNPs were further divided into four categories. First, we identified a “known” pleiotropic SNP that reached genome-wide significance in both single traits (P_PCOS/sex hormone_ < 5×10^-8^ and P_MS_ < 5×10^-8^). These SNPs were naturally pleiotropic even without performing CPASSOC. Second, we identified a “single-trait-driven” pleiotropic SNP that reached genome-wide significance in one of two single traits (P_PCOS/sex hormone_ < 5×10^-8^ or P_MS_ < 5×10^-8^ and P_CPASSOC_ < 5×10^-8^). Third, we identified an “LD-tagged” pleiotropic SNP that, despite not reaching genome-wide significance in any single trait (5×10^-8^ < P_PCOS/sex hormone_ < 1×10^-3^, 5×10^-8^ < P_MS_ < 1×10^-3^, and P_CPASSOC_ < 5×10^-8^), was in LD (r^2^-threshold=0.2) with index SNPs (or any SNP located within ± 250K around the index SNPs) identified by single-trait GWAS(s). Finally, a “novel” pleiotropic SNP, that we were most interested in, defined as those that neither reached genome-wide significance in single trait nor in LD with previously identified SNPs for single-trait GWAS(s). Ensembl Variant Effect Predictor was used to map pleiotropic SNPs to genes ^16^.

**Fine-mapping analysis and transcriptome-wide association study**

To validate identified pleiotropic SNPs and to provide biological insight, two additional analyses were conducted. First, a 95% credible set of causal variants was identified for each pleiotropic SNP using fine-mapping approach, a simplified Bayesian fine-mapping method using summary statistics ^17, 18^. Second, a TWAS was performed to identify relevant genes whose expression patterns vary across tissues. Imputable genes were provided by pre-trained joint-tissue imputation prediction models (GTEx v8) ^19^. Gene-phenotype association analysis was performed by S-PrediXcan ^19, 20^. We performed a single-trait TWAS and intersected these results to examine if they were shared across traits. Bonferroni correction was used to account for the number of gene-tissue pairs tested in each trait.

The full sets of GWAS summary statistics were obtained and used for other genetic analyses. The human reference genome build 37 (or hg19) was used for all analyses.

**References**

1. Pinola P, Piltonen TT, Puurunen J, et al. Androgen Profile Through Life in Women With Polycystic Ovary Syndrome: A Nordic Multicenter Collaboration Study. *J Clin Endocrinol Metab* 2015; 100: 3400-3407. 20150720. DOI: 10.1210/jc.2015-2123.

2. Stener-Victorin E, Holm G, Labrie F, et al. Are there any sensitive and specific sex steroid markers for polycystic ovary syndrome? *J Clin Endocrinol Metab* 2010; 95: 810-819. 20091216. DOI: 10.1210/jc.2009-1908.

3. Teljas C, Boström I, Marrie RA, et al. Validating the diagnosis of multiple sclerosis using Swedish administrative data in Värmland County. *Acta Neurol Scand* 2021; 144: 680-686. 20210806. DOI: 10.1111/ane.13514.

4. Day F, Karaderi T, Jones MR, et al. Large-scale genome-wide meta-analysis of polycystic ovary syndrome suggests shared genetic architecture for different diagnosis criteria. *PLoS genetics* 2018; 14: e1007813.

5. Ruth KS, Day FR, Tyrrell J, et al. Using human genetics to understand the disease impacts of testosterone in men and women. *Nature Medicine* 2020; 26: 252-258. DOI: 10.1038/s41591-020-0751-5.

6. Patsopoulos NA, Baranzini SE, Santaniello A, et al. Multiple sclerosis genomic map implicates peripheral immune cells and microglia in susceptibility. *Science* 2019; 365: eaav7188. DOI: doi:10.1126/science.aav7188.

7. Smith GD and Ebrahim S. 'Mendelian randomization': can genetic epidemiology contribute to understanding environmental determinants of disease? *Int J Epidemiol* 2003; 32: 1-22. DOI: 10.1093/ije/dyg070.

8. Burgess S, Butterworth A and Thompson SG. Mendelian randomization analysis with multiple genetic variants using summarized data. *Genetic epidemiology* 2013; 37: 658-665. DOI: 10.1002/gepi.21758.

9. Burgess S and Thompson SG. Interpreting findings from Mendelian randomization using the MR-Egger method. *European Journal of Epidemiology* 2017; 32: 377-389. DOI: 10.1007/s10654-017-0255-x.

10. Bowden J, Davey Smith G, Haycock PC, et al. Consistent estimation in Mendelian randomization with some invalid instruments using a weighted median estimator. *Genetic epidemiology* 2016; 40: 304-314. DOI: 10.1002/gepi.21965.

11. Brion M-JA, Shakhbazov K and Visscher PM. Calculating statistical power in Mendelian randomization studies. *International Journal of Epidemiology* 2012; 42: 1497-1501. DOI: 10.1093/ije/dyt179.

12. Bulik-Sullivan BK, Loh P-R, Finucane HK, et al. LD Score regression distinguishes confounding from polygenicity in genome-wide association studies. *Nature Genetics* 2015; 47: 291-295. DOI: 10.1038/ng.3211.

13. Bulik-Sullivan B, Finucane HK, Anttila V, et al. An atlas of genetic correlations across human diseases and traits. *Nature Genetics* 2015; 47: 1236-1241. DOI: 10.1038/ng.3406.

14. Zhang Y, Lu Q, Ye Y, et al. SUPERGNOVA: local genetic correlation analysis reveals heterogeneous etiologic sharing of complex traits. *Genome Biology* 2021; 22: 262. DOI: 10.1186/s13059-021-02478-w.

15. Zhu X, Feng T, Tayo BO, et al. Meta-analysis of correlated traits via summary statistics from GWASs with an application in hypertension. *Am J Hum Genet* 2015; 96: 21-36. 20141211. DOI: 10.1016/j.ajhg.2014.11.011.

16. McLaren W, Gil L, Hunt SE, et al. The Ensembl Variant Effect Predictor. *Genome Biology* 2016; 17: 122. DOI: 10.1186/s13059-016-0974-4.

17. Huang H, Fang M, Jostins L, et al. Fine-mapping inflammatory bowel disease loci to single-variant resolution. *Nature* 2017; 547: 173-178. DOI: 10.1038/nature22969.

18. Maller JB, McVean G, Byrnes J, et al. Bayesian refinement of association signals for 14 loci in 3 common diseases. *Nature Genetics* 2012; 44: 1294-1301. DOI: 10.1038/ng.2435.

19. Zhou D, Jiang Y, Zhong X, et al. A unified framework for joint-tissue transcriptome-wide association and Mendelian randomization analysis. *Nature Genetics* 2020; 52: 1239-1246. DOI: 10.1038/s41588-020-0706-2.

20. Barbeira AN, Dickinson SP, Bonazzola R, et al. Exploring the phenotypic consequences of tissue specific gene expression variation inferred from GWAS summary statistics. *Nature Communications* 2018; 9: 1825. DOI: 10.1038/s41467-018-03621-1.
