# Supplementary material for: Exploring the relationship between polycystic ovarian syndrome, testosterone, and multiple sclerosis in women: A nationwide cohort study and genome-wide cross-trait analysis
Source: Mult Scler. 2024 Nov 6;30(14):1765–74. doi: 10.1177/13524585241292802 (PMC11616213; doi:10.1177/13524585241292802)
Supplement: sj-docx-2-msj-10.1177_13524585241292802 – Supplemental material for Exploring the relationship between polycystic ovarian syndrome, testosterone, and multiple sclerosis in women: A nationwide cohort study and genome-wide cross-trait analysis [file sj-docx-2-msj-10.1177_13524585241292802.docx]

| **Table S1. The characteristic of PCOS-associated index SNPs and their effect sizes with exposure and outcome.** | | | | | | | | | |
| --- | --- | --- | --- | --- | --- | --- | --- | --- | --- |
| **SNP** | **Chr** | **Position** | **Allele frequency** | **A1** | **A2** | **Exposure** | | **Outcome** | |
|  |  |  |  |  |  | **beta** | **se** | **beta** | **se** |
| rs7563201 | 2 | 43561780 | 0.451 | A | G | -0.1081 | 0.0172 | 0.0299 | 0.0183 |
| rs2178575 | 2 | 213391766 | 0.151 | A | G | 0.1663 | 0.0219 | 0.0178 | 0.0226 |
| rs13164856 | 5 | 131813204 | 0.729 | T | C | 0.1235 | 0.0193 | -0.0712 | 0.0189 |
| rs804279 | 8 | 11623889 | 0.262 | A | T | 0.1276 | 0.0184 | 0.0232 | 0.0188 |
| rs10739076 | 9 | 5440589 | 0.308 | A | C | 0.1097 | 0.0197 | -0.0120 | 0.0207 |
| rs7864171 | 9 | 97723266 | 0.428 | A | G | -0.0933 | 0.0168 | -0.0048 | 0.0166 |
| rs9696009 | 9 | 126619233 | 0.068 | A | G | 0.2020 | 0.0311 | -0.0230 | 0.0333 |
| rs11031005 | 11 | 30226356 | 0.854 | T | C | -0.1593 | 0.0223 | 0.0382 | 0.0231 |
| rs11225154 | 11 | 102043240 | 0.094 | A | G | 0.1787 | 0.0272 | -0.0399 | 0.0300 |
| rs1784692 | 11 | 113949232 | 0.824 | T | C | 0.1438 | 0.0226 | -0.0172 | 0.0215 |
| rs2271194 | 12 | 56477694 | 0.416 | A | T | 0.0971 | 0.0166 | -0.0209 | 0.0171 |
| rs1795379 | 12 | 75941042 | 0.240 | T | C | -0.1174 | 0.0195 | -0.0112 | 0.0191 |
| rs8043701 | 16 | 52375777 | 0.815 | A | T | -0.1273 | 0.0208 | 0.0036 | 0.0217 |
| PCOS: polycystic ovary syndrome; SNP: single nucleotide polymorphism; Chr: chromosome; A1:effect allele; A2:other allele. | | | | | | | | | |

| **Table S2. The characteristic of SHBG-associated index SNPs and their effect sizes with exposure and outcome.** | | | | | | | | | |
| --- | --- | --- | --- | --- | --- | --- | --- | --- | --- |
| **SNP** | **Chr** | **Position** | **Allele frequency** | **A1** | **A2** | **Exposure** | | **Outcome** | |
|  |  |  |  |  |  | **beta** | **se** | **beta** | **se** |
| rs114165349 | 1 | 27021913 | 0.976296 | G | C | 0.0695 | 0.0047 | -0.0508 | 0.0592 |
| rs74090351 | 1 | 61705898 | 0.068767 | A | G | 0.0160 | 0.0028 | 0.0073 | 0.0337 |
| rs469721 | 1 | 91530001 | 0.803120 | C | T | 0.0128 | 0.0018 | -0.0364 | 0.0219 |
| rs1730862 | 1 | 107614003 | 0.341827 | G | A | 0.0208 | 0.0015 | 0.0225 | 0.0175 |
| rs267733 | 1 | 150958836 | 0.838969 | A | G | 0.0142 | 0.0019 | 0.0278 | 0.0227 |
| rs9426829 | 1 | 154592201 | 0.480729 | C | T | 0.0140 | 0.0014 | 0.0027 | 0.0162 |
| rs2064074 | 1 | 171061990 | 0.528459 | A | G | 0.0076 | 0.0014 | 0.0163 | 0.0205 |
| rs34331968 | 1 | 196659753 | 0.533525 | T | C | 0.0123 | 0.0014 | -0.0076 | 0.0164 |
| rs17583875 | 1 | 197924770 | 0.020867 | A | G | 0.0274 | 0.0049 | 0.0386 | 0.0563 |
| rs1223796 | 1 | 214323347 | 0.163998 | G | C | 0.0125 | 0.0019 | 0.0204 | 0.0223 |
| rs3001032 | 1 | 219727779 | 0.319697 | C | T | 0.0120 | 0.0015 | 0.0121 | 0.0177 |
| rs61830291 | 1 | 221001142 | 0.096820 | C | A | 0.0138 | 0.0024 | 0.0697 | 0.0348 |
| rs7567544 | 2 | 20392802 | 0.454669 | C | G | 0.0081 | 0.0014 | 0.0124 | 0.0168 |
| rs1260326 | 2 | 27730940 | 0.607415 | C | T | 0.0343 | 0.0014 | 0.0092 | 0.0166 |
| rs6736913 | 2 | 42510018 | 0.020645 | A | G | 0.0337 | 0.005 | -0.2761 | 0.3082 |
| rs6546096 | 2 | 64906295 | 0.262505 | A | G | 0.0276 | 0.0016 | -0.0151 | 0.0184 |
| rs12624244 | 2 | 70417138 | 0.935671 | A | G | 0.0208 | 0.0029 | -0.0219 | 0.0321 |
| rs12613243 | 2 | 111897506 | 0.936726 | T | C | 0.0154 | 0.0029 | -0.0402 | 0.0337 |
| rs11688682 | 2 | 121347612 | 0.270439 | C | G | 0.0092 | 0.0016 | 0.0394 | 0.0248 |
| rs1128249 | 2 | 165528624 | 0.392363 | T | G | 0.0171 | 0.0014 | -0.0095 | 0.0167 |
| rs1047891 | 2 | 211540507 | 0.315407 | A | C | 0.0142 | 0.0015 | 0.0093 | 0.0193 |
| rs2176040 | 2 | 227092802 | 0.353686 | A | G | 0.0149 | 0.0015 | -0.053 | 0.017 |
| rs1801282 | 3 | 12393125 | 0.119929 | G | C | 0.0203 | 0.0022 | -0.023 | 0.0247 |
| rs6792725 | 3 | 24520283 | 0.692002 | G | A | 0.0169 | 0.0016 | -0.0076 | 0.018 |
| rs784504 | 3 | 39195260 | 0.811877 | C | G | 0.0095 | 0.0018 | 0.0387 | 0.0213 |
| rs10461018 | 3 | 46995242 | 0.419984 | T | C | 0.0100 | 0.0014 | -0.0635 | 0.0166 |
| rs6772177 | 3 | 52497778 | 0.828337 | C | T | 0.0117 | 0.0019 | -0.0286 | 0.0267 |
| rs10511002 | 3 | 70631018 | 0.290003 | A | C | 0.0083 | 0.0016 | 0.0196 | 0.018 |
| rs687339 | 3 | 135932359 | 0.227041 | C | T | 0.0356 | 0.0017 | -0.0089 | 0.0196 |
| rs62271373 | 3 | 150066540 | 0.939897 | T | A | 0.0224 | 0.003 | 0.0972 | 0.044 |
| rs79287178 | 3 | 172294500 | 0.968996 | G | A | 0.0280 | 0.0043 | -0.0005 | 0.0587 |
| rs57158761 | 3 | 185371172 | 0.563749 | A | G | 0.0075 | 0.0014 | 0.015 | 0.0171 |
| rs34311866 | 4 | 951947 | 0.823863 | T | C | 0.0104 | 0.0019 | -0.0177 | 0.0226 |
| rs13108218 | 4 | 3443931 | 0.382898 | A | G | 0.0225 | 0.0015 | 0.0177 | 0.0184 |
| rs925098 | 4 | 17919811 | 0.264724 | G | A | 0.0098 | 0.0016 | -0.0353 | 0.0184 |
| rs28636815 | 4 | 77197397 | 0.377474 | G | A | 0.0110 | 0.0015 | 0.0183 | 0.0179 |
| rs13150068 | 4 | 88203828 | 0.564062 | A | G | 0.0172 | 0.0014 | -0.0082 | 0.0165 |
| rs138204164 | 4 | 120123417 | 0.874410 | C | G | 0.0114 | 0.0021 | 0.0223 | 0.0248 |
| rs28925904 | 4 | 144359490 | 0.974702 | C | T | 0.0283 | 0.0045 | -0.2548 | 0.4341 |
| rs10857228 | 4 | 148979700 | 0.256721 | T | C | 0.0086 | 0.0016 | 0.0184 | 0.0187 |
| rs28712547 | 4 | 157646955 | 0.320950 | G | A | 0.0096 | 0.0015 | -0.0179 | 0.0172 |
| rs78890745 | 4 | 159834474 | 0.108795 | A | G | 0.0218 | 0.0023 | 0.0627 | 0.0377 |
| rs11738093 | 5 | 53301425 | 0.748369 | A | G | 0.0119 | 0.0016 | -0.0029 | 0.0182 |
| rs40270 | 5 | 55804552 | 0.226690 | A | C | 0.0189 | 0.0017 | 0.0032 | 0.0199 |
| rs4976033 | 5 | 67714246 | 0.598947 | A | G | 0.0087 | 0.0015 | 0.0047 | 0.0202 |
| rs6860245 | 5 | 127367998 | 0.247090 | C | G | 0.0107 | 0.0017 | -0.0169 | 0.0192 |
| rs1650527 | 5 | 158022724 | 0.767899 | C | T | 0.0107 | 0.0017 | -0.0115 | 0.0194 |
| rs6879874 | 5 | 176730775 | 0.724183 | T | A | 0.0077 | 0.0016 | 0.0576 | 0.0185 |
| rs9379084 | 6 | 7231843 | 0.884204 | G | A | 0.0148 | 0.0023 | 0.0844 | 0.0298 |
| rs9366291 | 6 | 19381870 | 0.577052 | C | G | 0.0076 | 0.0014 | 0.0193 | 0.0181 |
| rs1634791 | 6 | 31276777 | 0.455909 | A | G | 0.0102 | 0.0015 | 0.2554 | 0.0183 |
| rs28360642 | 6 | 41667506 | 0.839480 | A | C | 0.0199 | 0.0019 | -0.0101 | 0.023 |
| rs11967262 | 6 | 43760327 | 0.511808 | C | G | 0.0094 | 0.0014 | 0.0223 | 0.0176 |
| rs1738386 | 6 | 151990235 | 0.379996 | C | T | 0.0086 | 0.0015 | -0.0107 | 0.0167 |
| rs555754 | 6 | 160769423 | 0.467759 | A | G | 0.0165 | 0.0014 | -0.0008 | 0.0157 |
| rs4563785 | 7 | 26349213 | 0.912931 | G | T | 0.0138 | 0.0025 | -0.0142 | 0.0291 |
| rs799157 | 7 | 73020301 | 0.043222 | T | C | 0.0207 | 0.0035 | 0.0154 | 0.0446 |
| rs1229498 | 7 | 81568750 | 0.275260 | T | G | 0.0112 | 0.0016 | 0.0084 | 0.0185 |
| rs10238028 | 7 | 99208899 | 0.067041 | G | A | 0.0188 | 0.0028 | 0.0279 | 0.0327 |
| rs6706 | 7 | 100471044 | 0.183551 | T | C | 0.0183 | 0.0018 | -0.0009 | 0.0206 |
| rs11556924 | 7 | 129663496 | 0.389097 | T | C | 0.0122 | 0.0015 | -0.0121 | 0.0212 |
| rs114949263 | 7 | 150498245 | 0.111159 | C | T | 0.0151 | 0.0023 | -0.0049 | 0.0272 |
| rs9987289 | 8 | 9183358 | 0.907871 | G | A | 0.0215 | 0.0025 | 0.0223 | 0.0291 |
| rs12543287 | 8 | 42334511 | 0.372017 | C | G | 0.0106 | 0.0015 | 0.0147 | 0.0184 |
| rs76767219 | 8 | 81426196 | 0.034451 | A | C | 0.0466 | 0.0039 | 0.0661 | 0.0613 |
| rs62515079 | 8 | 81442754 | 0.019907 | G | A | 0.0314 | 0.0052 | 0.0478 | 0.2778 |
| rs10095930 | 8 | 116974302 | 0.417115 | C | T | 0.0108 | 0.0014 | 0.0202 | 0.0166 |
| rs10108150 | 8 | 145687475 | 0.470017 | A | G | 0.0082 | 0.0014 | 0.0038 | 0.0169 |
| rs568656 | 9 | 4133874 | 0.351139 | C | A | 0.0111 | 0.0015 | 0.0013 | 0.0168 |
| rs1330307 | 9 | 4305064 | 0.514883 | A | C | 0.0088 | 0.0014 | 0.0031 | 0.0161 |
| rs10815276 | 9 | 5737692 | 0.456759 | G | A | 0.0089 | 0.0014 | -0.0079 | 0.0167 |
| rs820504 | 9 | 6668278 | 0.863965 | G | A | 0.0126 | 0.0021 | 0.0445 | 0.024 |
| rs696825 | 9 | 86583076 | 0.251847 | T | C | 0.0254 | 0.0016 | 0.0732 | 0.0185 |
| rs4876993 | 9 | 92281403 | 0.488052 | T | C | 0.0077 | 0.0014 | -0.0088 | 0.0165 |
| rs62580766 | 9 | 113034490 | 0.181214 | T | C | 0.0112 | 0.0018 | 0.0178 | 0.0213 |
| rs2986669 | 9 | 113187721 | 0.681436 | A | G | 0.0090 | 0.0015 | 0.015 | 0.0173 |
| rs4837794 | 9 | 123507855 | 0.331585 | T | C | 0.0119 | 0.0015 | 0.0139 | 0.0178 |
| rs9697210 | 9 | 131468740 | 0.853586 | G | A | 0.0161 | 0.002 | -0.0332 | 0.0443 |
| rs11791747 | 9 | 137106879 | 0.306547 | G | A | 0.0106 | 0.0016 | -0.003 | 0.018 |
| rs35233014 | 9 | 137268177 | 0.254515 | C | A | 0.0143 | 0.0016 | -0.0043 | 0.0192 |
| rs11103377 | 9 | 139097135 | 0.538173 | G | A | 0.0076 | 0.0014 | -0.0188 | 0.0169 |
| rs7475279 | 10 | 5252866 | 0.845559 | A | C | 0.0209 | 0.002 | 0.0105 | 0.0227 |
| rs1530439 | 10 | 63645959 | 0.308785 | T | G | 0.0123 | 0.0015 | 0.0203 | 0.018 |
| rs1782652 | 10 | 81074125 | 0.618815 | T | A | 0.0122 | 0.0015 | 0.0268 | 0.0187 |
| rs11186719 | 10 | 93631956 | 0.522156 | A | C | 0.0116 | 0.0014 | -0.0081 | 0.0163 |
| rs2068888 | 10 | 94839642 | 0.451225 | A | G | 0.0115 | 0.0014 | 0.0079 | 0.0167 |
| rs11188601 | 10 | 97856899 | 0.364454 | C | T | 0.0097 | 0.0015 | 0.0199 | 0.0168 |
| rs10883451 | 10 | 101924418 | 0.498806 | C | T | 0.0087 | 0.0014 | -0.0105 | 0.0163 |
| rs140312320 | 10 | 103992418 | 0.932876 | G | A | 0.0162 | 0.0028 | 0.0759 | 0.0351 |
| rs35198068 | 10 | 114754784 | 0.709536 | T | C | 0.0079 | 0.0016 | -0.0293 | 0.0182 |
| rs80235628 | 10 | 122859270 | 0.950127 | G | A | 0.0212 | 0.0033 | -0.0492 | 0.0374 |
| rs11601507 | 11 | 5701074 | 0.069169 | A | C | 0.0161 | 0.0028 | -0.0301 | 0.0409 |
| rs1037169 | 11 | 13361005 | 0.313024 | T | C | 0.0153 | 0.0015 | 0.004 | 0.0174 |
| rs174537 | 11 | 61552680 | 0.654276 | G | T | 0.0140 | 0.0015 | 0.0095 | 0.0172 |
| rs12797706 | 11 | 65561369 | 0.234579 | A | G | 0.0139 | 0.0017 | -0.0521 | 0.0197 |
| rs3018695 | 11 | 68911500 | 0.492988 | A | C | 0.0080 | 0.0014 | 0.0223 | 0.0163 |
| rs12804411 | 11 | 69284200 | 0.231234 | T | C | 0.0153 | 0.0017 | 0.0228 | 0.0218 |
| rs11021232 | 11 | 95320808 | 0.820213 | T | C | 0.0134 | 0.0019 | -0.0888 | 0.0219 |
| rs76895963 | 12 | 4384844 | 0.020964 | G | T | 0.0730 | 0.0054 | -0.0578 | 0.2166 |
| rs17887160 | 12 | 6877721 | 0.720025 | C | T | 0.0087 | 0.0016 | 0.0317 | 0.0196 |
| rs3782735 | 12 | 6885076 | 0.600654 | A | G | 0.0112 | 0.0015 | 0.0254 | 0.0172 |
| rs7298820 | 12 | 20581339 | 0.205464 | T | A | 0.0139 | 0.0018 | 0.0121 | 0.0207 |
| rs4149056 | 12 | 21331549 | 0.848805 | T | C | 0.0269 | 0.002 | -0.0196 | 0.0223 |
| rs11047237 | 12 | 24206326 | 0.965318 | A | T | 0.0297 | 0.0039 | 0.0089 | 0.0421 |
| rs75130744 | 12 | 25410741 | 0.928584 | G | C | 0.0261 | 0.0028 | 0.0537 | 0.0365 |
| rs4307773 | 12 | 51144432 | 0.418763 | T | C | 0.0133 | 0.0014 | -0.043 | 0.0165 |
| rs7484541 | 12 | 57714803 | 0.227751 | T | A | 0.0141 | 0.0017 | 0.0036 | 0.0204 |
| rs8756 | 12 | 66359752 | 0.485175 | C | A | 0.0088 | 0.0014 | 0.0058 | 0.0162 |
| rs3751129 | 12 | 102455729 | 0.216322 | A | G | 0.0100 | 0.0017 | 0.0192 | 0.0197 |
| rs183015141 | 12 | 112069836 | 0.973296 | G | A | 0.0288 | 0.0046 | 0.1766 | 0.1507 |
| rs7139079 | 12 | 121415293 | 0.592779 | A | G | 0.0125 | 0.0014 | -0.0115 | 0.0167 |
| rs12311848 | 12 | 124486851 | 0.332566 | G | A | 0.0109 | 0.0015 | 0.0094 | 0.0172 |
| rs17128091 | 14 | 23714682 | 0.255860 | C | G | 0.0123 | 0.0016 | -0.0189 | 0.0205 |
| rs11621792 | 14 | 24871926 | 0.547669 | C | T | 0.0248 | 0.0014 | -0.0267 | 0.0174 |
| rs2239222 | 14 | 73011885 | 0.349334 | G | A | 0.0088 | 0.0015 | 0.0099 | 0.0173 |
| rs13379043 | 14 | 74250126 | 0.277993 | C | T | 0.0100 | 0.0016 | -0.0065 | 0.0187 |
| rs1005421 | 14 | 89886940 | 0.583258 | C | T | 0.0087 | 0.0015 | -0.0166 | 0.0197 |
| rs28929474 | 14 | 94844947 | 0.020150 | T | C | 0.0627 | 0.0051 | 0.1007 | 0.0602 |
| rs17580 | 14 | 94847262 | 0.047981 | A | T | 0.0231 | 0.0033 | 0.0689 | 0.0644 |
| rs2498786 | 14 | 105262368 | 0.384603 | C | G | 0.0130 | 0.0015 | -0.0113 | 0.0193 |
| rs12593818 | 15 | 35153930 | 0.262761 | T | C | 0.0099 | 0.0016 | 0.004 | 0.0185 |
| rs11637595 | 15 | 40387728 | 0.723599 | C | T | 0.0092 | 0.0016 | -0.0005 | 0.0185 |
| rs139974673 | 15 | 44027885 | 0.974365 | T | C | 0.0481 | 0.0045 | 0.0485 | 0.1179 |
| rs79391862 | 15 | 53739426 | 0.985902 | A | C | 0.0738 | 0.0061 | -0.2422 | 0.211 |
| rs8027064 | 15 | 53741826 | 0.035042 | A | G | 0.0260 | 0.0039 | 0.0093 | 0.0456 |
| rs12906447 | 15 | 96224270 | 0.551672 | C | T | 0.0087 | 0.0014 | -0.0131 | 0.0163 |
| rs56332871 | 15 | 96714816 | 0.272252 | A | C | 0.0379 | 0.0016 | 0.0301 | 0.0188 |
| rs1684608 | 16 | 4676852 | 0.807368 | C | A | 0.0092 | 0.0018 | -0.0163 | 0.021 |
| rs72782727 | 16 | 11878033 | 0.753720 | G | T | 0.0093 | 0.0017 | 0.0326 | 0.0196 |
| rs4122352 | 16 | 15174571 | 0.295545 | A | G | 0.0110 | 0.0016 | -0.0018 | 0.0223 |
| rs2925979 | 16 | 81534790 | 0.699772 | C | T | 0.0134 | 0.0016 | 0.0153 | 0.018 |
| rs67890964 | 16 | 83979317 | 0.373584 | C | T | 0.0089 | 0.0015 | -0.0255 | 0.023 |
| rs11641834 | 16 | 88070573 | 0.570554 | C | T | 0.0112 | 0.0014 | -0.0169 | 0.0182 |
| rs11078597 | 17 | 1618363 | 0.185905 | C | T | 0.0167 | 0.0018 | 0.0468 | 0.024 |
| rs858519 | 17 | 7531965 | 0.556552 | C | T | 0.1001 | 0.0014 | -0.021 | 0.0178 |
| rs17669311 | 17 | 13837051 | 0.609988 | G | A | 0.0087 | 0.0014 | 0.0084 | 0.0168 |
| rs2525570 | 17 | 29681245 | 0.600891 | G | A | 0.0088 | 0.0014 | -0.0195 | 0.0166 |
| rs17616365 | 17 | 38256401 | 0.967911 | G | A | 0.0259 | 0.004 | -0.0676 | 0.0539 |
| rs4264433 | 17 | 45737275 | 0.514481 | T | A | 0.0186 | 0.0014 | 0.0997 | 0.0163 |
| rs140302625 | 17 | 47379867 | 0.086510 | T | G | 0.0669 | 0.0025 | 0.0252 | 0.0303 |
| rs8178824 | 17 | 64224775 | 0.970217 | C | T | 0.0417 | 0.0041 | -0.0571 | 0.1403 |
| rs11079685 | 17 | 65257494 | 0.521766 | A | G | 0.0077 | 0.0014 | -0.0032 | 0.017 |
| rs72844546 | 17 | 73149850 | 0.345886 | C | T | 0.0097 | 0.0015 | -0.0142 | 0.017 |
| rs78057960 | 17 | 73779075 | 0.293576 | T | C | 0.0090 | 0.0015 | 0.0549 | 0.0182 |
| rs10153315 | 17 | 79481772 | 0.580864 | T | C | 0.0092 | 0.0014 | 0.0119 | 0.0175 |
| rs4092465 | 18 | 55080437 | 0.649015 | G | A | 0.0108 | 0.0015 | -0.0199 | 0.0173 |
| rs12454712 | 18 | 60845884 | 0.376392 | C | T | 0.0079 | 0.0015 | 0.0006 | 0.0172 |
| rs11539938 | 19 | 3062857 | 0.421359 | C | T | 0.0088 | 0.0015 | 0.0094 | 0.0261 |
| rs60018147 | 19 | 3375572 | 0.119859 | G | A | 0.0135 | 0.0023 | -0.0319 | 0.0568 |
| rs8107967 | 19 | 7972615 | 0.567302 | G | A | 0.0100 | 0.0014 | -0.0186 | 0.0179 |
| rs7250869 | 19 | 33887405 | 0.690021 | C | T | 0.0100 | 0.0015 | -0.0359 | 0.0176 |
| rs2018519 | 19 | 35559787 | 0.181242 | C | T | 0.0195 | 0.0018 | 0.013 | 0.0218 |
| rs5117 | 19 | 45418790 | 0.235459 | C | T | 0.0124 | 0.0017 | -0.0309 | 0.0222 |
| rs5112 | 19 | 45430280 | 0.532859 | G | C | 0.0089 | 0.0015 | 0.0755 | 0.0328 |
| rs73036519 | 19 | 45748362 | 0.699571 | G | C | 0.0116 | 0.0016 | 0.0075 | 0.0186 |
| rs34255979 | 19 | 46384830 | 0.121040 | T | C | 0.0273 | 0.0022 | -0.0342 | 0.0267 |
| rs62128735 | 19 | 48113604 | 0.696749 | A | G | 0.0092 | 0.0016 | 0.0163 | 0.0201 |
| rs59774409 | 19 | 50016748 | 0.082186 | T | C | 0.0161 | 0.0026 | -0.0195 | 0.0338 |
| rs4077285 | 19 | 56599405 | 0.906230 | G | C | 0.0141 | 0.0025 | 0.0212 | 0.0413 |
| rs13042148 | 20 | 32298286 | 0.845319 | C | T | 0.0145 | 0.002 | 0.0504 | 0.0246 |
| rs6088776 | 20 | 33835773 | 0.860648 | T | C | 0.0122 | 0.0021 | -0.0062 | 0.0226 |
| rs2207132 | 20 | 39142516 | 0.966320 | G | A | 0.0227 | 0.0039 | -0.0517 | 0.2112 |
| rs6073431 | 20 | 43040569 | 0.531700 | T | C | 0.0166 | 0.0015 | 0.0355 | 0.0168 |
| rs4810580 | 20 | 45594295 | 0.782389 | T | G | 0.0100 | 0.0017 | -0.0576 | 0.0197 |
| rs16995626 | 20 | 49540925 | 0.072253 | C | T | 0.0170 | 0.0028 | -0.0255 | 0.032 |
| rs1033667 | 22 | 29130300 | 0.299679 | T | C | 0.0109 | 0.0016 | 0.0226 | 0.018 |
| rs5753111 | 22 | 30779211 | 0.290673 | T | C | 0.0139 | 0.0016 | 0.0233 | 0.0181 |
| rs3747207 | 22 | 44324855 | 0.214383 | A | G | 0.0187 | 0.0017 | 0.0135 | 0.0195 |
| SHBG: sex hormone-binding globulin; SNP: single nucleotide polymorphism; Chr: chromosome; A1:effect allele; A2:other allele. | | | | | | | | | |

| **Table S3. The characteristic of SHBGadjBMI-associated index SNPs and their effect sizes with exposure and outcome.** | | | | | | | | | |
| --- | --- | --- | --- | --- | --- | --- | --- | --- | --- |
| **SNP** | **Chr** | **Position** | **Allele frequency** | **A1** | **A2** | **Exposure** | | **Outcome** | |
|  |  |  |  |  |  | **beta** | **se** | **beta** | **se** |
| rs198358 | 1 | 11904076 | 0.248320 | C | T | 0.0085 | 0.0014 | 0.0466 | 0.0189 |
| rs114165349 | 1 | 27021913 | 0.976296 | G | C | 0.0740 | 0.0041 | -0.0508 | 0.0592 |
| rs74090351 | 1 | 61705898 | 0.068767 | A | G | 0.0194 | 0.0024 | 0.0073 | 0.0337 |
| rs469721 | 1 | 91530001 | 0.803120 | C | T | 0.0107 | 0.0015 | -0.0364 | 0.0219 |
| rs1730862 | 1 | 107614003 | 0.341827 | G | A | 0.0227 | 0.0013 | 0.0225 | 0.0175 |
| rs267733 | 1 | 150958836 | 0.838969 | A | G | 0.0144 | 0.0017 | 0.0278 | 0.0227 |
| rs9426829 | 1 | 154592201 | 0.480729 | C | T | 0.0139 | 0.0012 | 0.0027 | 0.0162 |
| rs10489206 | 1 | 168041232 | 0.779271 | C | T | 0.0079 | 0.0015 | 0.0116 | 0.0203 |
| rs2064074 | 1 | 171061990 | 0.528459 | A | G | 0.0064 | 0.0012 | 0.0163 | 0.0205 |
| rs12138803 | 1 | 172348823 | 0.730533 | C | T | 0.0086 | 0.0014 | -0.0305 | 0.0186 |
| rs34331968 | 1 | 196659753 | 0.533525 | T | C | 0.0111 | 0.0012 | -0.0076 | 0.0164 |
| rs17583875 | 1 | 197924770 | 0.020867 | A | G | 0.0290 | 0.0043 | 0.0386 | 0.0563 |
| rs1418652 | 1 | 205646458 | 0.386157 | C | T | 0.0071 | 0.0013 | 0.0210 | 0.0169 |
| rs1223796 | 1 | 214323347 | 0.163998 | G | C | 0.0137 | 0.0017 | 0.0204 | 0.0223 |
| rs3001032 | 1 | 219727779 | 0.319697 | C | T | 0.0147 | 0.0013 | 0.0121 | 0.0177 |
| rs61830291 | 1 | 221001142 | 0.096820 | C | A | 0.0115 | 0.0021 | 0.0697 | 0.0348 |
| rs3768420 | 1 | 227173537 | 0.785749 | C | T | 0.0085 | 0.0015 | -0.0018 | 0.0195 |
| rs7567544 | 2 | 20392802 | 0.454669 | C | G | 0.0076 | 0.0012 | 0.0124 | 0.0168 |
| rs1260326 | 2 | 27730940 | 0.607415 | C | T | 0.0352 | 0.0013 | 0.0092 | 0.0166 |
| rs6736913 | 2 | 42510018 | 0.020645 | A | G | 0.0368 | 0.0043 | -0.2761 | 0.3082 |
| rs11690748 | 2 | 48584575 | 0.622710 | C | G | 0.0079 | 0.0013 | -0.0183 | 0.0168 |
| rs921153 | 2 | 61563408 | 0.158984 | A | G | 0.0094 | 0.0017 | -0.0175 | 0.0226 |
| rs6546096 | 2 | 64906295 | 0.262505 | A | G | 0.0252 | 0.0014 | -0.0151 | 0.0184 |
| rs12624244 | 2 | 70417138 | 0.935671 | A | G | 0.0218 | 0.0025 | -0.0219 | 0.0321 |
| rs13394092 | 2 | 85815954 | 0.169814 | C | T | 0.0085 | 0.0016 | -0.0123 | 0.0220 |
| rs72836346 | 2 | 111876613 | 0.078561 | C | G | 0.0155 | 0.0023 | -0.0685 | 0.0305 |
| rs12613243 | 2 | 111897506 | 0.936726 | T | C | 0.0188 | 0.0025 | -0.0402 | 0.0337 |
| rs13018007 | 2 | 114971913 | 0.082429 | G | A | 0.0127 | 0.0023 | -0.0042 | 0.0295 |
| rs11688682 | 2 | 121347612 | 0.270439 | C | G | 0.0098 | 0.0014 | 0.0394 | 0.0248 |
| rs1128249 | 2 | 165528624 | 0.392363 | T | G | 0.0217 | 0.0013 | -0.0095 | 0.0167 |
| rs2364717 | 2 | 178101235 | 0.538422 | T | C | 0.0076 | 0.0012 | 0.0296 | 0.0165 |
| rs1047891 | 2 | 211540507 | 0.315407 | A | C | 0.0179 | 0.0013 | 0.0093 | 0.0193 |
| rs10189479 | 2 | 219287276 | 0.434028 | A | C | 0.0093 | 0.0012 | 0.0203 | 0.0165 |
| rs2176040 | 2 | 227092802 | 0.353686 | A | G | 0.0151 | 0.0013 | -0.0530 | 0.0170 |
| rs2924808 | 2 | 234329816 | 0.345470 | C | G | 0.0070 | 0.0013 | 0.0336 | 0.0172 |
| rs62186584 | 2 | 241853621 | 0.743514 | C | T | 0.0080 | 0.0014 | -0.0040 | 0.0204 |
| rs1801282 | 3 | 12393125 | 0.119929 | G | C | 0.0242 | 0.0019 | -0.0230 | 0.0247 |
| rs6792725 | 3 | 24520283 | 0.692002 | G | A | 0.0177 | 0.0014 | -0.0076 | 0.0180 |
| rs784504 | 3 | 39195260 | 0.811877 | C | G | 0.0105 | 0.0016 | 0.0387 | 0.0213 |
| rs10461018 | 3 | 46995242 | 0.419984 | T | C | 0.0106 | 0.0013 | -0.0635 | 0.0166 |
| rs6772177 | 3 | 52497778 | 0.828337 | C | T | 0.0107 | 0.0016 | -0.0286 | 0.0267 |
| rs11130982 | 3 | 64728312 | 0.292996 | T | G | 0.0087 | 0.0014 | 0.0004 | 0.0219 |
| rs10511002 | 3 | 70631018 | 0.290003 | A | C | 0.0081 | 0.0014 | 0.0196 | 0.0180 |
| rs4530527 | 3 | 86800085 | 0.361086 | C | A | 0.0079 | 0.0013 | 0.0146 | 0.0170 |
| rs17202341 | 3 | 105452593 | 0.348154 | G | A | 0.0075 | 0.0013 | 0.0575 | 0.0179 |
| rs11720108 | 3 | 123069058 | 0.248736 | T | C | 0.0081 | 0.0014 | -0.0016 | 0.0191 |
| rs6803518 | 3 | 129735650 | 0.754350 | T | C | 0.0071 | 0.0015 | 0.0010 | 0.0222 |
| rs687339 | 3 | 135932359 | 0.227041 | C | T | 0.0308 | 0.0015 | -0.0089 | 0.0196 |
| rs9872754 | 3 | 138117985 | 0.839833 | C | T | 0.0103 | 0.0017 | -0.0716 | 0.0223 |
| rs9834503 | 3 | 149994882 | 0.539659 | A | C | 0.0080 | 0.0013 | -0.0447 | 0.0182 |
| rs62271373 | 3 | 150066540 | 0.939897 | T | A | 0.0260 | 0.0026 | 0.0972 | 0.0440 |
| rs1126161 | 3 | 172228827 | 0.673134 | G | A | 0.0076 | 0.0013 | 0.0144 | 0.0174 |
| rs79287178 | 3 | 172294500 | 0.968996 | G | A | 0.0317 | 0.0037 | -0.0005 | 0.0587 |
| rs59837038 | 3 | 172317573 | 0.269318 | T | C | 0.0093 | 0.0014 | 0.0177 | 0.0207 |
| rs3749228 | 3 | 184099342 | 0.062171 | C | G | 0.0161 | 0.0026 | 0.0208 | 0.0368 |
| rs57158761 | 3 | 185371172 | 0.563749 | A | G | 0.0098 | 0.0012 | 0.0150 | 0.0171 |
| rs34311866 | 4 | 951947 | 0.823863 | T | C | 0.0112 | 0.0016 | -0.0177 | 0.0226 |
| rs13108218 | 4 | 3443931 | 0.382898 | A | G | 0.0235 | 0.0013 | 0.0177 | 0.0184 |
| rs4450871 | 4 | 4990298 | 0.442403 | G | A | 0.0092 | 0.0012 | -0.0026 | 0.0276 |
| rs925098 | 4 | 17919811 | 0.264724 | G | A | 0.0088 | 0.0014 | -0.0353 | 0.0184 |
| rs2970871 | 4 | 23890582 | 0.441213 | T | C | 0.0073 | 0.0012 | -0.0115 | 0.0172 |
| rs6531735 | 4 | 39686332 | 0.492492 | G | A | 0.0064 | 0.0012 | 0.0224 | 0.0165 |
| rs62303689 | 4 | 56295873 | 0.874913 | C | A | 0.0120 | 0.0019 | -0.0173 | 0.0237 |
| rs7696472 | 4 | 69538180 | 0.474738 | A | G | 0.0066 | 0.0012 | -0.0186 | 0.0211 |
| rs28636815 | 4 | 77197397 | 0.377474 | G | A | 0.0117 | 0.0013 | 0.0183 | 0.0179 |
| rs13150068 | 4 | 88203828 | 0.564062 | A | G | 0.0167 | 0.0012 | -0.0082 | 0.0165 |
| rs6831257 | 4 | 100018260 | 0.340111 | G | A | 0.0080 | 0.0013 | -0.0098 | 0.0170 |
| rs138204164 | 4 | 120123417 | 0.874410 | C | G | 0.0116 | 0.0019 | 0.0223 | 0.0248 |
| rs1433210 | 4 | 124766956 | 0.245263 | C | A | 0.0101 | 0.0014 | -0.0003 | 0.0172 |
| rs28925904 | 4 | 144359490 | 0.974702 | C | T | 0.0227 | 0.0039 | -0.2548 | 0.4341 |
| rs10857228 | 4 | 148979700 | 0.256721 | T | C | 0.0097 | 0.0014 | 0.0184 | 0.0187 |
| rs41280463 | 4 | 154191226 | 0.832659 | G | A | 0.0103 | 0.0016 | 0.0185 | 0.0234 |
| rs28712547 | 4 | 157646955 | 0.320950 | G | A | 0.0096 | 0.0013 | -0.0179 | 0.0172 |
| rs78890745 | 4 | 159834474 | 0.108795 | A | G | 0.0202 | 0.0020 | 0.0627 | 0.0377 |
| rs11738093 | 5 | 53301425 | 0.748369 | A | G | 0.0139 | 0.0014 | -0.0029 | 0.0182 |
| rs40270 | 5 | 55804552 | 0.226690 | A | C | 0.0177 | 0.0015 | 0.0032 | 0.0199 |
| rs10939934 | 5 | 61531501 | 0.422369 | A | G | 0.0075 | 0.0013 | -0.0010 | 0.0175 |
| rs4976033 | 5 | 67714246 | 0.598947 | A | G | 0.0106 | 0.0013 | 0.0047 | 0.0202 |
| rs34651 | 5 | 72144005 | 0.917965 | T | C | 0.0114 | 0.0023 | -0.0915 | 0.0512 |
| rs6860245 | 5 | 127367998 | 0.247090 | C | G | 0.0107 | 0.0014 | -0.0169 | 0.0192 |
| rs2057655 | 5 | 131807624 | 0.186744 | A | G | 0.0109 | 0.0016 | -0.0239 | 0.0207 |
| rs1650527 | 5 | 158022724 | 0.767899 | C | T | 0.0137 | 0.0015 | -0.0115 | 0.0194 |
| rs6879874 | 5 | 176730775 | 0.724183 | T | A | 0.0075 | 0.0014 | 0.0576 | 0.0185 |
| rs72648854 | 5 | 180222312 | 0.051255 | C | T | 0.0190 | 0.0032 | 0.0245 | 0.0292 |
| rs9379084 | 6 | 7231843 | 0.884204 | G | A | 0.0127 | 0.0020 | 0.0844 | 0.0298 |
| rs2299055 | 6 | 15398331 | 0.117375 | A | G | 0.0107 | 0.0019 | 0.0282 | 0.0254 |
| rs9366291 | 6 | 19381870 | 0.577052 | C | G | 0.0063 | 0.0013 | 0.0193 | 0.0181 |
| rs1634791 | 6 | 31276777 | 0.455909 | A | G | 0.0118 | 0.0013 | 0.2554 | 0.0183 |
| rs28360642 | 6 | 41667506 | 0.839480 | A | C | 0.0205 | 0.0017 | -0.0101 | 0.0230 |
| rs11967262 | 6 | 43760327 | 0.511808 | C | G | 0.0110 | 0.0012 | 0.0223 | 0.0176 |
| rs13200245 | 6 | 95949746 | 0.840884 | A | G | 0.0093 | 0.0017 | 0.0311 | 0.0232 |
| rs150115323 | 6 | 117506408 | 0.372036 | G | C | 0.0064 | 0.0013 | 0.0082 | 0.0176 |
| rs58321169 | 6 | 126868567 | 0.732326 | C | T | 0.0101 | 0.0014 | 0.0158 | 0.0244 |
| rs1738386 | 6 | 151990235 | 0.379996 | C | T | 0.0081 | 0.0013 | -0.0107 | 0.0167 |
| rs555754 | 6 | 160769423 | 0.467759 | A | G | 0.0183 | 0.0012 | -0.0008 | 0.0157 |
| rs4709746 | 6 | 164133001 | 0.134119 | T | C | 0.0118 | 0.0018 | -0.0049 | 0.0246 |
| rs73670309 | 7 | 1065947 | 0.893632 | C | A | 0.0118 | 0.0020 | -0.0013 | 0.0382 |
| rs2246223 | 7 | 6701189 | 0.555122 | T | C | 0.0077 | 0.0012 | 0.0231 | 0.0171 |
| rs10486782 | 7 | 15884421 | 0.761060 | A | G | 0.0083 | 0.0015 | -0.0024 | 0.0213 |
| rs2723555 | 7 | 17863633 | 0.488453 | T | A | 0.0064 | 0.0012 | -0.0078 | 0.0164 |
| rs28459049 | 7 | 21567331 | 0.787203 | C | T | 0.0090 | 0.0015 | 0.0005 | 0.0211 |
| rs4563785 | 7 | 26349213 | 0.912931 | G | T | 0.0145 | 0.0022 | -0.0142 | 0.0291 |
| rs13237750 | 7 | 46456878 | 0.951761 | C | T | 0.0163 | 0.0029 | -0.0369 | 0.0438 |
| rs17492269 | 7 | 70047405 | 0.823585 | G | A | 0.0098 | 0.0016 | 0.0402 | 0.0219 |
| rs799157 | 7 | 73020301 | 0.043222 | T | C | 0.0170 | 0.0030 | 0.0154 | 0.0446 |
| rs848476 | 7 | 77541673 | 0.292159 | G | A | 0.0104 | 0.0014 | 0.0177 | 0.0182 |
| rs1229498 | 7 | 81568750 | 0.275260 | T | G | 0.0113 | 0.0014 | 0.0084 | 0.0185 |
| rs10238028 | 7 | 99208899 | 0.067041 | G | A | 0.0142 | 0.0025 | 0.0279 | 0.0327 |
| rs6706 | 7 | 100471044 | 0.183551 | T | C | 0.0172 | 0.0016 | -0.0009 | 0.0206 |
| rs11556924 | 7 | 129663496 | 0.389097 | T | C | 0.0113 | 0.0013 | -0.0121 | 0.0212 |
| rs114949263 | 7 | 150498245 | 0.111159 | C | T | 0.0139 | 0.0020 | -0.0049 | 0.0272 |
| rs9987289 | 8 | 9183358 | 0.907871 | G | A | 0.0216 | 0.0021 | 0.0223 | 0.0291 |
| rs62486442 | 8 | 12623463 | 0.665337 | G | A | 0.0076 | 0.0013 | -0.0070 | 0.0188 |
| rs9644032 | 8 | 23414822 | 0.368235 | T | G | 0.0082 | 0.0013 | 0.0152 | 0.0169 |
| rs10095380 | 8 | 36853217 | 0.834373 | G | C | 0.0119 | 0.0017 | -0.0287 | 0.0212 |
| rs12543287 | 8 | 42334511 | 0.372017 | C | G | 0.0105 | 0.0013 | 0.0147 | 0.0184 |
| rs76767219 | 8 | 81426196 | 0.034451 | A | C | 0.0484 | 0.0034 | 0.0661 | 0.0613 |
| rs62515079 | 8 | 81442754 | 0.019907 | G | A | 0.0321 | 0.0045 | 0.0478 | 0.2778 |
| rs10095930 | 8 | 116974302 | 0.417115 | C | T | 0.0097 | 0.0013 | 0.0202 | 0.0166 |
| rs11774700 | 8 | 118220270 | 0.308787 | C | T | 0.0075 | 0.0013 | -0.0093 | 0.0205 |
| rs2980858 | 8 | 126501177 | 0.696302 | C | T | 0.0099 | 0.0013 | -0.0049 | 0.0177 |
| rs4871015 | 8 | 128314516 | 0.581492 | A | G | 0.0079 | 0.0013 | 0.0044 | 0.0176 |
| rs11780978 | 8 | 145034852 | 0.403073 | A | G | 0.0088 | 0.0013 | 0.0611 | 0.0167 |
| rs10108150 | 8 | 145687475 | 0.470017 | A | G | 0.0087 | 0.0012 | 0.0038 | 0.0169 |
| rs868655 | 9 | 1037118 | 0.691635 | C | A | 0.0089 | 0.0013 | -0.0077 | 0.0178 |
| rs568656 | 9 | 4133874 | 0.351139 | C | A | 0.0116 | 0.0013 | 0.0013 | 0.0168 |
| rs1330307 | 9 | 4305064 | 0.514883 | A | C | 0.0079 | 0.0012 | 0.0031 | 0.0161 |
| rs10815276 | 9 | 5737692 | 0.456759 | G | A | 0.0077 | 0.0012 | -0.0079 | 0.0167 |
| rs820504 | 9 | 6668278 | 0.863965 | G | A | 0.0137 | 0.0018 | 0.0445 | 0.0240 |
| rs10961205 | 9 | 13722479 | 0.582604 | A | G | 0.0071 | 0.0013 | 0.0039 | 0.0166 |
| rs696825 | 9 | 86583076 | 0.251847 | T | C | 0.0241 | 0.0014 | 0.0732 | 0.0185 |
| rs4876993 | 9 | 92281403 | 0.488052 | T | C | 0.0069 | 0.0012 | -0.0088 | 0.0165 |
| rs1962883 | 9 | 107722705 | 0.529820 | C | T | 0.0080 | 0.0013 | -0.0173 | 0.0188 |
| rs62580766 | 9 | 113034490 | 0.181214 | T | C | 0.0114 | 0.0016 | 0.0178 | 0.0213 |
| rs2986669 | 9 | 113187721 | 0.681436 | A | G | 0.0091 | 0.0013 | 0.0150 | 0.0173 |
| rs112332688 | 9 | 119083371 | 0.767684 | A | G | 0.0078 | 0.0015 | -0.0350 | 0.0197 |
| rs4837794 | 9 | 123507855 | 0.331585 | T | C | 0.0104 | 0.0013 | 0.0139 | 0.0178 |
| rs9697210 | 9 | 131468740 | 0.853586 | G | A | 0.0155 | 0.0018 | -0.0332 | 0.0443 |
| rs143378550 | 9 | 133069741 | 0.025498 | A | C | 0.0191 | 0.0043 | -0.1304 | 0.0616 |
| rs8176741 | 9 | 136131461 | 0.938152 | G | A | 0.0163 | 0.0026 | 0.0370 | 0.0307 |
| rs11791747 | 9 | 137106879 | 0.306547 | G | A | 0.0099 | 0.0014 | -0.0030 | 0.0180 |
| rs35233014 | 9 | 137268177 | 0.254515 | C | A | 0.0153 | 0.0014 | -0.0043 | 0.0192 |
| rs11103377 | 9 | 139097135 | 0.538173 | G | A | 0.0072 | 0.0012 | -0.0188 | 0.0169 |
| rs7475279 | 10 | 5252866 | 0.845559 | A | C | 0.0183 | 0.0017 | 0.0105 | 0.0227 |
| rs899865 | 10 | 36473044 | 0.599730 | T | C | 0.0069 | 0.0013 | 0.0073 | 0.0172 |
| rs1530439 | 10 | 63645959 | 0.308785 | T | G | 0.0115 | 0.0013 | 0.0203 | 0.0180 |
| rs2915023 | 10 | 77282899 | 0.902979 | G | A | 0.0108 | 0.0021 | -0.0044 | 0.0284 |
| rs1782652 | 10 | 81074125 | 0.618815 | T | A | 0.0136 | 0.0013 | 0.0268 | 0.0187 |
| rs11186719 | 10 | 93631956 | 0.522156 | A | C | 0.0118 | 0.0012 | -0.0081 | 0.0163 |
| rs2068888 | 10 | 94839642 | 0.451225 | A | G | 0.0131 | 0.0012 | 0.0079 | 0.0167 |
| rs11188601 | 10 | 97856899 | 0.364454 | C | T | 0.0097 | 0.0013 | 0.0199 | 0.0168 |
| rs10883451 | 10 | 101924418 | 0.498806 | C | T | 0.0088 | 0.0012 | -0.0105 | 0.0163 |
| rs140312320 | 10 | 103992418 | 0.932876 | G | A | 0.0167 | 0.0025 | 0.0759 | 0.0351 |
| rs35198068 | 10 | 114754784 | 0.709536 | T | C | 0.0112 | 0.0014 | -0.0293 | 0.0182 |
| rs80235628 | 10 | 122859270 | 0.950127 | G | A | 0.0247 | 0.0028 | -0.0492 | 0.0374 |
| rs11601507 | 11 | 5701074 | 0.069169 | A | C | 0.0183 | 0.0024 | -0.0301 | 0.0409 |
| rs1037169 | 11 | 13361005 | 0.313024 | T | C | 0.0130 | 0.0013 | 0.0040 | 0.0174 |
| rs76491020 | 11 | 32673898 | 0.093789 | C | G | 0.0123 | 0.0022 | 0.0413 | 0.0310 |
| rs185044544 | 11 | 58867435 | 0.974928 | T | G | 0.0219 | 0.0040 | 0.0831 | 0.0636 |
| rs174537 | 11 | 61552680 | 0.654276 | G | T | 0.0123 | 0.0013 | 0.0095 | 0.0172 |
| rs12797706 | 11 | 65561369 | 0.234579 | A | G | 0.0129 | 0.0015 | -0.0521 | 0.0197 |
| rs3018695 | 11 | 68911500 | 0.492988 | A | C | 0.0087 | 0.0012 | 0.0223 | 0.0163 |
| rs12280075 | 11 | 69170389 | 0.319901 | G | T | 0.0084 | 0.0013 | 0.0278 | 0.0181 |
| rs12804411 | 11 | 69284200 | 0.231234 | T | C | 0.0140 | 0.0015 | 0.0228 | 0.0218 |
| rs11021232 | 11 | 95320808 | 0.820213 | T | C | 0.0135 | 0.0016 | -0.0888 | 0.0219 |
| rs12787996 | 11 | 102057068 | 0.661075 | C | A | 0.0086 | 0.0013 | -0.0325 | 0.0172 |
| rs10893876 | 11 | 128353007 | 0.766412 | C | T | 0.0080 | 0.0015 | 0.0706 | 0.0200 |
| rs740893 | 12 | 676209 | 0.213982 | G | C | 0.0094 | 0.0015 | -0.0165 | 0.0202 |
| rs10774095 | 12 | 3125648 | 0.806533 | A | G | 0.0086 | 0.0016 | -0.0196 | 0.0222 |
| rs76895963 | 12 | 4384844 | 0.020964 | G | T | 0.0754 | 0.0047 | -0.0578 | 0.2166 |
| rs17887160 | 12 | 6877721 | 0.720025 | C | T | 0.0090 | 0.0014 | 0.0317 | 0.0196 |
| rs3782735 | 12 | 6885076 | 0.600654 | A | G | 0.0101 | 0.0013 | 0.0254 | 0.0172 |
| rs7298820 | 12 | 20581339 | 0.205464 | T | A | 0.0150 | 0.0015 | 0.0121 | 0.0207 |
| rs4149056 | 12 | 21331549 | 0.848805 | T | C | 0.0299 | 0.0017 | -0.0196 | 0.0223 |
| rs11047237 | 12 | 24206326 | 0.965318 | A | T | 0.0275 | 0.0034 | 0.0089 | 0.0421 |
| rs75130744 | 12 | 25410741 | 0.928584 | G | C | 0.0257 | 0.0024 | 0.0537 | 0.0365 |
| rs1872992 | 12 | 26457190 | 0.756315 | A | G | 0.0086 | 0.0014 | -0.0002 | 0.0175 |
| rs10880868 | 12 | 46322449 | 0.788982 | C | T | 0.0080 | 0.0015 | 0.0530 | 0.0218 |
| rs4307773 | 12 | 51144432 | 0.418763 | T | C | 0.0142 | 0.0012 | -0.0430 | 0.0165 |
| rs7484541 | 12 | 57714803 | 0.227751 | T | A | 0.0159 | 0.0015 | 0.0036 | 0.0204 |
| rs8756 | 12 | 66359752 | 0.485175 | C | A | 0.0085 | 0.0012 | 0.0058 | 0.0162 |
| rs2438109 | 12 | 67653617 | 0.656713 | C | T | 0.0065 | 0.0013 | 0.0178 | 0.0172 |
| rs3751129 | 12 | 102455729 | 0.216322 | A | G | 0.0108 | 0.0015 | 0.0192 | 0.0197 |
| rs183015141 | 12 | 112069836 | 0.973296 | G | A | 0.0244 | 0.0040 | 0.1766 | 0.1507 |
| rs7139079 | 12 | 121415293 | 0.592779 | A | G | 0.0129 | 0.0013 | -0.0115 | 0.0167 |
| rs12311848 | 12 | 124486851 | 0.332566 | G | A | 0.0141 | 0.0013 | 0.0094 | 0.0172 |
| rs12864658 | 13 | 50666074 | 0.050964 | T | C | 0.0187 | 0.0028 | -0.0403 | 0.0519 |
| rs9556403 | 13 | 95236825 | 0.350483 | G | A | 0.0072 | 0.0013 | -0.0042 | 0.0168 |
| rs7321688 | 13 | 115000365 | 0.766545 | C | A | 0.0086 | 0.0015 | -0.0190 | 0.0192 |
| rs17128091 | 14 | 23714682 | 0.255860 | C | G | 0.0126 | 0.0014 | -0.0189 | 0.0205 |
| rs11621792 | 14 | 24871926 | 0.547669 | C | T | 0.0261 | 0.0013 | -0.0267 | 0.0174 |
| rs2239222 | 14 | 73011885 | 0.349334 | G | A | 0.0100 | 0.0013 | 0.0099 | 0.0173 |
| rs13379043 | 14 | 74250126 | 0.277993 | C | T | 0.0113 | 0.0014 | -0.0065 | 0.0187 |
| rs1005421 | 14 | 89886940 | 0.583258 | C | T | 0.0081 | 0.0013 | -0.0166 | 0.0197 |
| rs28929474 | 14 | 94844947 | 0.020150 | T | C | 0.0609 | 0.0044 | 0.1007 | 0.0602 |
| rs17580 | 14 | 94847262 | 0.047981 | A | T | 0.0240 | 0.0029 | 0.0689 | 0.0644 |
| rs2498786 | 14 | 105262368 | 0.384603 | C | G | 0.0110 | 0.0013 | -0.0113 | 0.0193 |
| rs12593818 | 15 | 35153930 | 0.262761 | T | C | 0.0088 | 0.0014 | 0.0040 | 0.0185 |
| rs275177 | 15 | 39449003 | 0.149747 | C | T | 0.0100 | 0.0018 | -0.0221 | 0.0228 |
| rs11637595 | 15 | 40387728 | 0.723599 | C | T | 0.0120 | 0.0014 | -0.0005 | 0.0185 |
| rs2009310 | 15 | 41965591 | 0.496397 | T | G | 0.0083 | 0.0012 | -0.0165 | 0.0162 |
| rs139974673 | 15 | 44027885 | 0.974365 | T | C | 0.0537 | 0.0039 | 0.0485 | 0.1179 |
| rs79391862 | 15 | 53739426 | 0.985902 | A | C | 0.0731 | 0.0053 | -0.2422 | 0.2110 |
| rs79237700 | 15 | 53741612 | 0.961839 | T | C | 0.0203 | 0.0032 | 0.0228 | 0.1274 |
| rs8027064 | 15 | 53741826 | 0.035042 | A | G | 0.0266 | 0.0034 | 0.0093 | 0.0456 |
| rs12438742 | 15 | 61947280 | 0.570004 | G | C | 0.0069 | 0.0013 | 0.0100 | 0.0167 |
| rs12906447 | 15 | 96224270 | 0.551672 | C | T | 0.0090 | 0.0013 | -0.0131 | 0.0163 |
| rs56332871 | 15 | 96714816 | 0.272252 | A | C | 0.0387 | 0.0014 | 0.0301 | 0.0188 |
| rs1684608 | 16 | 4676852 | 0.807368 | C | A | 0.0090 | 0.0016 | -0.0163 | 0.0210 |
| rs72782727 | 16 | 11878033 | 0.753720 | G | T | 0.0087 | 0.0014 | 0.0326 | 0.0196 |
| rs4122352 | 16 | 15174571 | 0.295545 | A | G | 0.0108 | 0.0014 | -0.0018 | 0.0223 |
| rs2925979 | 16 | 81534790 | 0.699772 | C | T | 0.0135 | 0.0014 | 0.0153 | 0.0180 |
| rs67890964 | 16 | 83979317 | 0.373584 | C | T | 0.0105 | 0.0013 | -0.0255 | 0.0230 |
| rs11641834 | 16 | 88070573 | 0.570554 | C | T | 0.0104 | 0.0013 | -0.0169 | 0.0182 |
| rs67651018 | 16 | 88527222 | 0.306898 | A | G | 0.0074 | 0.0013 | -0.0367 | 0.0188 |
| rs11078597 | 17 | 1618363 | 0.185905 | C | T | 0.0156 | 0.0015 | 0.0468 | 0.0240 |
| rs858519 | 17 | 7531965 | 0.556552 | C | T | 0.0988 | 0.0012 | -0.0210 | 0.0178 |
| rs17669311 | 17 | 13837051 | 0.609988 | G | A | 0.0094 | 0.0012 | 0.0084 | 0.0168 |
| rs2525570 | 17 | 29681245 | 0.600891 | G | A | 0.0072 | 0.0012 | -0.0195 | 0.0166 |
| rs17616365 | 17 | 38256401 | 0.967911 | G | A | 0.0280 | 0.0034 | -0.0676 | 0.0539 |
| rs4264433 | 17 | 45737275 | 0.514481 | T | A | 0.0195 | 0.0012 | 0.0997 | 0.0163 |
| rs140302625 | 17 | 47379867 | 0.086510 | T | G | 0.0622 | 0.0021 | 0.0252 | 0.0303 |
| rs8178824 | 17 | 64224775 | 0.970217 | C | T | 0.0427 | 0.0035 | -0.0571 | 0.1403 |
| rs11079685 | 17 | 65257494 | 0.521766 | A | G | 0.0080 | 0.0012 | -0.0032 | 0.0170 |
| rs740516 | 17 | 67082962 | 0.849078 | C | G | 0.0094 | 0.0017 | 0.0177 | 0.0280 |
| rs72844546 | 17 | 73149850 | 0.345886 | C | T | 0.0103 | 0.0013 | -0.0142 | 0.0170 |
| rs78057960 | 17 | 73779075 | 0.293576 | T | C | 0.0113 | 0.0013 | 0.0549 | 0.0182 |
| rs10153315 | 17 | 79481772 | 0.580864 | T | C | 0.0093 | 0.0012 | 0.0119 | 0.0175 |
| rs11664106 | 18 | 2846812 | 0.372641 | T | A | 0.0084 | 0.0013 | 0.0115 | 0.0189 |
| rs4092465 | 18 | 55080437 | 0.649015 | G | A | 0.0118 | 0.0013 | -0.0199 | 0.0173 |
| rs12454712 | 18 | 60845884 | 0.376392 | C | T | 0.0104 | 0.0013 | 0.0006 | 0.0172 |
| rs4327143 | 18 | 71925113 | 0.714237 | A | G | 0.0087 | 0.0014 | -0.0204 | 0.0183 |
| rs892225 | 19 | 1152656 | 0.380269 | G | A | 0.0085 | 0.0013 | -0.0233 | 0.0246 |
| rs11539938 | 19 | 3062857 | 0.421359 | C | T | 0.0088 | 0.0013 | 0.0094 | 0.0261 |
| rs60018147 | 19 | 3375572 | 0.119859 | G | A | 0.0155 | 0.0020 | -0.0319 | 0.0568 |
| rs2288926 | 19 | 4498245 | 0.310853 | A | G | 0.0069 | 0.0013 | 0.0374 | 0.0178 |
| rs8107967 | 19 | 7972615 | 0.567302 | G | A | 0.0086 | 0.0012 | -0.0186 | 0.0179 |
| rs4804669 | 19 | 12502457 | 0.217168 | A | G | 0.0092 | 0.0015 | -0.0536 | 0.0227 |
| rs7252372 | 19 | 14172896 | 0.556970 | G | C | 0.0071 | 0.0013 | -0.0108 | 0.0233 |
| rs58489806 | 19 | 19456917 | 0.912819 | C | T | 0.0121 | 0.0022 | 0.0373 | 0.0286 |
| rs7250869 | 19 | 33887405 | 0.690021 | C | T | 0.0114 | 0.0013 | -0.0359 | 0.0176 |
| rs2018519 | 19 | 35559787 | 0.181242 | C | T | 0.0203 | 0.0016 | 0.0130 | 0.0218 |
| rs11666245 | 19 | 38229926 | 0.953554 | G | A | 0.0164 | 0.0029 | -0.0187 | 0.0408 |
| rs5117 | 19 | 45418790 | 0.235459 | C | T | 0.0108 | 0.0015 | -0.0309 | 0.0222 |
| rs5112 | 19 | 45430280 | 0.532859 | G | C | 0.0091 | 0.0013 | 0.0755 | 0.0328 |
| rs73036519 | 19 | 45748362 | 0.699571 | G | C | 0.0086 | 0.0014 | 0.0075 | 0.0186 |
| rs34255979 | 19 | 46384830 | 0.121040 | T | C | 0.0282 | 0.0019 | -0.0342 | 0.0267 |
| rs62128735 | 19 | 48113604 | 0.696749 | A | G | 0.0101 | 0.0014 | 0.0163 | 0.0201 |
| rs59774409 | 19 | 50016748 | 0.082186 | T | C | 0.0180 | 0.0023 | -0.0195 | 0.0338 |
| rs4077285 | 19 | 56599405 | 0.906230 | G | C | 0.0115 | 0.0021 | 0.0212 | 0.0413 |
| rs11668201 | 19 | 59003632 | 0.189933 | T | A | 0.0086 | 0.0016 | -0.0100 | 0.0222 |
| rs1741344 | 20 | 4101800 | 0.634346 | T | C | 0.0071 | 0.0013 | -0.0231 | 0.0170 |
| rs13042148 | 20 | 32298286 | 0.845319 | C | T | 0.0157 | 0.0017 | 0.0504 | 0.0246 |
| rs6088776 | 20 | 33835773 | 0.860648 | T | C | 0.0105 | 0.0018 | -0.0062 | 0.0226 |
| rs2207132 | 20 | 39142516 | 0.966320 | G | A | 0.0276 | 0.0034 | -0.0517 | 0.2112 |
| rs6073431 | 20 | 43040569 | 0.531700 | T | C | 0.0168 | 0.0013 | 0.0355 | 0.0168 |
| rs4810580 | 20 | 45594295 | 0.782389 | T | G | 0.0096 | 0.0015 | -0.0576 | 0.0197 |
| rs16995626 | 20 | 49540925 | 0.072253 | C | T | 0.0180 | 0.0024 | -0.0255 | 0.0320 |
| rs1033667 | 22 | 29130300 | 0.299679 | T | C | 0.0092 | 0.0014 | 0.0226 | 0.0180 |
| rs5753111 | 22 | 30779211 | 0.290673 | T | C | 0.0129 | 0.0014 | 0.0233 | 0.0181 |
| rs5750131 | 22 | 36007421 | 0.615861 | G | A | 0.0068 | 0.0013 | -0.0058 | 0.0183 |
| rs3747207 | 22 | 44324855 | 0.214383 | A | G | 0.0173 | 0.0015 | 0.0135 | 0.0195 |
| SHBG_adj_BMI: sex hormone-binding globulin adjusted for BMI; SNP: single nucleotide polymorphism; Chr: chromosome; A1:effect allele; A2:other allele. | | | | | | | | | |

| **Table S4. The characteristic of total testosterone-associated index SNPs and their effect size with exposure and outcome.** | | | | | | | | | |
| --- | --- | --- | --- | --- | --- | --- | --- | --- | --- |
| **SNP** | **Chr** | **Position** | **Allele frequency** | **A1** | **A2** | **Exposure** | | **Outcome** | |
|  |  |  |  |  |  | **beta** | **se** | **beta** | **se** |
| rs10799713 | 1 | 22102328 | 0.211590 | G | C | 0.02710 | 0.00340 | 0.00720 | 0.01960 |
| rs7530117 | 1 | 31263360 | 0.369459 | T | C | 0.01780 | 0.00290 | 0.05070 | 0.01730 |
| rs4453027 | 1 | 41453453 | 0.574558 | G | T | 0.02260 | 0.00290 | 0.02810 | 0.01640 |
| rs1278526 | 1 | 50891213 | 0.524452 | A | C | 0.01930 | 0.00280 | -0.00750 | 0.01640 |
| rs7529520 | 1 | 57016477 | 0.485959 | C | G | 0.01830 | 0.00280 | -0.03260 | 0.01620 |
| rs505237 | 1 | 66738623 | 0.616031 | G | A | 0.01570 | 0.00290 | -0.04110 | 0.01710 |
| rs4294422 | 1 | 93104655 | 0.359334 | G | A | 0.01940 | 0.00290 | 0.09920 | 0.01710 |
| rs6684361 | 1 | 101737743 | 0.306953 | C | T | 0.07580 | 0.00300 | -0.05300 | 0.01750 |
| rs7519368 | 1 | 113150644 | 0.727188 | T | A | 0.03340 | 0.00310 | -0.00540 | 0.01920 |
| rs72693130 | 1 | 120013323 | 0.062638 | A | G | 0.03120 | 0.00590 | 0.03930 | 0.04790 |
| rs1870940 | 1 | 154984363 | 0.727398 | G | A | 0.02540 | 0.00320 | -0.00860 | 0.01950 |
| rs76830943 | 1 | 156009127 | 0.832755 | T | C | 0.02420 | 0.00380 | 0.00010 | 0.02850 |
| rs12564492 | 1 | 168234645 | 0.703240 | A | G | 0.01950 | 0.00310 | 0.00740 | 0.01770 |
| rs2062479 | 1 | 197868281 | 0.524073 | C | G | 0.01640 | 0.00280 | 0.01460 | 0.01730 |
| rs17043570 | 1 | 216905301 | 0.159257 | T | C | 0.02540 | 0.00380 | 0.01810 | 0.02260 |
| rs12078363 | 1 | 218537310 | 0.680824 | T | C | 0.02760 | 0.00300 | -0.03940 | 0.01780 |
| rs10910476 | 1 | 234734956 | 0.444104 | C | T | 0.01680 | 0.00280 | -0.00270 | 0.01850 |
| rs34269793 | 2 | 12555164 | 0.052515 | C | T | 0.06450 | 0.00630 | -0.04870 | 0.03730 |
| rs3771243 | 2 | 20412227 | 0.389909 | A | G | 0.02870 | 0.00290 | 0.01350 | 0.01720 |
| rs11892043 | 2 | 24990082 | 0.728129 | A | G | 0.02160 | 0.00310 | 0.02440 | 0.01820 |
| rs1260326 | 2 | 27730940 | 0.606511 | C | T | 0.03660 | 0.00290 | 0.00920 | 0.01660 |
| rs2374456 | 2 | 43271621 | 0.584175 | G | C | 0.02360 | 0.00290 | 0.01020 | 0.01710 |
| rs7575635 | 2 | 43515427 | 0.805662 | C | T | 0.03790 | 0.00350 | 0.01810 | 0.02060 |
| rs3136354 | 2 | 48031372 | 0.500744 | C | T | 0.02370 | 0.00280 | -0.01240 | 0.01680 |
| rs11125180 | 2 | 48942282 | 0.909007 | A | T | 0.03250 | 0.00490 | 0.00060 | 0.02660 |
| rs7573187 | 2 | 62536671 | 0.534724 | A | T | 0.01900 | 0.00280 | -0.00650 | 0.01680 |
| rs1009360 | 2 | 65276049 | 0.416067 | C | T | 0.01790 | 0.00280 | 0.03250 | 0.01660 |
| rs10865479 | 2 | 86095432 | 0.714977 | T | C | 0.02210 | 0.00310 | -0.02190 | 0.01800 |
| rs590097 | 2 | 111934107 | 0.646198 | G | T | 0.06060 | 0.00290 | -0.05070 | 0.01740 |
| rs10168169 | 2 | 112375425 | 0.812209 | T | C | 0.03330 | 0.00360 | -0.02080 | 0.02110 |
| rs59741822 | 2 | 136970398 | 0.923006 | G | A | 0.03300 | 0.00520 | 0.00850 | 0.04760 |
| rs58723250 | 2 | 178153430 | 0.200478 | T | C | 0.03510 | 0.00350 | 0.00440 | 0.02060 |
| rs36088520 | 2 | 197779590 | 0.104155 | T | C | 0.02830 | 0.00460 | -0.05960 | 0.03330 |
| rs873779 | 2 | 208773860 | 0.371709 | C | T | 0.01640 | 0.00290 | -0.00980 | 0.01670 |
| rs2011425 | 2 | 234627608 | 0.920604 | T | G | 0.03350 | 0.00520 | -0.00070 | 0.02700 |
| rs7618363 | 3 | 10545125 | 0.841085 | C | G | 0.03710 | 0.00380 | -0.02120 | 0.02320 |
| rs62231822 | 3 | 14423060 | 0.901557 | C | T | 0.04890 | 0.00470 | -0.01570 | 0.03420 |
| rs17201704 | 3 | 41105589 | 0.858666 | T | C | 0.04330 | 0.00410 | 0.01630 | 0.02380 |
| rs4067 | 3 | 51738256 | 0.854622 | G | A | 0.02430 | 0.00400 | 0.01400 | 0.02470 |
| rs9832502 | 3 | 72398979 | 0.248629 | A | G | 0.01750 | 0.00320 | -0.01570 | 0.01920 |
| rs167096 | 3 | 73874699 | 0.259620 | T | G | 0.01730 | 0.00320 | -0.01460 | 0.01920 |
| rs7633673 | 3 | 152084243 | 0.593857 | G | A | 0.02560 | 0.00280 | -0.01260 | 0.01670 |
| rs9850919 | 3 | 169177924 | 0.405319 | C | T | 0.02010 | 0.00290 | 0.01210 | 0.01660 |
| rs77822621 | 4 | 1008212 | 0.042221 | T | C | 0.04800 | 0.00700 | -0.03000 | 0.06400 |
| rs3849653 | 4 | 53860084 | 0.540807 | A | T | 0.01920 | 0.00280 | -0.00520 | 0.01710 |
| rs4632729 | 4 | 69946004 | 0.545410 | A | G | 0.02760 | 0.00280 | -0.02860 | 0.01630 |
| rs1229984 | 4 | 100239319 | 0.976316 | C | T | 0.04900 | 0.00920 | 0.02240 | 0.07190 |
| rs4586943 | 4 | 102366529 | 0.282112 | A | C | 0.01840 | 0.00310 | 0.02520 | 0.01820 |
| rs2903385 | 4 | 106094427 | 0.485657 | A | G | 0.02460 | 0.00280 | -0.00020 | 0.01970 |
| rs4245930 | 4 | 109038654 | 0.367501 | G | A | 0.02540 | 0.00290 | 0.08440 | 0.01680 |
| rs371162363 | 4 | 177614117 | 0.904135 | G | A | 0.03920 | 0.00500 | -0.02590 | 0.13720 |
| rs112694713 | 5 | 35247932 | 0.986488 | A | G | 0.10700 | 0.01240 | -0.09930 | 0.06610 |
| rs9687846 | 5 | 55861894 | 0.800305 | G | A | 0.02640 | 0.00350 | 0.03030 | 0.02140 |
| rs4431325 | 5 | 76461706 | 0.058979 | T | C | 0.04970 | 0.00600 | -0.00880 | 0.03800 |
| rs1119208 | 5 | 76488613 | 0.648496 | C | T | 0.03050 | 0.00290 | 0.03270 | 0.01690 |
| rs784420 | 5 | 77987524 | 0.288700 | G | A | 0.03900 | 0.00310 | 0.01380 | 0.01870 |
| rs11948639 | 5 | 122741746 | 0.434320 | T | C | 0.01710 | 0.00280 | 0.02190 | 0.01640 |
| rs12658172 | 5 | 124205385 | 0.841514 | G | C | 0.05170 | 0.00390 | 0.00170 | 0.02400 |
| rs13184921 | 5 | 127870642 | 0.753156 | T | C | 0.02550 | 0.00330 | -0.00240 | 0.01920 |
| rs3776299 | 5 | 142507651 | 0.549186 | G | A | 0.01710 | 0.00280 | -0.01180 | 0.01630 |
| rs13153019 | 5 | 176782218 | 0.249244 | C | T | 0.02430 | 0.00330 | 0.08250 | 0.01920 |
| rs75217853 | 6 | 1377048 | 0.097925 | A | G | 0.03500 | 0.00470 | -0.08060 | 0.03030 |
| rs267190 | 6 | 7842121 | 0.577753 | G | T | 0.01520 | 0.00280 | 0.04000 | 0.01650 |
| rs6904345 | 6 | 24697424 | 0.634032 | T | C | 0.01650 | 0.00290 | -0.03360 | 0.01710 |
| rs487624 | 6 | 25879539 | 0.568302 | C | A | 0.01930 | 0.00280 | -0.00140 | 0.01600 |
| rs2517582 | 6 | 30808762 | 0.384303 | T | C | 0.02770 | 0.00290 | -0.14290 | 0.01690 |
| rs184265581 | 6 | 31900754 | 0.012357 | C | G | 0.13230 | 0.01270 | 0.09900 | 0.14570 |
| rs1214761 | 6 | 43354431 | 0.679032 | G | A | 0.03110 | 0.00300 | 0.00610 | 0.01720 |
| rs2608652 | 6 | 52642768 | 0.525165 | T | C | 0.01540 | 0.00280 | -0.00410 | 0.01650 |
| rs1032388 | 6 | 119171866 | 0.779374 | C | T | 0.05440 | 0.00340 | -0.06090 | 0.02000 |
| rs2473140 | 6 | 144620688 | 0.090432 | C | T | 0.02920 | 0.00490 | 0.06870 | 0.02790 |
| rs287884 | 6 | 157124080 | 0.443895 | A | T | 0.01860 | 0.00280 | -0.00630 | 0.01650 |
| rs9457466 | 6 | 159224561 | 0.362690 | A | T | 0.01700 | 0.00290 | 0.01450 | 0.01690 |
| rs2344744 | 6 | 170588559 | 0.414681 | G | T | 0.01780 | 0.00280 | -0.01950 | 0.01810 |
| rs28612846 | 7 | 25950880 | 0.743341 | G | A | 0.01560 | 0.00320 | -0.06020 | 0.01910 |
| rs6460528 | 7 | 69218337 | 0.454495 | T | C | 0.01530 | 0.00280 | 0.01310 | 0.01680 |
| rs13229619 | 7 | 73030175 | 0.129368 | A | G | 0.05380 | 0.00420 | 0.02210 | 0.02980 |
| rs1872930 | 7 | 137801444 | 0.792539 | T | C | 0.05140 | 0.00350 | 0.02380 | 0.02020 |
| rs9638084 | 7 | 156311745 | 0.603529 | G | A | 0.02060 | 0.00290 | -0.00450 | 0.01720 |
| rs17362923 | 8 | 5546824 | 0.189026 | G | C | 0.02330 | 0.00360 | 0.02140 | 0.02070 |
| rs6997799 | 8 | 10606421 | 0.234469 | C | A | 0.02360 | 0.00330 | -0.01020 | 0.01960 |
| rs56109436 | 8 | 11543663 | 0.098716 | G | C | 0.02590 | 0.00470 | 0.00750 | 0.02860 |
| rs17053931 | 8 | 25417528 | 0.198880 | A | G | 0.02060 | 0.00350 | -0.03640 | 0.02010 |
| rs11782259 | 8 | 37532984 | 0.910616 | A | G | 0.03080 | 0.00490 | 0.01710 | 0.03220 |
| rs10108398 | 8 | 59440824 | 0.278554 | G | A | 0.01810 | 0.00320 | 0.01260 | 0.02050 |
| rs13269725 | 8 | 72459889 | 0.921111 | A | G | 0.03000 | 0.00520 | 0.02060 | 0.03550 |
| rs1660322 | 8 | 101277640 | 0.686984 | T | C | 0.02330 | 0.00300 | 0.02980 | 0.01790 |
| rs11774829 | 8 | 105978368 | 0.101260 | A | T | 0.05600 | 0.00470 | -0.00880 | 0.03190 |
| rs4736359 | 8 | 143987427 | 0.442771 | T | G | 0.03700 | 0.00280 | 0.01140 | 0.01630 |
| rs12683780 | 9 | 16252807 | 0.668430 | A | C | 0.03090 | 0.00310 | 0.00480 | 0.01810 |
| rs4961485 | 9 | 16360889 | 0.933562 | T | C | 0.03910 | 0.00560 | -0.03400 | 0.03380 |
| rs61237993 | 9 | 34130435 | 0.127933 | A | G | 0.04000 | 0.00420 | 0.01520 | 0.02410 |
| rs10821415 | 9 | 97713459 | 0.578972 | C | A | 0.01460 | 0.00280 | 0.00560 | 0.01660 |
| rs1547308 | 9 | 114603240 | 0.800022 | C | T | 0.03230 | 0.00350 | -0.02260 | 0.02050 |
| rs10817260 | 9 | 114828332 | 0.809595 | C | T | 0.04300 | 0.00360 | 0.02690 | 0.02170 |
| rs494242 | 9 | 136145118 | 0.659060 | C | T | 0.01690 | 0.00300 | 0.02990 | 0.01680 |
| rs35249079 | 10 | 1037737 | 0.530644 | T | G | 0.02240 | 0.00280 | 0.01710 | 0.01620 |
| rs36032941 | 10 | 5062752 | 0.704004 | C | A | 0.06280 | 0.00310 | -0.02610 | 0.02050 |
| rs1171617 | 10 | 61467182 | 0.767086 | T | G | 0.05230 | 0.00330 | -0.00150 | 0.02090 |
| rs10740131 | 10 | 65271488 | 0.473158 | T | A | 0.02830 | 0.00280 | -0.00770 | 0.01630 |
| rs674486 | 10 | 69819917 | 0.350394 | C | T | 0.01760 | 0.00290 | 0.02620 | 0.01830 |
| rs35199395 | 10 | 70983936 | 0.693866 | C | G | 0.01700 | 0.00300 | 0.03480 | 0.01770 |
| rs2147419 | 10 | 90759916 | 0.717874 | T | G | 0.01820 | 0.00310 | 0.00670 | 0.01800 |
| rs111328885 | 10 | 96823812 | 0.878145 | C | A | 0.03260 | 0.00430 | -0.04990 | 0.02550 |
| rs11191421 | 10 | 104623053 | 0.758129 | C | G | 0.03740 | 0.00330 | -0.00950 | 0.01890 |
| rs11191801 | 10 | 105532165 | 0.708091 | A | C | 0.02340 | 0.00310 | -0.04190 | 0.01790 |
| rs440150 | 11 | 2936657 | 0.093409 | G | A | 0.02570 | 0.00490 | -0.02250 | 0.02880 |
| rs11024458 | 11 | 18082483 | 0.728256 | A | G | 0.01890 | 0.00320 | 0.00900 | 0.01920 |
| rs10501081 | 11 | 27236061 | 0.137669 | G | C | 0.02160 | 0.00410 | -0.02610 | 0.02300 |
| rs11031005 | 11 | 30226356 | 0.142867 | C | T | 0.03260 | 0.00400 | -0.03820 | 0.02310 |
| rs1939769 | 11 | 62914202 | 0.066412 | A | G | 0.08330 | 0.00570 | 0.02060 | 0.03630 |
| rs312023 | 11 | 68097049 | 0.472423 | G | A | 0.02790 | 0.00280 | -0.02450 | 0.01690 |
| rs171021 | 11 | 72317557 | 0.702846 | C | T | 0.03840 | 0.00310 | -0.03040 | 0.01780 |
| rs11235688 | 11 | 72947934 | 0.583068 | G | A | 0.02230 | 0.00290 | 0.00100 | 0.01710 |
| rs75848431 | 11 | 123325111 | 0.151708 | T | C | 0.03170 | 0.00390 | -0.01520 | 0.02810 |
| rs76299412 | 11 | 128269098 | 0.152614 | A | G | 0.03490 | 0.00390 | -0.01260 | 0.02320 |
| rs881613 | 12 | 12738486 | 0.301183 | A | G | 0.01720 | 0.00310 | -0.00890 | 0.02500 |
| rs4149056 | 12 | 21331549 | 0.151403 | C | T | 0.02900 | 0.00390 | 0.01960 | 0.02230 |
| rs117913411 | 12 | 48254353 | 0.966332 | T | A | 0.04810 | 0.00790 | -0.03880 | 0.04940 |
| rs35801460 | 12 | 49180828 | 0.796971 | G | A | 0.02040 | 0.00350 | -0.00730 | 0.02120 |
| rs7977247 | 12 | 107259470 | 0.578252 | T | C | 0.01830 | 0.00280 | -0.02890 | 0.01640 |
| rs35427 | 12 | 115556307 | 0.616743 | T | G | 0.01710 | 0.00300 | 0.01990 | 0.01720 |
| rs1169289 | 12 | 121416622 | 0.439895 | G | C | 0.01950 | 0.00280 | 0.00600 | 0.01730 |
| rs837493 | 12 | 125075660 | 0.451504 | G | A | 0.01580 | 0.00280 | 0.00580 | 0.01650 |
| rs9506725 | 13 | 22314146 | 0.629106 | T | C | 0.05230 | 0.00290 | -0.01900 | 0.01690 |
| rs9552597 | 13 | 22690127 | 0.190986 | A | G | 0.03540 | 0.00360 | 0.01470 | 0.02050 |
| rs4943729 | 13 | 32353100 | 0.518398 | A | C | 0.01540 | 0.00280 | 0.00030 | 0.01640 |
| rs17764067 | 13 | 33728353 | 0.780329 | G | A | 0.02080 | 0.00340 | -0.02670 | 0.02020 |
| rs17245822 | 13 | 73131694 | 0.372728 | C | A | 0.02840 | 0.00290 | -0.01820 | 0.01690 |
| rs9599996 | 13 | 73203811 | 0.598724 | T | G | 0.01740 | 0.00290 | 0.00790 | 0.01650 |
| rs7342537 | 14 | 21555063 | 0.018067 | G | A | 0.10690 | 0.01060 | 0.01040 | 0.05770 |
| rs59397130 | 14 | 64898076 | 0.026261 | G | A | 0.05620 | 0.00880 | 0.04080 | 0.05000 |
| rs61987429 | 14 | 65606248 | 0.649040 | C | T | 0.01750 | 0.00300 | -0.00640 | 0.01740 |
| rs1314911 | 14 | 68698230 | 0.858906 | A | G | 0.02590 | 0.00400 | -0.02770 | 0.02260 |
| rs72731535 | 14 | 69237902 | 0.822269 | G | A | 0.02540 | 0.00370 | -0.07440 | 0.02130 |
| rs112635299 | 14 | 94838142 | 0.979102 | G | T | 0.06210 | 0.00980 | -0.10190 | 0.06060 |
| rs12436785 | 14 | 98550490 | 0.416727 | C | T | 0.03000 | 0.00280 | -0.00420 | 0.01680 |
| rs10147094 | 14 | 99727591 | 0.614111 | A | G | 0.02200 | 0.00290 | -0.03000 | 0.01890 |
| rs11638521 | 15 | 40360314 | 0.347256 | T | C | 0.06400 | 0.00290 | -0.00360 | 0.01710 |
| rs61661087 | 15 | 40720543 | 0.524200 | C | T | 0.02830 | 0.00280 | 0.01650 | 0.01630 |
| rs72738949 | 15 | 50668999 | 0.420114 | T | A | 0.01890 | 0.00290 | 0.02070 | 0.01740 |
| rs2113944 | 15 | 60977882 | 0.222783 | T | C | 0.02720 | 0.00340 | -0.00530 | 0.02010 |
| rs12708515 | 15 | 75454879 | 0.364201 | G | C | 0.02140 | 0.00290 | 0.01360 | 0.01770 |
| rs4464040 | 15 | 79840557 | 0.850331 | C | T | 0.04460 | 0.00390 | 0.01900 | 0.02340 |
| rs12900736 | 15 | 85558140 | 0.830190 | C | T | 0.03970 | 0.00370 | 0.00260 | 0.02090 |
| rs2074585 | 15 | 91009484 | 0.513762 | A | G | 0.01600 | 0.00280 | 0.07200 | 0.01890 |
| rs437115 | 16 | 4156423 | 0.564624 | T | C | 0.02790 | 0.00280 | -0.01850 | 0.01640 |
| rs8045779 | 16 | 12907061 | 0.836014 | T | C | 0.02560 | 0.00380 | -0.00340 | 0.02220 |
| rs8044588 | 16 | 67420328 | 0.088608 | C | G | 0.02850 | 0.00490 | 0.01080 | 0.03320 |
| rs58072681 | 16 | 81590541 | 0.070342 | C | T | 0.10820 | 0.00550 | 0.06530 | 0.03250 |
| rs187370584 | 17 | 2309972 | 0.986727 | A | G | 0.06620 | 0.01240 | 0.16610 | 0.45570 |
| rs9898480 | 17 | 6525861 | 0.569124 | T | C | 0.01830 | 0.00280 | -0.00470 | 0.01630 |
| rs62059839 | 17 | 7533015 | 0.260760 | T | C | 0.03250 | 0.00320 | -0.04340 | 0.01970 |
| rs2270445 | 17 | 8219478 | 0.486960 | G | A | 0.01720 | 0.00280 | 0.01080 | 0.01640 |
| rs1242518 | 17 | 17387899 | 0.747432 | T | C | 0.02050 | 0.00320 | 0.01420 | 0.01850 |
| rs232159 | 17 | 63708321 | 0.349369 | C | T | 0.01750 | 0.00300 | 0.01970 | 0.01720 |
| rs34931250 | 17 | 66879927 | 0.939450 | C | T | 0.05450 | 0.00590 | -0.04800 | 0.07780 |
| rs28421540 | 18 | 3818842 | 0.713516 | A | C | 0.03090 | 0.00310 | -0.00570 | 0.01830 |
| rs2186945 | 18 | 13921536 | 0.161688 | C | T | 0.02490 | 0.00380 | 0.01890 | 0.02210 |
| rs34163044 | 18 | 51851616 | 0.419375 | A | C | 0.01670 | 0.00290 | -0.03530 | 0.01720 |
| rs112367565 | 18 | 71883232 | 0.046207 | A | C | 0.04910 | 0.00670 | -0.07970 | 0.07140 |
| rs9319895 | 18 | 71896469 | 0.515052 | A | G | 0.02350 | 0.00290 | 0.00360 | 0.02550 |
| rs117327231 | 18 | 71916636 | 0.022790 | A | C | 0.17310 | 0.00940 | 0.04760 | 0.05650 |
| rs12977787 | 19 | 1814025 | 0.541424 | A | G | 0.01530 | 0.00280 | 0.00750 | 0.01700 |
| rs8111359 | 19 | 10471462 | 0.904763 | C | T | 0.05980 | 0.00480 | -0.01250 | 0.02980 |
| rs4804181 | 19 | 12509536 | 0.220984 | C | A | 0.03690 | 0.00340 | -0.05750 | 0.02370 |
| rs11673591 | 19 | 41985931 | 0.252020 | A | T | 0.02850 | 0.00320 | 0.03280 | 0.01880 |
| rs7256920 | 19 | 46203083 | 0.515161 | G | A | 0.01890 | 0.00280 | 0.02320 | 0.01690 |
| rs2879910 | 19 | 48335029 | 0.481440 | C | T | 0.01600 | 0.00280 | 0.02230 | 0.01710 |
| rs75287599 | 19 | 49517140 | 0.077324 | T | C | 0.04330 | 0.00530 | 0.02670 | 0.03010 |
| rs11697333 | 20 | 31189078 | 0.674464 | T | C | 0.01920 | 0.00300 | -0.02710 | 0.01810 |
| rs1883711 | 20 | 39179822 | 0.968358 | G | C | 0.05000 | 0.00820 | 0.15140 | 0.42480 |
| rs6020423 | 20 | 48909667 | 0.760288 | C | T | 0.03980 | 0.00330 | -0.00970 | 0.01920 |
| rs6127099 | 20 | 52731402 | 0.721214 | A | T | 0.01770 | 0.00320 | -0.05810 | 0.02060 |
| rs6100174 | 20 | 57280575 | 0.624844 | C | T | 0.02040 | 0.00290 | 0.00480 | 0.01700 |
| rs8126001 | 20 | 62711459 | 0.489930 | T | C | 0.01970 | 0.00280 | -0.00690 | 0.01840 |
| rs2824138 | 21 | 18294462 | 0.182318 | C | T | 0.02600 | 0.00360 | -0.05150 | 0.02120 |
| rs12185851 | 21 | 43372219 | 0.231467 | C | T | 0.02140 | 0.00330 | -0.01110 | 0.02010 |
| rs8184986 | 22 | 29127224 | 0.865764 | A | T | 0.02340 | 0.00420 | -0.02700 | 0.02640 |
| rs12628709 | 22 | 29647854 | 0.109262 | G | A | 0.02640 | 0.00450 | -0.03580 | 0.02660 |
| rs4820829 | 22 | 30524248 | 0.976727 | C | T | 0.05760 | 0.00930 | 0.00070 | 0.05820 |
| rs5751229 | 22 | 42545221 | 0.228462 | A | G | 0.02510 | 0.00340 | -0.02550 | 0.02050 |
| rs6008259 | 22 | 46633782 | 0.821105 | G | A | 0.03380 | 0.00370 | -0.00900 | 0.02140 |
| SNP: single nucleotide polymorphism; Chr: chromosome; A1:effect allele; A2:other allele. | | | | | | | | | |

| **Table S5. The characteristic of bioavailable testosterone-associated index SNPs and their effect sizes with exposure and outcome.** | | | | | | | | | |
| --- | --- | --- | --- | --- | --- | --- | --- | --- | --- |
| **SNP** | **Chr** | **Position** | **Allele frequency** | **A1** | **A2** | **Exposure** | | **Outcome** | |
|  |  |  |  |  |  | **beta** | **se** | **beta** | **se** |
| rs1989147 | 1 | 7909373 | 0.806791 | C | T | 0.0236 | 0.0032 | 0.0039 | 0.0215 |
| rs114165349 | 1 | 27021913 | 0.023696 | C | G | 0.0931 | 0.0084 | 0.0508 | 0.0592 |
| rs12135478 | 1 | 39539043 | 0.331026 | G | A | 0.0151 | 0.0027 | 0.0462 | 0.0177 |
| rs2391139 | 1 | 92727610 | 0.201753 | T | G | 0.0247 | 0.0031 | 0.0089 | 0.0203 |
| rs7418101 | 1 | 93756192 | 0.622174 | G | A | 0.0155 | 0.0026 | -0.0122 | 0.0170 |
| rs6684361 | 1 | 101737743 | 0.306970 | C | T | 0.0395 | 0.0028 | -0.0530 | 0.0175 |
| rs2282248 | 1 | 111736594 | 0.328468 | T | C | 0.0151 | 0.0027 | -0.0323 | 0.0183 |
| rs351370 | 1 | 113054659 | 0.586551 | T | C | 0.0162 | 0.0026 | -0.0058 | 0.0189 |
| rs12564492 | 1 | 168234645 | 0.703211 | A | G | 0.0153 | 0.0028 | 0.0074 | 0.0177 |
| rs2266782 | 1 | 171076966 | 0.416820 | A | G | 0.0161 | 0.0026 | -0.0034 | 0.0162 |
| rs2152318 | 1 | 179293511 | 0.244706 | T | C | 0.0318 | 0.0029 | -0.0007 | 0.0187 |
| rs34269793 | 2 | 12555164 | 0.052736 | C | T | 0.0333 | 0.0057 | -0.0487 | 0.0373 |
| rs62144584 | 2 | 25004937 | 0.728206 | T | C | 0.0189 | 0.0028 | 0.0230 | 0.0183 |
| rs2374456 | 2 | 43271621 | 0.583892 | G | C | 0.0158 | 0.0026 | 0.0102 | 0.0171 |
| rs13030651 | 2 | 43508205 | 0.448832 | G | A | 0.0166 | 0.0025 | -0.0382 | 0.0165 |
| rs13020003 | 2 | 48976886 | 0.326875 | C | A | 0.0170 | 0.0027 | 0.0096 | 0.0176 |
| rs9636441 | 2 | 62437709 | 0.289842 | C | T | 0.0168 | 0.0028 | -0.0100 | 0.0189 |
| rs13430258 | 2 | 62535082 | 0.534478 | G | T | 0.0155 | 0.0025 | -0.0072 | 0.0167 |
| rs590097 | 2 | 111934107 | 0.646468 | G | T | 0.0340 | 0.0026 | -0.0507 | 0.0174 |
| rs11683361 | 2 | 112380559 | 0.806537 | G | C | 0.0223 | 0.0032 | -0.0307 | 0.0211 |
| rs1515098 | 2 | 227073854 | 0.680427 | T | C | 0.0153 | 0.0027 | 0.0534 | 0.0176 |
| rs3732218 | 2 | 234627304 | 0.918354 | G | A | 0.0252 | 0.0046 | 0.0010 | 0.0293 |
| rs573833 | 3 | 10534247 | 0.463062 | T | C | 0.0148 | 0.0025 | -0.0045 | 0.0167 |
| rs4135247 | 3 | 12396588 | 0.433284 | G | A | 0.0154 | 0.0026 | 0.0289 | 0.0169 |
| rs62231822 | 3 | 14423060 | 0.901312 | C | T | 0.0289 | 0.0043 | -0.0157 | 0.0342 |
| rs13072623 | 3 | 20163556 | 0.432668 | G | A | 0.0154 | 0.0026 | 0.0270 | 0.0167 |
| rs6792725 | 3 | 24520283 | 0.308073 | A | G | 0.0173 | 0.0028 | 0.0076 | 0.0180 |
| rs6788984 | 3 | 41107173 | 0.856468 | A | G | 0.0201 | 0.0036 | 0.0212 | 0.0231 |
| rs62263023 | 3 | 48260304 | 0.091487 | A | T | 0.0281 | 0.0044 | 0.0393 | 0.0341 |
| rs6772177 | 3 | 52497778 | 0.171599 | T | C | 0.0204 | 0.0034 | 0.0286 | 0.0267 |
| rs687339 | 3 | 135932359 | 0.772974 | T | C | 0.0396 | 0.0030 | 0.0089 | 0.0196 |
| rs7633673 | 3 | 152084243 | 0.594220 | G | A | 0.0184 | 0.0026 | -0.0126 | 0.0167 |
| rs4368453 | 3 | 156852141 | 0.301677 | T | C | 0.0171 | 0.0028 | 0.0215 | 0.0179 |
| rs4690098 | 4 | 3447156 | 0.235337 | T | C | 0.0266 | 0.0030 | 0.0177 | 0.0199 |
| rs114303452 | 4 | 3449915 | 0.988567 | A | G | 0.0919 | 0.0123 | 0.0124 | 0.1334 |
| rs12645584 | 4 | 17919066 | 0.731368 | T | C | 0.0169 | 0.0029 | 0.0359 | 0.0185 |
| rs17287714 | 4 | 159871166 | 0.881885 | T | C | 0.0221 | 0.0040 | -0.0386 | 0.0343 |
| rs12189146 | 5 | 35239886 | 0.949888 | G | A | 0.0320 | 0.0058 | -0.0532 | 0.0359 |
| rs40270 | 5 | 55804552 | 0.773334 | C | A | 0.0190 | 0.0030 | -0.0032 | 0.0199 |
| rs34341 | 5 | 74934009 | 0.576182 | T | A | 0.0149 | 0.0026 | -0.0179 | 0.0172 |
| rs1119208 | 5 | 76488613 | 0.648622 | C | T | 0.0175 | 0.0027 | 0.0327 | 0.0169 |
| rs168189 | 5 | 77974427 | 0.279591 | T | C | 0.0203 | 0.0028 | 0.0114 | 0.0199 |
| rs12658172 | 5 | 124205385 | 0.841810 | G | C | 0.0303 | 0.0035 | 0.0017 | 0.0240 |
| rs73350117 | 5 | 127877729 | 0.827577 | A | C | 0.0203 | 0.0034 | -0.0006 | 0.0239 |
| rs1432679 | 5 | 158244083 | 0.446467 | C | T | 0.0134 | 0.0025 | -0.0343 | 0.0164 |
| rs13153019 | 5 | 176782218 | 0.249291 | C | T | 0.0177 | 0.0029 | 0.0825 | 0.0192 |
| rs9379084 | 6 | 7231843 | 0.115835 | A | G | 0.0225 | 0.0041 | -0.0844 | 0.0298 |
| rs4712976 | 6 | 25842203 | 0.732442 | C | T | 0.0186 | 0.0029 | -0.0327 | 0.0182 |
| rs199746610 | 6 | 34364965 | 0.966894 | C | A | 0.0409 | 0.0072 | -0.0269 | 0.0356 |
| rs62396733 | 6 | 41679691 | 0.137117 | T | C | 0.0213 | 0.0037 | 0.0188 | 0.0246 |
| rs1214759 | 6 | 43352980 | 0.678718 | G | A | 0.0181 | 0.0027 | 0.0060 | 0.0171 |
| rs2397112 | 6 | 52684333 | 0.573791 | A | G | 0.0161 | 0.0026 | -0.0079 | 0.0164 |
| rs9399469 | 6 | 144318529 | 0.622027 | A | T | 0.0198 | 0.0026 | 0.0097 | 0.0168 |
| rs4869893 | 6 | 157117322 | 0.281972 | C | A | 0.0155 | 0.0028 | -0.0088 | 0.0181 |
| rs381194 | 6 | 160712194 | 0.528176 | G | A | 0.0127 | 0.0025 | 0.0041 | 0.0164 |
| rs7780066 | 7 | 137801915 | 0.793042 | A | G | 0.0228 | 0.0031 | 0.0236 | 0.0202 |
| rs9987289 | 8 | 9183358 | 0.092071 | A | G | 0.0337 | 0.0044 | -0.0223 | 0.0291 |
| rs10504255 | 8 | 59398461 | 0.336853 | G | A | 0.0174 | 0.0027 | 0.0128 | 0.0171 |
| rs113347955 | 8 | 97136852 | 0.976005 | G | A | 0.0446 | 0.0084 | -0.0110 | 0.0556 |
| rs35783704 | 8 | 105966258 | 0.101475 | A | G | 0.0327 | 0.0042 | -0.0160 | 0.0366 |
| rs11784903 | 8 | 116979531 | 0.525780 | C | T | 0.0136 | 0.0025 | -0.0134 | 0.0164 |
| rs12543598 | 8 | 143955318 | 0.445787 | T | G | 0.0199 | 0.0025 | 0.0111 | 0.0163 |
| rs12683780 | 9 | 16252807 | 0.668616 | A | C | 0.0179 | 0.0028 | 0.0048 | 0.0181 |
| rs10757893 | 9 | 29599001 | 0.575224 | A | G | 0.0137 | 0.0026 | -0.0202 | 0.0180 |
| rs61237993 | 9 | 34130435 | 0.127902 | A | G | 0.0294 | 0.0038 | 0.0152 | 0.0241 |
| rs61856128 | 10 | 5063382 | 0.703561 | C | A | 0.0346 | 0.0028 | -0.0260 | 0.0204 |
| rs1171617 | 10 | 61467182 | 0.766787 | T | G | 0.0295 | 0.0030 | -0.0015 | 0.0209 |
| rs7089122 | 10 | 93647412 | 0.177718 | T | C | 0.0244 | 0.0033 | 0.0324 | 0.0209 |
| rs9527 | 10 | 104623578 | 0.758463 | C | T | 0.0230 | 0.0030 | -0.0095 | 0.0188 |
| rs7078330 | 10 | 122834163 | 0.087856 | T | C | 0.0313 | 0.0045 | 0.0125 | 0.0287 |
| rs6486122 | 11 | 13361524 | 0.689797 | T | C | 0.0167 | 0.0027 | -0.0041 | 0.0177 |
| rs11031005 | 11 | 30226356 | 0.143658 | C | T | 0.0230 | 0.0036 | -0.0382 | 0.0231 |
| rs113172275 | 11 | 62905115 | 0.066134 | C | T | 0.0492 | 0.0051 | 0.0202 | 0.0367 |
| rs3814707 | 11 | 65560785 | 0.758298 | G | A | 0.0195 | 0.0030 | 0.0516 | 0.0195 |
| rs171021 | 11 | 72317557 | 0.703178 | C | T | 0.0181 | 0.0028 | -0.0304 | 0.0178 |
| rs11235688 | 11 | 72947934 | 0.583063 | G | A | 0.0165 | 0.0026 | 0.0010 | 0.0171 |
| rs850294 | 11 | 123437669 | 0.113609 | T | C | 0.0337 | 0.0040 | -0.0042 | 0.0276 |
| rs76895963 | 12 | 4384844 | 0.979035 | T | G | 0.0875 | 0.0097 | 0.0578 | 0.2166 |
| rs4149056 | 12 | 21331549 | 0.151223 | C | T | 0.0426 | 0.0035 | 0.0196 | 0.0223 |
| rs11047261 | 12 | 24257228 | 0.041384 | G | A | 0.0366 | 0.0064 | 0.0210 | 0.0396 |
| rs56205943 | 12 | 57679414 | 0.759004 | G | A | 0.0209 | 0.0030 | -0.0016 | 0.0199 |
| rs6538437 | 12 | 93989735 | 0.289023 | T | A | 0.0162 | 0.0028 | 0.0015 | 0.0186 |
| rs7301634 | 12 | 98901200 | 0.810267 | A | G | 0.0191 | 0.0032 | 0.0136 | 0.0212 |
| rs10778215 | 12 | 103537266 | 0.472109 | A | T | 0.0243 | 0.0025 | -0.0079 | 0.0163 |
| rs7314285 | 12 | 111522026 | 0.931867 | T | G | 0.0375 | 0.0050 | -0.0411 | 0.0342 |
| rs7139079 | 12 | 121415293 | 0.407287 | G | A | 0.0221 | 0.0026 | 0.0115 | 0.0167 |
| rs2954111 | 12 | 122134415 | 0.361450 | C | T | 0.0167 | 0.0026 | 0.0050 | 0.0172 |
| rs629042 | 13 | 22318506 | 0.607001 | C | G | 0.0284 | 0.0026 | -0.0178 | 0.0167 |
| rs17245822 | 13 | 73131694 | 0.373082 | C | A | 0.0139 | 0.0026 | -0.0182 | 0.0169 |
| rs17128091 | 14 | 23714682 | 0.744091 | G | C | 0.0163 | 0.0029 | 0.0189 | 0.0205 |
| rs11621792 | 14 | 24871926 | 0.452392 | T | C | 0.0245 | 0.0026 | 0.0267 | 0.0174 |
| rs112635299 | 14 | 94838142 | 0.979033 | G | T | 0.1023 | 0.0089 | -0.1019 | 0.0606 |
| rs17580 | 14 | 94847262 | 0.952028 | T | A | 0.0402 | 0.0059 | -0.0689 | 0.0644 |
| rs7183977 | 15 | 40377092 | 0.347710 | C | T | 0.0286 | 0.0027 | -0.0054 | 0.0176 |
| rs10851395 | 15 | 40718534 | 0.524015 | C | T | 0.0189 | 0.0025 | 0.0164 | 0.0162 |
| rs149624078 | 15 | 53728710 | 0.014014 | T | C | 0.0791 | 0.0111 | 0.2254 | 0.2124 |
| rs62025141 | 15 | 79850183 | 0.855415 | A | G | 0.0263 | 0.0037 | 0.0131 | 0.0238 |
| rs71397837 | 15 | 85563561 | 0.829736 | G | A | 0.0196 | 0.0034 | 0.0001 | 0.0319 |
| rs56332871 | 15 | 96714816 | 0.727843 | C | A | 0.0299 | 0.0029 | -0.0301 | 0.0188 |
| rs879619 | 16 | 4015046 | 0.864046 | G | A | 0.0211 | 0.0037 | 0.0316 | 0.0247 |
| rs388430 | 16 | 4135562 | 0.683193 | C | T | 0.0167 | 0.0027 | -0.0394 | 0.0174 |
| rs8046391 | 16 | 30836648 | 0.272182 | C | G | 0.0171 | 0.0028 | -0.0122 | 0.0187 |
| rs58072681 | 16 | 81590541 | 0.070023 | C | T | 0.0611 | 0.0050 | 0.0653 | 0.0325 |
| rs11078597 | 17 | 1618363 | 0.813954 | T | C | 0.0193 | 0.0032 | -0.0468 | 0.0240 |
| rs727428 | 17 | 7537792 | 0.444254 | T | C | 0.0948 | 0.0025 | 0.0241 | 0.0178 |
| rs11653686 | 17 | 47362991 | 0.913205 | C | T | 0.0657 | 0.0045 | -0.0242 | 0.0303 |
| rs4793788 | 17 | 53381796 | 0.363867 | C | A | 0.0159 | 0.0026 | 0.0011 | 0.0165 |
| rs8178824 | 17 | 64224775 | 0.029755 | T | C | 0.0578 | 0.0074 | 0.0571 | 0.1403 |
| rs34931250 | 17 | 66879927 | 0.939244 | C | T | 0.0399 | 0.0053 | -0.0480 | 0.0778 |
| rs2587507 | 17 | 77790135 | 0.506658 | C | T | 0.0145 | 0.0025 | -0.0054 | 0.0163 |
| rs117327231 | 18 | 71916636 | 0.022901 | A | C | 0.0972 | 0.0085 | 0.0476 | 0.0565 |
| rs7239564 | 18 | 71967031 | 0.856074 | C | T | 0.0308 | 0.0036 | 0.0111 | 0.0239 |
| rs1640272 | 19 | 2800192 | 0.712846 | A | T | 0.0212 | 0.0028 | -0.0392 | 0.0180 |
| rs7248104 | 19 | 7224431 | 0.584360 | G | A | 0.0157 | 0.0026 | -0.0150 | 0.0165 |
| rs8111359 | 19 | 10471462 | 0.904645 | C | T | 0.0343 | 0.0044 | -0.0125 | 0.0298 |
| rs1688043 | 19 | 35553341 | 0.066263 | C | T | 0.0407 | 0.0051 | -0.0237 | 0.0334 |
| rs11879227 | 19 | 46193351 | 0.816917 | A | G | 0.0201 | 0.0033 | 0.0729 | 0.0217 |
| rs34255979 | 19 | 46384830 | 0.878920 | C | T | 0.0228 | 0.0039 | 0.0342 | 0.0267 |
| rs6073431 | 20 | 43040569 | 0.468310 | C | T | 0.0146 | 0.0026 | -0.0355 | 0.0168 |
| rs6020423 | 20 | 48909667 | 0.759653 | C | T | 0.0250 | 0.0030 | -0.0097 | 0.0192 |
| rs62223042 | 21 | 40683740 | 0.374730 | G | A | 0.0153 | 0.0026 | 0.0108 | 0.0172 |
| rs738409 | 22 | 44324727 | 0.783867 | C | G | 0.0171 | 0.0031 | -0.0139 | 0.0196 |
| rs6008259 | 22 | 46633782 | 0.820867 | G | A | 0.0208 | 0.0033 | -0.0090 | 0.0214 |
| SNP: single nucleotide polymorphism; Chr: chromosome; A1:effect allele; A2:other allele. | | | | | | | | | |

| **Table S6. Characteristics of women who were followed up for 40 years, with or without a diagnosis of PCOS.** | | | |
| --- | --- | --- | --- |
|  | **Women with PCOS (N=4079)** | **Women without PCOS (N=251,401)** | **Total (N=255,480)** |
| **Year of Birth (N, %)** |  |  |  |
| 1973 | 582 (14.27) | 45,641 (18.15) | 46,223 (18.09) |
| 1974 | 654 (16.03) | 45,666 (18.16) | 46,320 (18.13) |
| 1975 | 629 (15.42) | 42,979 (17.1) | 43,608 (17.07) |
| 1976 | 696 (17.06) | 40,393 (16.07) | 41,089 (16.08) |
| 1977 | 745 (18.26) | 39,005 (15.52) | 39,750 (15.56) |
| 1978 | 773 (18.95) | 37,717 (15.00) | 38,490 (15.07) |
| **Obesity (N, %)** | 966 (23.68) | 15,524 (6.17) | 16,490 (6.45) |
| **MS (N, %)** | 15 (0.37) | 1200 (0.48) | 1215 (0.48) |
| **Age at MS diagnosis (mean ± SD)** | 34.12 ± 6.45 | 32.98 ± 5.59 | 33.00 ± 5.60 |
| PCOS: polycystic ovary syndrome; MS: multiple sclerosis; SD: standard deviation. | | | |

| **Table S7. Causal associations between genetically predicted PCOS, SHBG, testosterone and risk of MS.** | | | | |
| --- | --- | --- | --- | --- |
| **Exposure** | **Method** | **# IV** | **OR (95% CI)** | **P** |
| **PCOS** | Inverse-variance weighted | 13 | 0.90 (0.80-1.01) | 0.07 |
|  | MR-Egger |  | 0.92 (0.52-1.61) | 0.76 |
|  | MR-Egger intercept |  |  | 0.95 |
|  | Weighted median |  | 0.89 (0.79-1.01) | 0.08 |
|  | Inverse-variance weighted (remove palindormic SNPs) | 13 | 0.90 (0.80-1.01) | 0.07 |
| **PCOS (remove obesity-related pleiotropic SNPs)** | Inverse-variance weighted | 9 | 0.97 (0.88-1.08) | 0.62 |
|  | MR-Egger |  | 0.74 (0.43-1.26) | 0.30 |
|  | MR-Egger intercept |  |  | 0.33 |
|  | Weighted median |  | 0.99 (0.86-1.14) | 0.90 |
|  | Inverse-variance weighted (remove palindormic SNPs) | 9 | 0.97 (0.88-1.08) | 0.62 |
| **SHBG** | Inverse-variance weighted | 168 | 1.23 (0.90-1.68) | 0.19 |
|  | MR-Egger |  | 0.94 (0.56-1.58) | 0.83 |
|  | MR-Egger intercept |  |  | 0.21 |
|  | Weighted median |  | 0.83 (0.61-1.14) | 0.26 |
|  | Inverse-variance weighted (remove palindormic SNPs) | 141 | 1.02 (0.81-1.29) | 0.87 |
| **SHBG_adj_BMI** | Inverse-variance weighted | 262 | 1.23 (0.94-1.60) | 0.13 |
|  | MR-Egger |  | 0.98 (0.63-1.55) | 0.95 |
|  | MR-Egger intercept |  |  | 0.24 |
|  | Weighted median |  | 0.81 (0.59-1.11) | 0.19 |
|  | Inverse-variance weighted (remove palindormic SNPs) | 221 | 1.04 (0.83-1.30) | 0.74 |
| **Total testosterone** | Inverse-variance weighted | 183 | 0.89 (0.77-1.04) | 0.13 |
|  | MR-Egger |  | 0.71 (0.51-1.00) | 0.05 |
|  | MR-Egger intercept |  |  | 0.15 |
|  | Weighted median |  | 0.93 (0.78-1.10) | 0.41 |
|  | Inverse-variance weighted (remove palindormic SNPs) | 154 | 0.88 (0.74-1.04) | 0.13 |
| **Bioavailable testosterone** | Inverse-variance weighted | 123 | 0.99 (0.82-1.19) | 0.92 |
|  | MR-Egger |  | 1.00 (0.67-1.49) | 0.99 |
|  | MR-Egger intercept |  |  | 0.94 |
|  | Weighted median |  | 1.26 (0.99-1.60) | 0.06 |
|  | Inverse-variance weighted (remove palindormic SNPs) | 108 | 1.02 (0.83-1.25) | 0.84 |
| PCOS: polycystic ovary syndrome; SHBG: sex hormone-binding globulin; SHBGadjBMI: sex hormone-binding globulin adjusted for BMI; MS: multiple sclerosis; MR: mendelian randomization; IV: instrumental variables; OR: odds ratio; CI: confidence interval. | | | | |

| **Table S8. Reverse causal associations between genetically predicted risk of MS and risk of PCOS, levels of SHBG and testosterone.** | | | | |
| --- | --- | --- | --- | --- |
| **Outcome** | **Method** | **# IV** | **OR (95% CI)** | **P** |
| **PCOS** | Inverse-variance weighted | 176 | 0.99 (0.93-1.04) | 0.65 |
|  | MR-Egger |  | 1.04 (0.86-1.25) | 0.70 |
|  | MR-Egger intercept |  |  | 0.59 |
|  | Weighted median |  | 0.99 (0.91-1.08) | 0.88 |
| **SHBG** | Inverse-variance weighted | 176 | 1.01 (1.00-1.01) | 0.03 |
|  | MR-Egger |  | 1.00 (0.98-1.02) | 0.93 |
|  | MR-Egger intercept |  |  | 0.56 |
|  | Weighted median |  | 1.00 (1.00-1.01) | 0.12 |
| **SHBG_adj_BMI** | Inverse-variance weighted | 176 | 1.01 (1.00-1.01) | 0.04 |
|  | MR-Egger |  | 1.00 (0.99-1.02) | 0.75 |
|  | MR-Egger intercept |  |  | 0.76 |
|  | Weighted median |  | 1.00 (1.00-1.01) | 0.02 |
| **Total testosterone** | Inverse-variance weighted | 176 | 1.01 (1.00-1.02) | 0.15 |
|  | MR-Egger |  | 1.00 (0.96-1.03) | 0.88 |
|  | MR-Egger intercept |  |  | 0.54 |
|  | Weighted median |  | 1.00 (0.99-1.01) | 0.41 |
| **Bioavailable testosterone** | Inverse-variance weighted | 176 | 1.00 (0.99-1.01) | 0.67 |
|  | MR-Egger |  | 1.00 (0.97-1.02) | 0.78 |
|  | MR-Egger intercept |  |  | 0.87 |
|  | Weighted median |  | 1.00 (0.99-1.00) | 0.41 |
| PCOS: polycystic ovary syndrome; SHBG: sex hormone-binding globulin; SHBG_adj_BMI: sex hormone-binding globulin adjusted for BMI; MS: multiple sclerosis; MR: mendelian randomization; IV:instrumental variables; MS: multiple sclerosis; OR: odds ratio; CI: confidence intervals. | | | | |

| **Table S9. Power calculation for Mendelian randomization study using assumed ORs .** | | | | | | | |
| --- | --- | --- | --- | --- | --- | --- | --- |
| **Exposure** | **Variance explained by index SNPs** | **Sample size (% cases)** | **F-statistic** | **Power panel to detect OR** | | | **OR under 80% power** |
|  |  |  |  | **1.05/0.95** | **1.10/0.91** | **1.15/0.87** |  |
| PCOS | 0.005 | 41505 (35.66) | 209.57 | 0.06 | 0.10 | 0.17 | 1.47/0.65 |
| SHBG | 0.095 | 41505 (35.66) | 4357.88 | 0.32 | 0.83 | 0.99 | 1.10/0.91 |
| SHBG_adj_BMI | 0.145 | 41505 (35.66) | 7039.86 | 0.45 | 0.95 | 1.00 | 1.08/0.93 |
| Total testosterone | 0.063 | 41505 (35.66) | 2791.62 | 0.23 | 0.66 | 0.94 | 1.12/0.89 |
| Bioavailable testosterone | 0.043 | 41505 (35.66) | 1865.91 | 0.17 | 0.50 | 0.82 | 1.15/0.87 |
| PCOS: polycystic ovary syndrome; SHBG: sex hormone-binding globulin; SHBG_adj_BMI: sex hormone-binding globulin adjusted for BMI; SNP: single nucleotide polymorphism; OR: odds ratio; IVW method: Inverse-variance weighted method. | | | | | | | |

| **Table S10. Local genetic covariance between PCOS, SHBG, testosterone and MS.** | | | | | |
| --- | --- | --- | --- | --- | --- |
| **Chr** | **Start** | **End** | **Covariance** | **Var** | **P** |
| **PCOS & MS** | | | | | |
| 1 | 2321099 | 3065568 | -0.0016 | 7.41×10^-8^ | 5.85×10^-9^ |
| 19 | 17944900 | 18506666 | -0.0015 | - | 9.64×10^-11^ |
| **SHBG & MS** | | | | | |
| 9 | 86181461 | 87571563 | 0.0014 | - | 3.00×10^-11^ |
| 16 | 10431221 | 11493266 | 0.0010 | 4.71×10^-8^ | 6.76×10^-6^ |
| 17 | 44863413 | 45874715 | 0.0018 | 9.44×10^-8^ | 1.13×10^-8^ |
| 19 | 17944900 | 18506666 | 0.0013 | 4.17×10^-8^ | 6.43×10^-10^ |
| **SHBG_adj_BMI & MS** | | | | | |
| 9 | 86181461 | 87571563 | 0.0014 | 4.79×10^-8^ | 5.36×10^-10^ |
| 16 | 10431221 | 11493266 | 0.0012 | 5.48×10^-8^ | 3.69×10^-7^ |
| 17 | 44863413 | 45874715 | 0.0022 | 1.29×10^-7^ | 1.13×10^-9^ |
| 19 | 17944900 | 18506666 | 0.0011 | 3.54×10^-8^ | 1.00×10^-8^ |
| 22 | 27654838 | 29454477 | 0.0009 | 3.97×10^-8^ | 1.54×10^-5^ |
| **Total testosterone & MS** | | | | | |
| 4 | 108469756 | 109979131 | 0.0010 | 3.39×10^-8^ | 1.58×10^-7^ |
| 8 | 24457122 | 25461315 | -0.0004 | 6.07×10^-9^ | 4.14×10^-6^ |
| 12 | 106156001 | 107521770 | -0.0006 | 1.22×10^-8^ | 1.75×10^-7^ |
| **Bioavailable testosterone & MS** | | | | | |
| 8 | 24457122 | 25461315 | -0.0004 | 6.54×10^-9^ | 4.92×10^-6^ |
| 9 | 4591655 | 5273194 | -0.0004 | 6.90×10^-9^ | 2.09×10^-5^ |
| 9 | 86181461 | 87571563 | -0.0008 | 2.54×10^-8^ | 2.80×10^-7^ |
| 16 | 10431221 | 11493266 | -0.0007 | 2.39×10^-8^ | 1.14×10^-5^ |
| 17 | 44863413 | 45874715 | -0.0014 | 5.11×10^-8^ | 7.78×10^-10^ |
| 20 | 39455778 | 40584825 | 0.0004 | 9.24×10^-9^ | 1.20×10^-10^ |
| Chr: chromosome; Start: the start position; End: the end position; Var: the variance of the estimation of local genetic covariance; SHBG_adj_BMI: sex hormone-binding globulin adjusted for BMI; SHBG: sex hormone-binding globulin; PCOS: polycystic ovary syndrome; MS: multiple sclerosis. | | | | | |

| **Table S11. Cross-trait meta-analysis between SHBG, testosterone and MS (PCPASSOC < 5×10-8, Psingle trair< 1×10-5).*** | | | | | | | | | | |
| --- | --- | --- | --- | --- | --- | --- | --- | --- | --- | --- |
| **SNP** | **Chr** | **bp** | **A1** | **A2** | **Beta** | | **P_trait_** | **P_MS_** | **P_CPASSOC_** | **Gene symbol** |
|  |  |  |  |  | **Trait** | **MS** |  |  |  |  |
| **SHBG & MS** | | | | | | | | | | |
| rs2289746 | 3 | 105455955 | T | C | -0.0082 | -0.0852 | 2.90E-07 | 1.48E-06 | 1.71E-10 | CBLB |
| rs9392863 | 6 | 7195009 | G | A | 0.0080 | 0.0937 | 6.30E-07 | 1.35E-06 | 6.77E-09 | RREB1 |
| rs989134^#^ | 6 | 26336224 | A | C | -0.0070 | 0.0753 | 1.90E-06 | 5.94E-06 | 1.40E-08 | HIST1H2BI HIST1H3G HIST1H4H |
| rs17513809 | 6 | 139829917 | G | A | -0.0088 | -0.1056 | 4.60E-07 | 4.52E-06 | 5.07E-09 |  |
| rs56232455 | 11 | 321235 | G | A | -0.0084 | -0.1584 | 6.70E-09 | 1.78E-08 | 4.51E-12 | IFITM1 IFITM2 IFITM3 |
| rs3922 | 11 | 118765600 | A | G | 0.0068 | 0.0813 | 2.00E-06 | 1.26E-06 | 1.76E-08 | BCL9L CXCR5 |
| rs56191111 | 17 | 45571505 | A | C | -0.0112 | -0.2331 | 1.40E-09 | 3.30E-07 | 3.90E-12 | EFCAB13 ITGB3 KPNB1 LOC102724508 MRPL45P2 NPEPPS TBKBP1 TBX21 |
| rs7350906 | 17 | 45588112 | G | A | -0.0186 | -0.1053 | 4.10E-40 | 6.56E-10 | 1.19E-50 | EFCAB13 ITGB3 KPNB1 LOC102724508 MRPL45P2 NPEPPS TBKBP1 TBX21 |
| rs9910408 | 17 | 45802874 | A | G | -0.0079 | -0.1017 | 2.70E-08 | 4.72E-06 | 3.23E-10 |  |
| rs72648871 | 17 | 45816108 | C | T | 0.0160 | 0.5037 | 2.50E-07 | 7.88E-08 | 1.63E-10 | TBX21 |
| rs885683 | 19 | 18244690 | G | A | 0.0129 | 0.1085 | 3.90E-16 | 2.02E-08 | 3.28E-20 | IFI30IL12RB1KIAA1683LOC729966MAST3MPV17L2PDE4CPIK3R2RAB3A |
| rs11086102 | 19 | 18398628 | G | C | -0.0087 | -0.0839 | 4.40E-10 | 9.78E-07 | 4.20E-12 | JUND KIAA1683 LOC729966 MIR3188 PDE4C |
| **SHBG_adj_BMI & MS** | | | | | | | | | | |
| rs2289746 | 3 | 105455955 | T | C | -0.0083 | -0.0852 | 2.60E-09 | 1.48E-06 | 6.93E-13 | CBLB |
| rs9392863 | 6 | 7195009 | G | A | 0.0072 | 0.0937 | 1.20E-06 | 1.35E-06 | 5.66E-09 | RREB1 |
| rs56232455 | 11 | 321235 | G | A | -0.0075 | -0.1584 | 9.90E-10 | 1.78E-08 | 5.36E-12 | IFITM1 IFITM2 IFITM3 |
| rs12365699 | 11 | 118743286 | G | A | 0.0068 | 0.1438 | 2.60E-06 | 3.15E-10 | 6.46E-10 | DDX6 |
| rs1706477 | 12 | 123913697 | T | G | 0.0059 | 0.1124 | 2.40E-07 | 1.22E-06 | 3.79E-08 | C12orf65 CDK2AP1 LOC100507091 MIR8072 MPHOSPH9 PITPNM2 RILPL2 SBNO1 SETD8 |
| rs8079821 | 17 | 45611704 | T | C | -0.0195 | -0.1062 | 1.80E-61 | 5.25E-10 | 1.92E-68 | EFCAB13ITGB3KPNB1LOC102724508MRPL45P2NPEPPSTBKBP1TBX21 |
| rs8073834 | 17 | 45698483 | G | A | -0.0098 | -0.1860 | 1.50E-10 | 1.44E-06 | 3.42E-12 | EFCAB13 ITGB3 KPNB1 LOC102724508 MRPL45P2 NPEPPS TBKBP1 |
| rs9910408 | 17 | 45802874 | A | G | -0.0100 | -0.1017 | 7.30E-17 | 4.72E-06 | 2.65E-18 |  |
| rs72648871 | 17 | 45816108 | C | T | 0.0187 | 0.5037 | 1.30E-12 | 7.88E-08 | 3.05E-16 | KPNB1 TBKBP1 TBX21 |
| rs72999466 | 19 | 18248499 | C | T | 0.0103 | 0.1089 | 9.60E-14 | 1.78E-08 | 1.32E-16 | IFI30 IL12RB1 KIAA1683 LOC729966 MAST3 MPV17L2 PDE4C PIK3R2 RAB3A |
| rs12610373 | 19 | 18398284 | A | T | -0.0074 | -0.0844 | 7.90E-10 | 8.53E-07 | 3.81E-11 | JUND KIAA1683 LOC729966 MIR3188 PDE4C |
| rs16986308 | 22 | 28717032 | G | A | 0.0103 | 0.1208 | 2.10E-07 | 1.97E-06 | 9.09E-10 | CCDC117CHEK2HSCBMIR5739TTC28TTC28-AS1XBP1 |
| **Total testosterone & MS** | | | | | | | | | | |
| rs4245930 | 4 | 109038654 | G | A | 0.0254 | 0.0844 | 1.10E-19 | 4.93E-07 | 4.37E-23 | CYP2U1 HADH LEF1 LEF1-AS1 |
| rs853158 | 5 | 142605172 | T | C | 0.0160 | 0.0825 | 2.20E-07 | 6.69E-06 | 6.18E-09 | ARHGAP26 |
| rs72980770 | 6 | 138234289 | C | T | -0.0196 | 0.1163 | 5.20E-07 | 1.25E-06 | 4.12E-09 | LOC100130476 TNFAIP3 |
| rs7918084 | 10 | 94429467 | C | T | -0.0270 | 0.0917 | 1.30E-21 | 4.50E-08 | 9.02E-28 | HHEX IDE KIF11 |
| rs1790120 | 12 | 123613565 | A | C | 0.0179 | 0.1074 | 9.80E-08 | 3.98E-08 | 1.53E-09 | ABCB9 ARL6IP4 C12orf65 CDK2AP1 LOC100507091 MIR4304 MIR8072 MPHOSPH9 OGFOD2 PITPNM2 RILPL2 SBNO1 SETD8 |
| rs1469024 | 19 | 18384544 | C | T | -0.0281 | -0.0859 | 1.40E-22 | 5.10E-07 | 1.91E-27 | IFI30JUNDKIAA1683LOC729966MIR3188MPV17L2PDE4CRAB3A |
| rs11671638 | 19 | 47619560 | G | A | 0.0164 | 0.0819 | 6.70E-08 | 6.58E-06 | 1.61E-09 | BBC3 MIR3190 MIR3191 SAE1 ZC3H4 |
| rs884171 | 19 | 47716347 | T | A | -0.0168 | -0.1036 | 5.30E-09 | 7.83E-07 | 3.06E-10 | SAE1 |
| **Bioavailable testosterone & MS** | | | | | | | | | | |
| rs7668764 | 4 | 109067898 | G | T | 0.0139 | 0.0861 | 5.50E-08 | 3.31E-07 | 6.60E-10 | LEF1 LEF1-AS1 |
| rs10048173 | 17 | 45592266 | A | G | 0.0242 | -0.1056 | 1.70E-22 | 6.13E-10 | 1.10E-27 | EFCAB13 ITGB3 KPNB1 LOC102724508 MRPL45P2 NPEPPS TBKBP1 TBX21 |
| rs9910408 | 17 | 45802874 | A | G | 0.0144 | -0.1017 | 1.10E-07 | 4.72E-06 | 3.83E-10 |  |
| * The MHC region was excluded from this analysis; # A novel pleiotropic SNP, which was defined as a significant pleiotropic SNP neither reached genome-wide significance in a single trait (5×10^-8^ < P_sex hormone trait_ < 1×10^-5^, 5×10^-8^ < P_MS_ < 1×10^-5^, and P_CPASSO_C < 5×10^-8^) nor in linkage-disequilibrium (LD) with previously identified sex hormones- or MS-associated SNPs; SNP: single nucleotide polymorphism; Chr: chromosome; bp: base pair; A1:effect allele; A2:other allele; SHBG: sex hormone-binding globulin; SHBG_adj_BMI: sex hormone-binding globulin adjusted for BMI; MS: multiple sclerosis. | | | | | | | | | | |

| **Table S12. List of 95% credible set SNPs in each locus identified from fine-mapping analysis (r2 threshold=0.6).*** | | |
| --- | --- | --- |
| **Index SNP** | **No. of credible set SNPs** | **List of credible set SNPs (posterior probability in brackets)** |
| **SHBG & MS** | | |
| rs2289746 | 14 | rs2289746 (0.35), rs6777124 (0.14), rs7615115 (0.07), rs35903551 (0.07), rs7646159 (0.06), rs726443 (0.05), rs2301046 (0.03), rs2301045 (0.03), rs2301044 (0.03), rs2034157 (0.03), rs1522287 (0.03), rs7645026 (0.02), rs7612189 (0.02), rs11708360 (0.02) |
| rs9392863 | 2 | rs9392863 (0.81), rs4960291 (0.19) |
| rs989134 | 29 | rs989134 (0.13), rs9366651 (0.13), rs9358919 (0.06), rs6916289 (0.05), rs4412192 (0.05), rs9358915 (0.05), rs9358917 (0.05), rs9393693 (0.05), rs9393694 (0.05), rs9379838 (0.05), rs7761298 (0.05), rs9366649 (0.04), rs6456712 (0.02), rs9393692 (0.02), rs1125000 (0.02), rs9357004 (0.01), rs6939589 (0.01), rs7748935 (0.01), rs2393593 (0.01), rs1124999 (0.01), rs9379843 (0.01), rs3999544 (0.01), rs34916901 (0.01), rs2142703 (0.01), rs12526215 (0.01), rs7747133 (0.01), rs6940237 (0.01), rs6931391 (0.01), rs7771202 (0.01) |
| rs17513809 | 5 | rs17513809 (0.40), rs60562771 (0.27), rs72976941 (0.14), rs72976934 (0.12), rs72976928 (0.07) |
| rs56232455 | 2 | rs56232455 (0.57), rs34481144 (0.38) |
| rs3922 | 4 | rs7104819 (0.44), rs581063 (0.25), rs11217081 (0.21), rs11217083 (0.09) |
| rs56191111 | 1 | rs62076510 (0.95) |
| rs7350906 | 30 | rs7350906 (0.09), rs7222851 (0.09), rs8067286 (0.08), rs7219173 (0.06), rs10048173 (0.06), rs8079821 (0.05), rs62073965 (0.05), rs4264433 (0.05), rs8077485 (0.04), rs10445374 (0.03), rs62073966 (0.03), rs7210472 (0.03), rs201496912 (0.03), rs34609599 (0.03), rs8065099 (0.03), rs62074014 (0.03), rs8077106 (0.02), rs8072100 (0.02), rs7220935 (0.01), rs12603290 (0.01), rs8081717 (0.01), rs12150231 (0.01), rs11870935 (0.01), rs9905583 (0.01), rs4580230 (0.01), rs8065669 (0.01), rs11650372 (0.01), rs9897957 (0.01), rs7210738 (0.01), rs56325564 (0.01) |
| rs9910408 | 1 | rs9910408 (1.00) |
| rs72648871 | 1 | rs72648871 (1.00) |
| rs885683 | 15 | rs885683 (0.16), rs11666906 (0.15), rs4808751 (0.10), rs56252442 (0.09), rs72999466 (0.08), rs72999449 (0.06), rs273492 (0.04), rs11668601 (0.04), rs11666281 (0.04), rs4808745 (0.04), rs4808114 (0.03), rs273488 (0.03), rs56345159 (0.03), rs72999447 (0.03), rs56338130 (0.02) |
| rs11086102 | 16 | rs11086102 (0.10), rs12610373 (0.10), rs12980403 (0.10), rs12973608 (0.09), rs7256111 (0.08), rs7247222 (0.08), rs11666808 (0.06), rs1469024 (0.05), rs8109944 (0.05), rs8110070 (0.04), rs10416600 (0.04), rs4808779 (0.04), rs8113367 (0.04), rs4531856 (0.04), rs1075403 (0.03), rs12608504 (0.03) |
| **SHBG_adj_BMI & MS** | | |
| rs2289746 | 13 | rs2289746 (0.46), rs7615115 (0.12), rs6777124 (0.08), rs2301046 (0.04), rs35903551 (0.04), rs2301045 (0.03), rs7646159 (0.03), rs2301044 (0.03), rs2034157 (0.03), rs1522287 (0.03), rs726443 (0.03), rs7645026 (0.02), rs7612189 (0.02) |
| rs9392863 | 2 | rs9392863 (0.81), rs4960291 (0.19) |
| rs56232455 | 2 | rs56232455 (0.74), rs34481144 (0.23) |
| rs12365699 | 49 | rs12365699 (0.28), rs4936444 (0.05), rs7117313 (0.04), rs7125333 (0.03), rs7481819 (0.03), rs10790275 (0.03), rs7481797 (0.03), rs10892296 (0.03), rs11217046 (0.02), rs7123726 (0.02), rs11217058 (0.02), rs73005502 (0.02), rs11217045 (0.02), rs11217060 (0.02), rs4936441 (0.02), rs11217066 (0.02), rs10892299 (0.02), rs10790269 (0.02), rs7122669 (0.01), rs4936443 (0.01), rs7951740 (0.01), rs7117261 (0.01), rs4938572 (0.01), rs7119044 (0.01), rs11217074 (0.01), rs10790268 (0.01), rs4938573 (0.01), rs11217044 (0.01), rs874621 (0.01), rs10892292 (0.01), rs11217042 (0.01), rs17122453 (0.01), rs10892291 (0.01), rs10892290 (0.01), rs57494551 (0.01), rs7942535 (0.01), rs11217041 (0.01), rs73005426 (0.01), rs11217040 (0.01), rs715412 (0.01), rs28409215 (0.01), rs73005423 (0.01), rs3889239 (0.01), rs11217009 (0.01), rs2004781 (0.01), rs2077579 (0.01), rs1048024 (0.01), rs56758835 (0.01), rs11826521 (0.00) |
| rs1706477 | 5 | rs1706477(0.53), rs28713448(0.19), rs28520455(0.12), rs28404303(0.09), rs1662(0.07) |
| rs8079821 | 27 | rs8079821 (0.10), rs4264433 (0.09), rs7219173 (0.08), rs62073965 (0.07), rs7210472 (0.06), rs10445374 (0.05), rs7222851 (0.04), rs62073966 (0.04), rs62074014 (0.04), rs7350906 (0.03), rs8067286 (0.03), rs34609599 (0.03), rs8065099 (0.03), rs8072100 (0.03), rs10048173 (0.03), rs8077485 (0.03), rs201496912 (0.02), rs8077106 (0.02), rs7220935 (0.02), rs8081717 (0.02), rs12150231 (0.02), rs12603290 (0.02), rs11650372 (0.01), rs8065669 (0.01), rs8065437 (0.01), rs11870935 (0.01), rs9905583 (0.01) |
| rs8073834 | 1 | rs62073987 (1.00) |
| rs9910408 | 1 | rs9910408 (1.00) |
| rs72648871 | 1 | rs72648871 (1.00) |
| rs72999466 | 17 | rs72999466 (0.11), rs4808751 (0.11), rs885683 (0.10), rs273510 (0.10), rs56252442 (0.09), rs11666906 (0.09), rs273507 (0.06), rs72999449 (0.05), rs4808745 (0.04), rs4808114 (0.04), rs11668601 (0.04), rs11666281 (0.04), rs273492 (0.03), rs273488 (0.02), rs72999447 (0.02), rs11671389 (0.01), rs56345159 (0.01) |
| rs12610373 | 18 | rs12610373 (0.11), rs11086102 (0.10), rs12980403 (0.09), rs12973608 (0.09), rs7256111 (0.07), rs7247222 (0.07), rs8109944 (0.06), rs8110070 (0.04), rs8113367 (0.04), rs1469024 (0.04), rs10416600 (0.04), rs4531856 (0.04), rs1075403 (0.04), rs12608504 (0.03), rs4808779 (0.03), rs11666808 (0.02), rs12610691 (0.02), rs11086103 (0.02) |
| rs16986308 | 36 | rs16986308 (0.14), rs16986398 (0.12), rs12170637 (0.05), rs134506 (0.05), rs4583569 (0.04), rs2144922 (0.04), rs2013784 (0.04), rs5752754 (0.04), rs6005809 (0.04), rs2881496 (0.04), rs134484 (0.04), rs134481 (0.03), rs9625433 (0.03), rs9625438 (0.03), rs134490 (0.02), rs134488 (0.02), rs1297607 (0.02), rs134483 (0.02), rs134495 (0.02), rs134492 (0.01), rs12166475 (0.01), rs134524 (0.01), rs9625447 (0.01), rs73430118 (0.01), rs9620779 (0.01), rs134547 (0.01), rs9625441 (0.01), rs9625440 (0.01), rs7289625 (0.01), rs9625437 (0.01), rs10212083 (0.00), rs134514 (0.00), rs12167532 (0.00), rs9625498 (0.00), rs56391942 (0.00), rs17487463 (0.00) |
| **Total testosterone & MS** | | |
| rs4245930 | 2 | rs4245930 (0.83), rs7668764 (0.13) |
| rs853158 | 8 | rs853158 (0.33), rs258825 (0.32), rs33384 (0.13), rs258796 (0.10), rs244471 (0.02), rs244472 (0.02), rs244474 (0.02), rs244470 (0.02) |
| rs72980770 | 5 | rs72980770 (0.33), rs17780429 (0.3), rs72980776 (0.21), rs55771473 (0.07), rs17779870 (0.06) |
| rs7918084 | 2 | rs7918084 (0.80), rs2497318 (0.18) |
| rs1790120 | 143 | rs1790120 (0.02), rs1727311 (0.02), rs1716174 (0.02), rs1716173 (0.02), rs1630820 (0.02), rs1727309 (0.02), rs1790087 (0.01), rs1727310 (0.01), rs1716158 (0.01), rs883563 (0.01), rs1716156 (0.01), rs1790125 (0.01), rs1716159 (0.01), rs1716157 (0.01), rs10772995 (0.01), rs641760 (0.01), rs1790105 (0.01), rs940904 (0.01), rs585522 (0.01), rs10846478 (0.01), rs1615694 (0.01), rs2049114 (0.01), rs2851436 (0.01), rs1727306 (0.01), rs1716175 (0.01), rs937564 (0.01), rs883263 (0.01), rs1727317 (0.01), rs1790123 (0.01), rs520088 (0.01), rs655293 (0.01), rs1463877 (0.01), rs1716169 (0.01), rs1790115 (0.01), rs2510885 (0.01), rs1616181 (0.01), rs1790119 (0.01), rs1727318 (0.01), rs1616131 (0.01), rs1716170 (0.01), rs1716171 (0.01), rs941306 (0.01), rs1790116 (0.01), rs1727294 (0.01), rs2851441 (0.01), rs949142 (0.01), rs2695480 (0.01), rs941305 (0.01), rs1727334 (0.01), rs2695482 (0.01), rs1727322 (0.01), rs1727324 (0.01), rs1619561 (0.01), rs1790090 (0.01), rs7975763 (0.01), rs884548 (0.01), rs1569068 (0.01), rs1727313 (0.01), rs1106240 (0.01), rs2851438 (0.01), rs10744149 (0.01), rs1617156 (0.01), rs10846486 (0.01), rs1630693 (0.01), rs1106241 (0.01), rs2695476 (0.01), rs2695479 (0.01), rs2695478 (0.01), rs1626899 (0.01), rs1790097 (0.01), rs2682433 (0.01), rs1716172 (0.01), rs12829456 (0.01), rs2851435 (0.01), rs1051431 (0.00), rs1716165 (0.00), rs1621194 (0.00), rs2851450 (0.00), rs1716167 (0.00), rs2682431 (0.00), rs1618679 (0.00), rs12817892 (0.00), rs1727300 (0.00), rs2851451 (0.00), rs7299943 (0.00), rs1727296 (0.00), rs1716164 (0.00), rs1617434 (0.00), rs1630905 (0.00), rs1727314 (0.00), rs1790102 (0.00), rs2695481 (0.00), rs1790101 (0.00), rs2682429 (0.00), rs1716168 (0.00), rs1790130 (0.00), rs1716160 (0.00), rs2682430 (0.00), rs1727320 (0.00), rs1727325 (0.00), rs2950537 (0.00), rs1716176 (0.00), rs1402274 (0.00), rs1790095 (0.00), rs2947099 (0.00), rs1716177 (0.00), rs3018098 (0.00), rs1627724 (0.00), rs1727315 (0.00), rs10744148 (0.00), rs1716182 (0.00), rs1790108 (0.00), rs1790126 (0.00), rs1790131 (0.00), rs1727323 (0.00), rs1716185 (0.00), rs71444568 (0.00), rs4372492 (0.00), rs10744147 (0.00), rs1790124 (0.00), rs1790109 (0.00), rs1790100 (0.00), rs1047158 (0.00), rs1790098 (0.00), rs1727290 (0.00), rs10772996 (0.00), rs2682434 (0.00), rs11057192 (0.00), rs1260294 (0.00), rs1270594 (0.00), rs58809476 (0.00), rs2682426 (0.00), rs12818067 (0.00), rs1727316 (0.00), rs7132277 (0.00), rs12811109 (0.00), rs1716183 (0.00), rs34341465 (0.00), rs6633 (0.00), rs4148856 (0.00), rs12820906 (0.00), rs1879380 (0.00), rs1790133 (0.00) |
| rs1469024 | 14 | rs1469024 (0.13), rs7247222 (0.10), rs7256111 (0.09), rs12980403 (0.08), rs1075403 (0.07), rs8109944 (0.07), rs10416600 (0.06), rs12608504 (0.06), rs11086102 (0.06), rs12610373 (0.06), rs8113367 (0.06), rs8110070 (0.06), rs4531856 (0.05), rs12973608 (0.05) |
| rs11671638 | 24 | rs11671638 (0.14), rs10403089 (0.14), rs11671656 (0.07), rs55731973 (0.05), rs12609406 (0.05), rs4804025 (0.05), rs4804024 (0.04), rs2241388 (0.04), rs4804023 (0.04), rs2303107 (0.03), rs7250850 (0.03), rs10408163 (0.03), rs7248181 (0.03), rs11083860 (0.03), rs8101149 (0.03), rs62136856 (0.03), rs4804026 (0.02), rs3810291 (0.02), rs7255995 (0.02), rs7251104 (0.01), rs11667244 (0.01), rs3745619 (0.01), rs6509311 (0.01), rs2303108 (0.01) |
| rs884171 | 6 | rs884171 (0.26), rs6509314 (0.26), rs307896 (0.20), rs2862235 (0.18), rs461709 (0.05), rs2909902 (0.04) |
| **Bioavailable testosterone & MS** | | |
| rs7668764 | 24 | rs7668764 (0.24), rs4245930 (0.22), rs922163 (0.07), rs922165 (0.07), rs4956037 (0.06), rs922164 (0.06), rs4245929 (0.05), rs4624731 (0.03), rs9992327 (0.02), rs62310705 (0.02), rs10011173 (0.01), rs956237 (0.01), rs7694643 (0.01), rs4423942 (0.01), rs6838919 (0.01), rs28617637 (0.01), rs7654502 (0.01), rs9992763 (0.00), rs61399930 (0.00), rs7666005 (0.00), rs4458527 (0.00), rs9999061 (0.00), rs6533351 (0.00), rs10025623 (0.00) |
| rs10048173 | 74 | rs10048173 (0.02), rs7222851 (0.02), rs10445374 (0.02), rs7223029 (0.02), rs7350906 (0.02), rs56325564 (0.02), rs11656855 (0.02), rs4793913 (0.02), rs11079784 (0.02), rs8067286 (0.02), rs4289035 (0.02), rs7219173 (0.02), rs8079821 (0.02), rs9897957 (0.02), rs7207542 (0.02), rs4793908 (0.02), rs3760370 (0.02), rs7220935 (0.02), rs8077485 (0.02), rs62074014 (0.02), rs4264433 (0.02), rs201496912 (0.02), rs8075411 (0.01), rs7210472 (0.01), rs4793978 (0.01), rs11871606 (0.01), rs10853090 (0.01), rs10432035 (0.01), rs7219303 (0.01), rs8078880 (0.01), rs4399567 (0.01), rs12943464 (0.01), rs8072100 (0.01), rs12603290 (0.01), rs3809868 (0.01), rs4794058 (0.01), rs73315878 (0.01), rs10163469 (0.01), rs12937560 (0.01), rs12150231 (0.01), rs4793842 (0.01), rs8077106 (0.01), rs4794001 (0.01), rs8081717 (0.01), rs11870935 (0.01), rs34609599 (0.01), rs8065099 (0.01), rs11079782 (0.01), rs4239162 (0.01), rs4239163 (0.01), rs55633793 (0.01), rs199538746 (0.01), rs7210738 (0.01), rs4794048 (0.01), rs9905922 (0.01), rs62073966 (0.01), rs145346039 (0.01), rs9905583 (0.01), rs4580230 (0.01), rs11650372 (0.01), rs2317826 (0.01), rs4092460 (0.01), rs62073965 (0.01), rs8072644 (0.01), rs9635762 (0.01), rs8082284 (0.01), rs9635763 (0.01), rs11079779 (0.01), rs35806769 (0.01), rs4794007 (0.01), rs8065990 (0.01), rs8065437 (0.01), rs55687600 (0.01), rs2935183 (0.01) |
| rs9910408 | 1 | rs9910408 (1.00) |
| *The MHC region was excluded from this analysis; SHBG: sex hormone-binding globulin; SHBGadjBMI: sex hormone-binding globulin adjusted for BMI; MS: multiple sclerosis. | | |

| **Table S13. Shared significant genes between SHBG, testosterone and MS from transcriptome-wide association studies using gene expressions across 49 GTEx tissues.*** | | | | | | | |
| --- | --- | --- | --- | --- | --- | --- | --- |
| **Chr** | **Gene** | **Tissue** | **P_trait_** | |  | **P_MS_** | |
| **SHBG & MS** | | | | | | | |
| 1 | GFI1 | Pituitary |  | 4.83E-10 |  |  | 2.31E-09 |
| 17 | KPNB1 | Brain_Caudate_basal_ganglia Brain_Hippocampus Brain_Hypothalamus Liver | < | 5.84E-19 |  | < | 2.52E-09 |
| 17 | NPEPPS | Adipose_Subcutaneous Adipose_Visceral_Omentum Adrenal_Gland Artery_Aorta Artery_Coronary Artery_Tibial Brain_Caudate_basal_ganglia Brain_Cerebellar_Hemisphere Brain_Cerebellum Brain_Cortex Brain_Hippocampus Brain_Hypothalamus Brain_Nucleus_accumbens_basal_ganglia Brain_Spinal_cord_cervical_c-1 Brain_Substantia_nigra Breast_Mammary_Tissue Cells_Cultured_fibroblasts Cells_EBV-transformed_lymphocytes Colon_Sigmoid Colon_Transverse Esophagus_Gastroesophageal_Junction Esophagus_Mucosa Esophagus_Muscularis Heart_Atrial_Appendage Heart_Left_Ventricle Lung Minor_Salivary_Gland Nerve_Tibial Pancreas Pituitary Prostate Small_Intestine_Terminal_Ileum Spleen Stomach Testis Thyroid Uterus Whole_Blood | < | 3.92E-13 |  | < | 7.30E-09 |
| 17 | TBKBP1 | Adipose_Subcutaneous Adipose_Visceral_Omentum Adrenal_Gland Artery_Aorta Artery_Coronary Artery_Tibial Brain_Caudate_basal_ganglia Brain_Cerebellum Brain_Cortex Brain_Frontal_Cortex_BA9 Breast_Mammary_Tissue Cells_Cultured_fibroblasts Cells_EBV-transformed_lymphocytes Colon_Sigmoid Colon_Transverse Esophagus_Gastroesophageal_Junction Esophagus_Mucosa Esophagus_Muscularis Heart_Atrial_Appendage Heart_Left_Ventricle Liver Lung Minor_Salivary_Gland Muscle_Skeletal Nerve_Tibial Ovary Pituitary Prostate Skin_Not_Sun_Exposed_Suprapubic Skin_Sun_Exposed_Lower_leg Small_Intestine_Terminal_Ileum Spleen Stomach Testis Thyroid Uterus Vagina Whole_Blood | < | 9.87E-25 |  | < | 1.46E-09 |
| 17 | AC025682.1 | Adipose_Subcutaneous Adipose_Visceral_Omentum Artery_Aorta Artery_Coronary Colon_Sigmoid Esophagus_Muscularis Heart_Left_Ventricle Minor_Salivary_Gland Testis | < | 5.58E-24 |  | < | 4.19E-09 |
| 17 | AC068234.2 | Adrenal_Gland |  | 9.88E-30 |  |  | 2.68E-09 |
| 19 | MAST3 | Brain_Cortex  Brain_Frontal_Cortex_BA9  Heart_Atrial_Appendage  Skin_Not_Sun_Exposed_Suprapubic  Skin_Sun_Exposed_Lower_leg | < | 4.48E-09 |  | < | 7.75E-09 |
| 19 | MPV17L2 | Adipose_Visceral_Omentum Artery_Aorta Artery_Tibial Brain_Cortex Brain_Frontal_Cortex_BA9 Brain_Nucleus_accumbens_basal_ganglia Brain_Spinal_cord_cervical_c-1 Brain_Substantia_nigra Breast_Mammary_Tissue Cells_Cultured_fibroblasts Cells_EBV-transformed_lymphocytes Colon_Sigmoid Colon_Transverse Esophagus_Gastroesophageal_Junction Esophagus_Mucosa Esophagus_Muscularis Lung Minor_Salivary_Gland Pancreas Prostate Skin_Not_Sun_Exposed_Suprapubic Skin_Sun_Exposed_Lower_leg Small_Intestine_Terminal_Ileum Testis Thyroid Vagina Whole_Blood | < | 7.60E-09 |  | < | 2.82E-10 |
| 19 | PDE4C | Artery_Aorta Artery_Coronary Brain_Amygdala Brain_Anterior_cingulate_cortex_BA24 Brain_Caudate_basal_ganglia Brain_Cerebellar_Hemisphere Brain_Cerebellum Brain_Cortex Brain_Nucleus_accumbens_basal_ganglia Brain_Putamen_basal_ganglia Brain_Spinal_cord_cervical_c-1 Brain_Substantia_nigra Colon_Sigmoid Heart_Atrial_Appendage Heart_Left_Ventricle Liver Muscle_Skeletal Nerve_Tibial Ovary Pituitary Spleen | < | 1.10E-09 |  | < | 1.04E-08 |
| **SHBG_adj_BMI & MS** | | | | | | | |
| 12 | OGFOD2 | Thyroid |  | 2.79E-10 |  |  | 3.84E-10 |
| 17 | KPNB1 | Brain_Caudate_basal_ganglia  Brain_Hippocampus  Brain_Hypothalamus  Liver | < | 1.45E-26 |  | < | 2.52E-09 |
| 17 | NPEPPS | Adipose_Subcutaneous Adipose_Visceral_Omentum Adrenal_Gland Artery_Aorta Artery_Coronary Artery_Tibial Brain_Caudate_basal_ganglia Brain_Cerebellar_Hemisphere Brain_Cerebellum Brain_Cortex Brain_Hippocampus Brain_Hypothalamus Brain_Nucleus_accumbens_basal_ganglia Brain_Spinal_cord_cervical_c-1 Brain_Substantia_nigra Breast_Mammary_Tissue Cells_Cultured_fibroblasts Cells_EBV-transformed_lymphocytes Colon_Sigmoid Colon_Transverse Esophagus_Gastroesophageal_Junction Esophagus_Mucosa Esophagus_Muscularis Heart_Atrial_Appendage Heart_Left_Ventricle Lung Minor_Salivary_Gland Nerve_Tibial Pancreas Pituitary Prostate Small_Intestine_Terminal_Ileum Spleen Stomach Testis Thyroid Uterus Whole_Blood | < | 2.35E-23 |  | < | 7.30E-09 |
| 17 | TBKBP1 | Adipose_Subcutaneous Adipose_Visceral_Omentum Adrenal_Gland Artery_Aorta Artery_Coronary Artery_Tibial Brain_Caudate_basal_ganglia Brain_Cerebellum Brain_Cortex Brain_Frontal_Cortex_BA9 Breast_Mammary_Tissue Cells_Cultured_fibroblasts Cells_EBV-transformed_lymphocytes Colon_Sigmoid Colon_Transverse Esophagus_Gastroesophageal_Junction Esophagus_Mucosa Esophagus_Muscularis Heart_Atrial_Appendage Heart_Left_Ventricle Liver Lung Minor_Salivary_Gland Muscle_Skeletal Nerve_Tibial Ovary Pituitary Prostate Skin_Not_Sun_Exposed_Suprapubic Skin_Sun_Exposed_Lower_leg Small_Intestine_Terminal_Ileum Spleen Stomach Testis Thyroid Uterus Vagina Whole_Blood | < | 2.69E-37 |  | < | 1.47E-09 |
| 17 | AC025682.1 | Adipose_Subcutaneous Adipose_Visceral_Omentum Artery_Aorta Artery_Coronary Colon_Sigmoid Esophagus_Muscularis Heart_Left_Ventricle Minor_Salivary_Gland Testis | < | 2.75E-39 |  | < | 4.19E-09 |
| 17 | AC068234.2 | Adrenal_Gland |  | 1.56E-42 |  |  | 2.68E-09 |
| 19 | MAST3 | Brain_Frontal_Cortex_BA9  Heart_Atrial_Appendage  Skin_Not_Sun_Exposed_Suprapubic  Skin_Sun_Exposed_Lower_leg | < | 1.40E-08 |  | < | 6.98E-09 |
| 19 | MPV17L2 | Artery_Tibial Brain_Cortex Brain_Frontal_Cortex_BA9 Brain_Nucleus_accumbens_basal_ganglia Brain_Spinal_cord_cervical_c-1 Brain_Substantia_nigra Cells_Cultured_fibroblasts Cells_EBV-transformed_lymphocytes Colon_Sigmoid Colon_Transverse Esophagus_Gastroesophageal_Junction Lung Minor_Salivary_Gland Pancreas Prostate Skin_Not_Sun_Exposed_Suprapubic Skin_Sun_Exposed_Lower_leg Small_Intestine_Terminal_Ileum Testis Thyroid Vagina Whole_Blood | < | 1.37E-09 |  | < | 8.23E-12 |
| 19 | PDE4C | Artery_Aorta Artery_Coronary Brain_Amygdala Brain_Anterior_cingulate_cortex_BA24 Brain_Caudate_basal_ganglia Brain_Cerebellar_Hemisphere Brain_Cerebellum Brain_Cortex Brain_Nucleus_accumbens_basal_ganglia Brain_Putamen_basal_ganglia Brain_Spinal_cord_cervical_c-1 Brain_Substantia_nigra Colon_Sigmoid Heart_Atrial_Appendage Heart_Left_Ventricle Liver Muscle_Skeletal Nerve_Tibial Ovary Spleen | < | 1.21E-08 |  | < | 1.04E-08 |
| **Total testosterone & MS** | | | | | | | |
| 1 | GFI1 | Pituitary |  | 9.22E-12 |  |  | 2.31E-09 |
| **Bioavailable testosterone & MS** | | | | | | | |
| 1 | GFI1 | Pituitary |  | 2.89E-15 |  |  | 2.31E-09 |
| 17 | KPNB1 | Brain_Caudate_basal_ganglia  Brain_Hippocampus  Liver | < | 8.76E-10 |  | < | 1.97E-10 |
| 17 | NPEPPS | Adipose_Subcutaneous Adipose_Visceral_Omentum Adrenal_Gland Artery_Aorta Artery_Coronary Artery_Tibial Brain_Caudate_basal_ganglia Brain_Cerebellar_Hemisphere Brain_Cortex Brain_Hippocampus Brain_Hypothalamus Brain_Nucleus_accumbens_basal_ganglia Brain_Spinal_cord_cervical_c-1 Brain_Substantia_nigra Breast_Mammary_Tissue Cells_Cultured_fibroblasts Cells_EBV-transformed_lymphocytes Colon_Sigmoid Colon_Transverse Esophagus_Gastroesophageal_Junction Esophagus_Mucosa Esophagus_Muscularis Heart_Atrial_Appendage Heart_Left_Ventricle Lung Minor_Salivary_Gland Nerve_Tibial Pancreas Pituitary Prostate Small_Intestine_Terminal_Ileum Spleen Stomach Testis Thyroid Uterus Whole_Blood | < | 6.91E-11 |  | < | 1.61E-09 |
| 17 | TBKBP1 | Adipose_Subcutaneous Adipose_Visceral_Omentum Adrenal_Gland Artery_Aorta Artery_Coronary Artery_Tibial Brain_Caudate_basal_ganglia Brain_Cerebellum Brain_Cortex Brain_Frontal_Cortex_BA9 Breast_Mammary_Tissue Cells_Cultured_fibroblasts Cells_EBV-transformed_lymphocytes Colon_Sigmoid Colon_Transverse Esophagus_Gastroesophageal_Junction Esophagus_Mucosa Esophagus_Muscularis Heart_Atrial_Appendage Heart_Left_Ventricle Liver Lung Minor_Salivary_Gland Muscle_Skeletal Nerve_Tibial Ovary Pituitary Prostate Skin_Not_Sun_Exposed_Suprapubic Skin_Sun_Exposed_Lower_leg Small_Intestine_Terminal_Ileum Spleen Stomach Testis Thyroid Uterus Vagina Whole_Blood | < | 1.07E-14 |  | < | 1.47E-09 |
| 17 | AC025682.1 | Adipose_Subcutaneous Adipose_Visceral_Omentum Artery_Aorta Artery_Coronary Colon_Sigmoid Esophagus_Muscularis Heart_Left_Ventricle Minor_Salivary_Gland Testis | < | 5.24E-15 |  | < | 4.19E-09 |
| 17 | AC068234.2 | Adrenal_Gland |  | 2.33E-18 |  |  | 2.68E-09 |
| *The MHC region was excluded from this analysis; Chr: chromosome; PCOS: Polycystic ovary syndrome; SHBG: sex hormone-binding globulin; SHBG_adj_BMI: sex hormone-binding globulin adjusted for BMI; MS: multiple sclerosis. | | | | | | | |
